# Novel antibiotics against *Staphylococcus aureus* without detectable resistance by targeting proton motive force and FtsH

**DOI:** 10.1002/mco2.70046

**Published:** 2025-01-08

**Authors:** She Pengfei, Yang Yifan, Li Linhui, Li Yimin, Xiao Dan, Guo Shaowei, Huang Guanqing, Wu Yong

**Affiliations:** ^1^ Department of Laboratory Medicine The Third Xiangya Hospital of Central South University Changsha Hunan China; ^2^ Department of Laboratory Medicine The Affiliated Changsha Hospital of Xiangya School of Medicine (The First Hospital of Changsha) Central South University Changsha Hunan China

**Keywords:** antibiotic development, methicillin‐resistant *Staphylococcus aureus*, biofilm, proton motive force, FtsH, in vivo

## Abstract

The increased prevalence of methicillin‐resistant *Staphylococcus aureus* (MRSA) and its biofilms poses a great threat to human health. Especially, *S. aureus*‐related osteomyelitis was hardly cured even by conventional antibiotics combined with surgical treatment. The development of novel structural antibiotics is urgently needed. By high‐throughput screening and rational design, we identified a small molecule C218‐0546 and its optimized analog STK848198 with great antimicrobial potential against MRSA avoiding resistance occurrence. And significant synergistical antimicrobial effects were found between the molecules and conventional antibiotics. Mechanisms studies by transcriptomics, fluorescent probes, molecule dynamics, and plasma surface resonance indicated that the proton motive force as well as FtsH are the main potential targets of these molecules. The compounds exhibited excellent in vivo pharmacokinetics, toxicity profiles, and antimicrobial activities in the abscess model as well as the peritonitis‐sepsis model. In addition, STK848198 was found to be effective against MRSA biofilms by interacting with the quorum sensing system. STK848198 also showed in vivo efficacy in the periprosthetic joint infection model. In all, our study identified a class of antimicrobials with novel scaffolds that could be potential alternatives for the treatment of MRSA and its biofilm‐related infections.

## Graphical abstract

• We identified novel structural molecules C218‐0546 and STK848198 that exhibited effective antimicrobial activity without detectable resistance against *Staphylococcus aureus*.

• The antimicrobial effects of the compounds were mainly mediated by proton motive force disruption and FtsH protein inhibition.

• The pharmacokinetic and toxicological profiles of the compounds are excellent, and STK848198 has great therapeutic potential for *S. aureus*‐related bone implantation infections

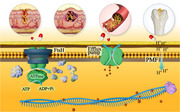

## 1 INTRODUCTION

*Staphylococcus aureus* is a Gram‐positive bacterium that colonizes the nasopharynx and the surface of the skin.^1^ However, it is one of the important pathogens causing community‐acquired and hospital‐acquired infections, which can lead to skin and soft tissue infections, and respiratory or biofilm‐associated infections.^2^ The prevalence rate of MRSA has been increasing, resulting in progressively high mortality.^3^, ^4^ Moreover, vancomycin (VAN)‐mediated/resistant *S. aureus* has also been reported worldwide in recent years.^5^, ^6^ In addition, *S. aureus* is also the main pathogen of high‐resistant biofilm‐related infections on indwelling implants.^7^

*S. aureus* osteomyelitis poses a significant threat to human health, as the coagulase can promote the formation of bone thrombosis and, once combined with local inflammation, it can easily lead to osteonecrosis.^8^, ^9^, ^10^ Implant‐associated osteomyelitis is a global health issue, especially after joint replacement and fracture fixation surgeries^11^, ^12^. *S. aureus* can cause sustained damage, making the disease prone to chronicity.^13^ The biofilm on the implant surface is an important factor in persistent infection.^11^, ^14^ Moreover, the damage caused by *S. aureus* could lead to bone destruction thereby affecting the entire skeletal system.^15^ The recurrence and mortality rates of *S. aureus* osteomyelitis are high.^16^, ^17^ The pathogens are difficult to eliminate even with conventional antibiotics combined with surgical treatments.^18^, ^19^, ^20^

Proton motive force (PMF), also known as transmembrane electrochemical potential gradient, consists of two components: electric potential (ΔΨ) and transmembrane proton gradient (ΔpH).^21^ Bacterial PMF is an energy pathway located in bacterial membranes that plays a crucial regulatory role in various biological processes.^22^, ^23^ Therefore, compounds targeting PMF are potential candidate antibiotics. Antibacterial drugs targeting PMF can be divided into two categories. For example, daptomycin (DAP),^24^ HT61,^25^ Pal‐α‐MSH(6‐13),^25^ and Bacaucin‐1^26^ exert antimicrobial activities by targeting the ΔΨ. While EcDBS1R4,^27^ bile salts,^28^ and flavonoids^29^ exert antimicrobial activity by targeting ΔpH.

FtsH is a membrane‐bound protease composed of two parts: a transmembrane segment located at the N terminus, and its active sites reside in the C‐terminal cytoplasmic domain.^30^ In bacteria, intracellular proteolysis is carried out by ATP‐dependent proteases, belonging to the AAA+ protease family.^31^ Among them, FtsH is the only essential membrane‐anchored protease.^32^ FtsH exhibits molecular chaperon‐like activity that plays a crucial role in protein quality control by eliminating misassembled or misfolded proteins.^33^, ^34^ In *S. aureus*, FtsH degrades a variety of membrane and cytoplasmic proteins and plays multiple roles in stress resistance and virulence production.^35^, ^36^ A previous study reported that the survival rate and bacterial colonization in zebrafish were greatly reduced after being infected with the Δ*FtsH* mutant strain.^37^ Other studies reported that the *FtsH*‐deficient mutant exhibited increased sensitivity to various uncorrelated stressors.^38^ Although, FtsH plays an essential role in the life process of *S. aureus*, antimicrobial targeting FtsH is largely uncovered.

In this study, we reported the discovery and mode of action of two small molecule compounds C218‐0546 and its analog STK848198 against MRSA and its biofilms without detectable resistance. Our study illustrated new antimicrobial mechanisms by these molecules, which own great potential for the treatment of MRSA and its biofilm‐related refractory infections in clinical settings.

## 2 RESULTS

### 2.1 Rational discovery of antimicrobials

**FIGURE 1:**
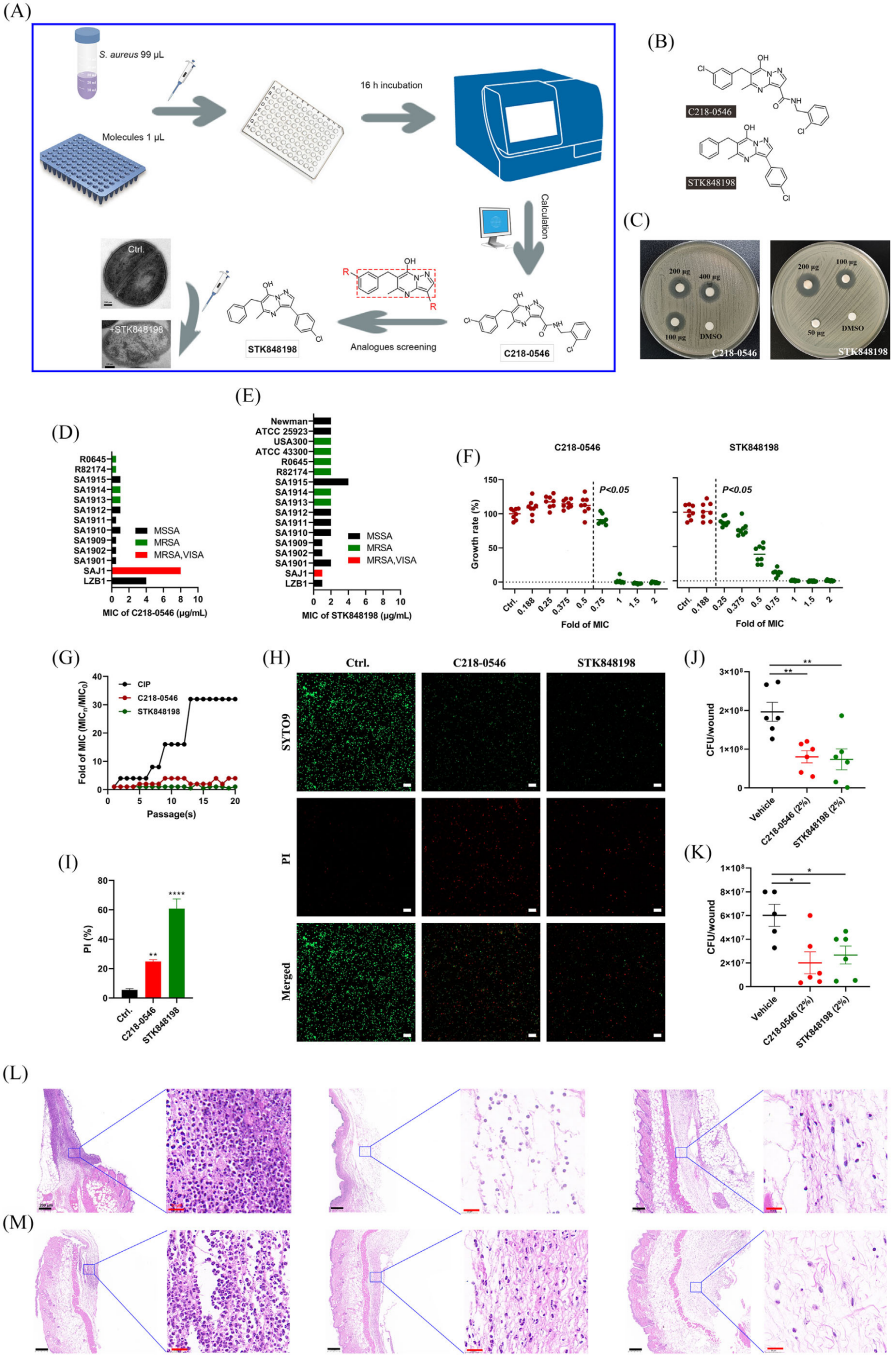
Antimicrobial activity of C218‐0546 and its analogue STK848198 against *S. aureus*. (A) Flow chart of bioactive molecules screening. The C218‐0546 was selected from the MINI Scaffold Library, and the optimized molecule STK848198 was acquired by C218‐0546 scaffold‐related analogs screening. The red dash box indicates the scaffold of C218‐0546. (B) Chemical structure of C218‐0546 and STK848198. (C) Antimicrobial susceptibility of C218‐0546/STK848198 determined by K‐B test. DMSO was used as a negative control. (D) MIC values distribution of C218‐0546 against *S. aureus* clinical isolates. (E) MIC values distribution of STK848198 against *S. aureus*‐type strains and clinical isolates. (F) Concentration‐growth inhibition curves of C218‐0546/STK848198 against *S. aureus* ATCC 43300. (G) The low resistance‐inducing ability of C218‐0546/STK848198 at the sub‐MIC (1/2×MIC) against *S. aureus* ATCC 43300. CIP was used as a positive control. (H) Live/dead bacteria determination after treatment with 1×MIC of C218‐0546/STK848198 for 2 h by SYTOX9/PI staining. Scale: 20 µm. (I) Fluorescence intensity quantification of the dead or impaired cells (PI‐stained) proportion. (J, K) Effective in vivo antibacterial effects of C218‐0546/STK848198 against *S. aureus* ATCC 43300 (J) and ATCC 25923 (K) in a wound infection model. The related H&E staining of the infected areas by ATCC 43300 (L) and ATCC 25923 (M), respectively. *p < 0.05, **p < 0.01, ***p < 0.001, ****p < 0.0001.

By bacterial growth‐based high throughput screening, our previous study discovered the potential antimicrobial activity of small molecular C218‐0546.^39^ To optimize the structure of the molecule, we developed its analog STK848198 by rational design (Figure S1; Figure 1A,B). As shown in Figure S1, to explore the essential groups of C218‐0546, we supposed that the pyrazolo[1,5‐α]pyrimidine was the basic scaffold of this molecule. The substitution of the hydroxyl on the scaffold (K784‐2416) reduced the antimicrobial effects of the molecule with the minimal inhibitory concentration (MIC) >32 µg/mL (the primary MIC value of C218‐0546 was 4 µg/mL). Thus, this hydroxyl is indispensable for the antimicrobial effects of C218‐0546. Next, we reduced the side chain with a chlorine substituent connected to the pyrimidine (K784‐4052), and an enhanced MIC value >32 µg/mL was also observed. We could speculate that the whole or part of the pyrimidine side chains was probably also dispensable for the antimicrobial activity. As we expected, the vanished antimicrobial activity was also found with the substitute of the pyrazolo (STK584829), which indicated whole or part of the side chains on pyrazolo were functional for its antimicrobial ability. Further, we extended the chemical bonds on these side chains (K786‐9860), and a decreased antimicrobial effect was observed with MIC = 8 µg/mL. Thus, we inferred that the long distance of the distal benzene ring connected to the pyrazolo may reduce its antimicrobial activity. So, we speculated that enhanced antimicrobial ability could be achieved by shortening the linking group between pyrazolo and the distal benzene ring. As expected, a more structural simplified molecule (STK848198) with greater antimicrobial effects was developed with MIC = 2 µg/mL. However, more side chains on pyrazolo (STK585953) could reduce the antimicrobial ability of STK848198. Substituent groups on the distal benzene ring (C218‐0864, C218‐0468, C218‐0460, and C218‐0278) connected to the pyrimidine did not optimize the MIC values. The absorption, distribution, metabolism, and excretion/toxicity (ADME/T) prediction by Schrodinger software indicated the great potential of the compounds to be developed as antibiotics with extremely low toxicity (Table S1). Thus, we selected the small molecule compound C218‐0546 and its analog STK848198 for further study.

**FIGURE 2:**
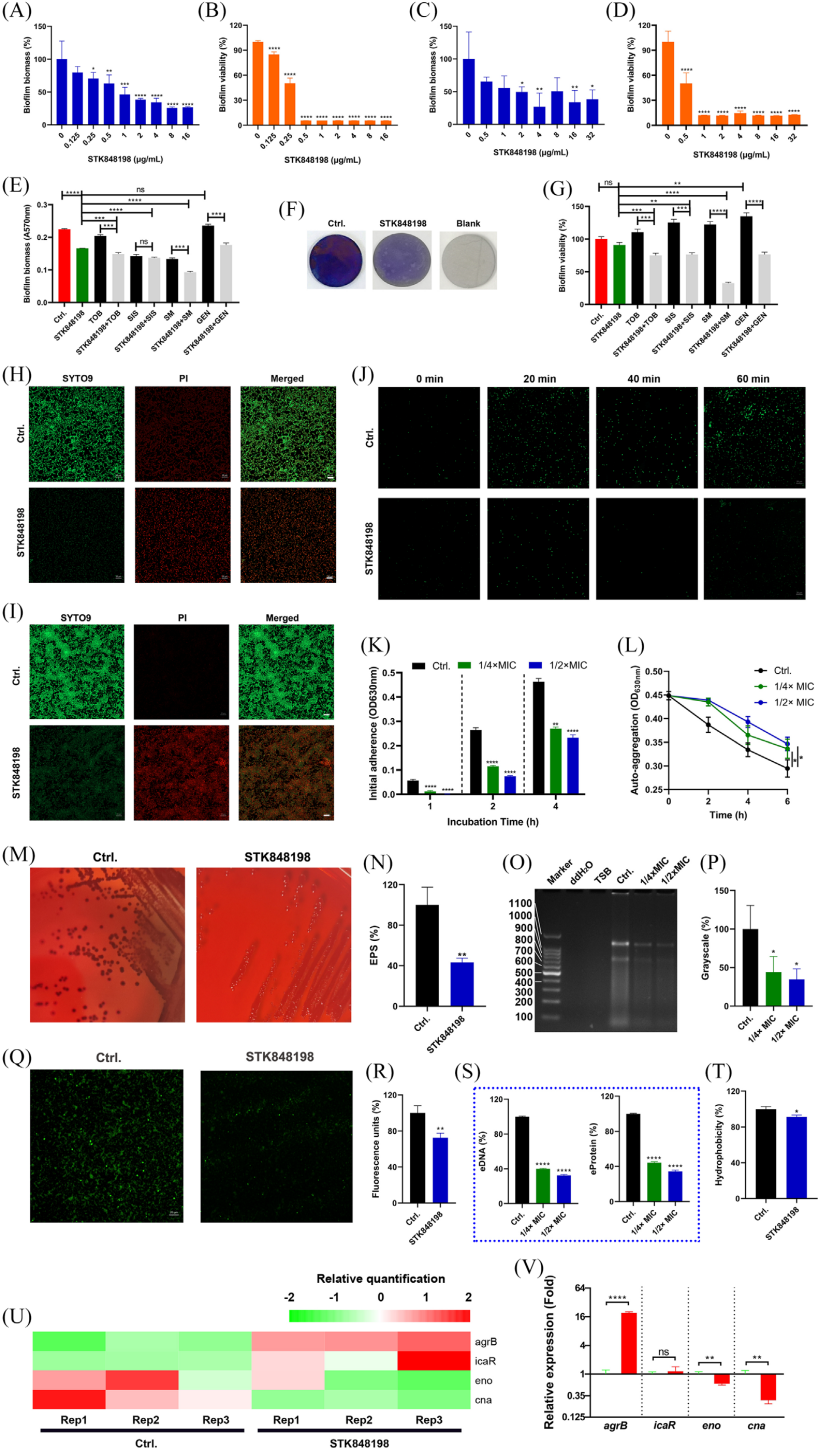
Antibiofilm effects by STK848198 against *S. aureus*. (A, B) Biofilm inhibitory activity of STK848198 against *S. aureus* ATCC 43300 was determined by crystal violet staining (A) and XTT staining (B), respectively. (C, D) Preformed biofilm eradication effects of the compounds against *S. aureus* ATCC 43300 were detected by crystal violet staining (C) and XTT staining (D), respectively. (E) Biofilm eradication by STK848198 (8 µg/mL) alone or in combination with aminoglycosides (32 µg/mL) against *S. aureus* on Ti (titanium)‐discs assessed by crystal violet staining. (F) Representative images of the biofilms formed on Ti‐discs. (G) Ti‐disc‐related biofilm eradication determined by XTT staining. (H) Biofilm inhibitory effects of STK848198 observed by SYTO9/PI staining. The biofilms were treated with STK848198 at the concentration of 1×MIC. (I) Biofilm eradicating activity by 1×MIC of STK848198 assessed by SYTO9/PI staining. (J) Sub‐MIC of STK848198 (1/4× MIC) inhibited the initial adherence of *S. aureus* ATCC 43300 determined by SYTO9 staining. (K) Initial adherence quantification by turbidity method. (L) Auto‐aggregation inhibition by sub‐MIC (1/4‐1/2×MIC) of STK848198. (M) EPS determination in the presence or absence of 1/4× MIC of STK848198 observed by Congo Red agar. (N) EPS production after being treated with 1/4× MIC of STK848198 was determined by the phenol‐sulphoacid method. (O) eDNA production inhibition after being treated with 1/4‐1/2× MIC of STK848198 for 4 h was detected by agarose gel electrophoresis. (P) Grayscale quantification of the eDNA. (Q) Sub‐MIC (1/4× MIC) of STK848198 reduced the eDNA production observed by SYTO9 staining. (R) Fluorescence intensity quantification of the eDNA stained with SYTO9 probe. (S) STK848198 inhibited the eDNA and eProtein production after 4 h treatment detected by spectrophotometric method. (T) STK8484198 reduced the hydrophobicity of *S. aureus* at the concentration of 1/4× MIC. (U) STK848198 inhibited the biofilm formation‐related gene expression by transcriptomic analysis. (V) Relative gene expression quantification by qRT‐PCR. ns: No statistical significance, *p < 0.05, **p < 0.01, ***p < 0.001, ****p < 0.0001.

**FIGURE 3:**
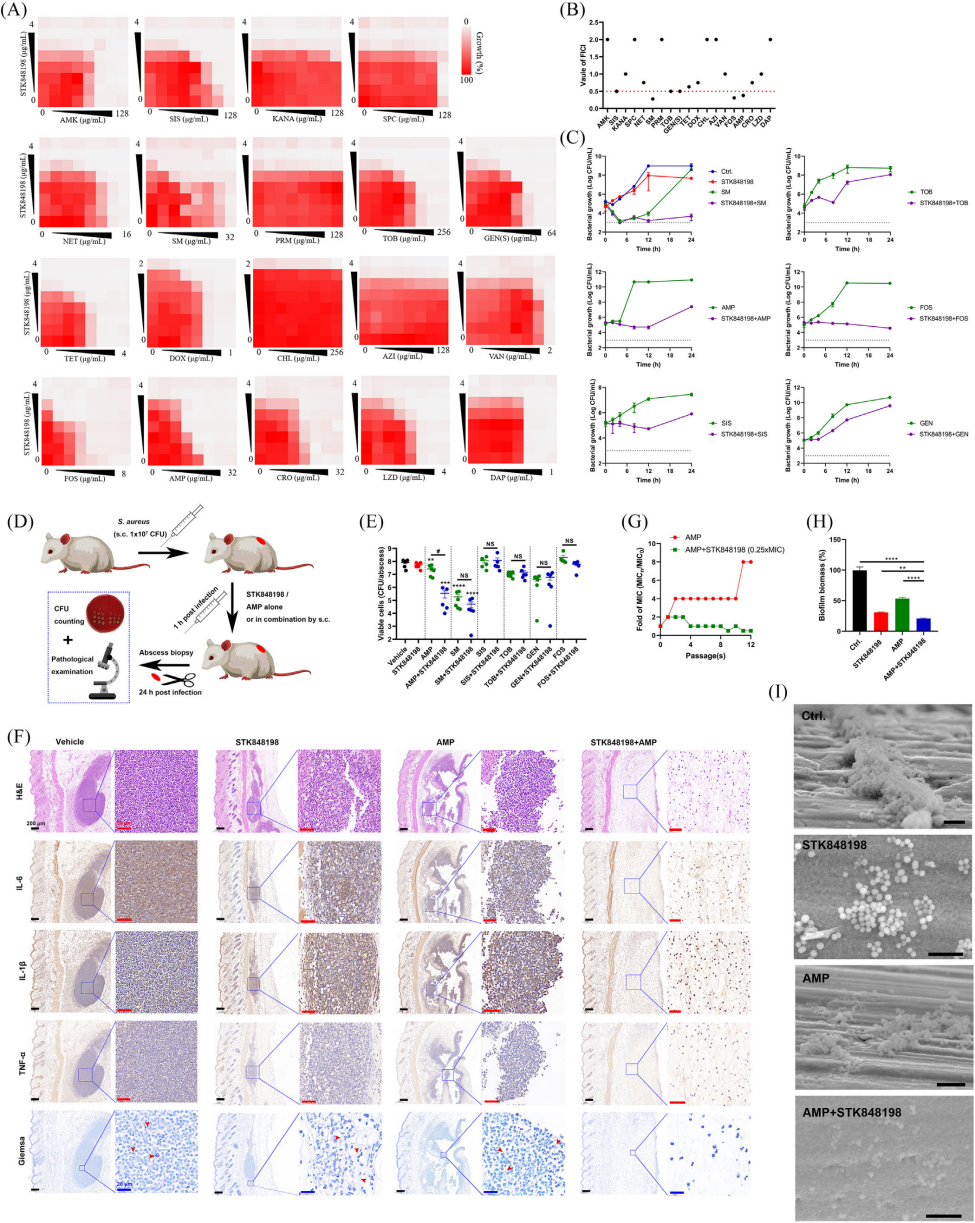
Synergistical antimicrobial effects between STK848198 and AMP. (A) Drug combination between STK848198 and conventional antibiotics determined by checkerboard assay. (B) Distribution of FICI values. (C) Time‐killing curves of drug combinations between STK848198 (0.5 µg/mL) and antibiotics with FICI≤ 0.5 [SM (8 µg/mL)/ TOB (32 µg/mL)/ AMP (2 µg/mL)/fosfomycin (0.5 µg/mL)/SIS (32 µg/mL)/gentamycin (8 µg/mL)]. (D) Diagram for abscess infection model preparation. (E) STK848198 combined with AMP synergistically reduced the viable cell counts in abscess. (F) Reduced pathological change of the abscess after being treated with AMP and STK848198 alone or in combination detected by H&E staining, immunohistochemistry (IL‐6, IL‐1β, and TNF‐α), and Giemsa staining, respectively. (G) STK848198 inhibited the resistance‐inducing ability of AMP. (H) Synergistical biofilm eradication effects between STK848198 (0.5 µg/mL) and AMP (8 µg/mL). (I) Biofilm eradication by STK848198 and AMP alone or in combination was observed by SEM. Scale: 5 µm. *p < 0.05, **p < 0.01, ***p < 0.001, ****p < 0.0001.

The dose‐dependent growth inhibition activity of C218‐0546 and STK848198 was observed by disc diffusion assay (Figure 1C). The MIC values of C218‐0546 and STK848198 against methicillin‐sensitive *S. aureus*, MSRA, VAN intermediate *S. aureus* (VISA), and DAP‐resistant strains (Table S2) ranged from 0.5 to 8 µg/mL (Figure 1D) and 1, 2, 3, 4 µg/mL (Figure 1E), respectively. STK848198 exhibited better antimicrobial activity against VISA (SAJ1) than C218‐0546. Meanwhile, 0.75×MIC of C218‐0546 started to show growth inhibition with a statistical significance, while the growth inhibitory effect of STK848198 was observed at the concentration of 0.25×MIC in a concentration‐dependent manner (Figure 1F). The dose/time‐depended growth inhibitory effects against other *S. aureus* strains were also observed (Figure S2A,B). Although sub‐MIC of ciprofloxacin (CIP) obviously increased the MIC values up to 16–32 fold after 20 days of treatment, sub‐MIC of C218‐0546 and STK848198 hardly induced the resistance occurrence with only 2‐ and 0‐fold of MIC increasement, respectively (Figure 1G; Figure S3B,D). Surprisingly, C218‐0546 and STK848198 still showed effective antimicrobial activities against the CIP‐induced drug‐resistant strains (Figure S3A,C,E). Similarly, no resistance occurrence was also found by on‐step resistance‐inducing assay (Table S3, Figure S3F). Further, by SYTO9/PI staining, both C218‐0546 and STK848198 exhibited effective antimicrobial effects with an increased proportion of PI‐stained (impaired or dead) cells (Figure 1H,I). In addition, the wound infection model also demonstrated the preliminary in vivo anti‐infection activity of the compounds with significantly decreased bacterial cell loads (Figure 1J,K) as well as inflammatory cell infiltration (Figure 1L,M) in the wounds. In addition, the compounds also exhibited dose‐dependent antimicrobial activities against *S. epidermidis* as well as enterococcus (including VAN‐resistant strains) with MIC ranging from 1 to 8 µg/mL (Figure S4A,B, Table S4). However, no antimicrobial activity was found by the compounds against Gram‐negative pathogens with the MIC > 32 µg/mL (Table S4).

**FIGURE 4:**
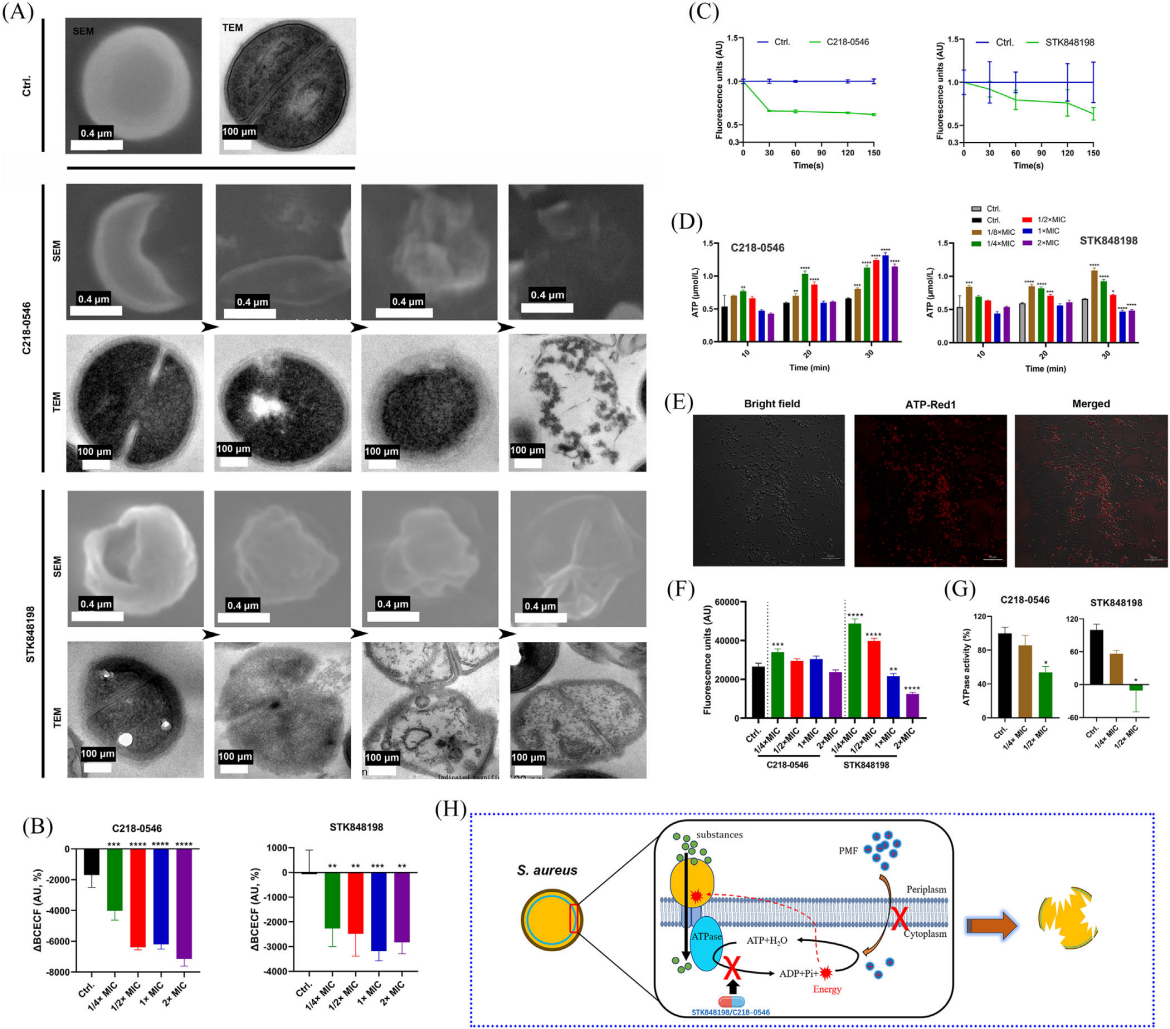
ATPase inhibitory activity by C218‐0546 and STK848198. (A) Ultrastructure observation of *S. aureus* ATCC 43300 by SEM and TEM after being treated with C218‐0546/STK848198 for 1 h. Scales: 4 µm for SEM, and 100 µm for TEM. (B) C218‐0546 and STK848198 disrupted the PMF balance determined by BCECF‐AM probes. (C) C218‐0546 and STK848198 reduced the fluorescence intensity of DiSC3(5). (D) Sub‐MIC of compounds enhanced the accumulation of intracellular ATP. (E) ATP‐Red1 was a sensitive probe for intracellular ATP tracing. (F) Sub‐MIC of the compounds enhanced the fluorescence intensity of ATP‐Red1. (G) ATPase inhibitory activity by sub‐MIC of C218‐0546/STK848198. (H) Diagrammatic sketch of the potential targets by C218‐0546 and STK848198. *p < 0.05, **p < 0.01, ***p < 0.001, ****p < 0.0001.

No hemolysis activity was observed by C218‐0546/STK848198 (Figure S5A). Meanwhile, no cytotoxicity against the cell lines of LO2 (Figure S5B,E) and HepG2 (Figure S5C,E) was found by the compounds up to 64 µg/mL. Although moderate cytotoxicity of C218‐0546 was exhibited against HSF with the IC_50_ of 34.5 µg/mL (which is still much higher than the value of MICs), no toxicity was found by STK848198 against the HSF (Figure S5D,E). Given the skin and soft tissue, infections mainly occurred by *S. aureus*, we further study the cytotoxicity to HSF by Calcein‐AM/PI staining. As shown in Figure S5F, sporadic PI staining cells were observed after being treated with 32 µg/mL of C218‐0546, but almost no impaired cell was found in the presence of 32 µg/mL of STK848198. Similarly, the low apoptosis‐inducing ability was also observed by the cytometer (Figure S5G). Meanwhile, the results of hERG indicated no inhibition activity was exhibited against potassium channels by C218‐0546 and STK848198 with the IC_50_ > 30 µM (Figure S5H,J), although an obvious inhibitory effect by the positive control (cisapride) was found (Figures S5I and S6J). In addition, no genotoxicity of C218‐0546 (Table S5) and STK848198 (Table S6) was found by the Ames test.

### 2.2 Antibiofilm activity

Crystal violet and XTT staining were used to determine the total biofilm biomass and the biofilm viability of *S. aureus*, respectively. STK848198 significantly inhibits the biofilm biomass formation of *S. aureus* at the sub‐MIC concentration of 0.25 µg/mL in a concentration‐dependent manner (Figure 2A; Figure S6A). Meanwhile, the biofilm viability was also effectively inhibited at the concentration of 0.125 µg/mL (Figure 2B). STK848198 could also eradicate the preformed biofilm biomass as well as reduce its viability at the concentrations of 2 and 0.5 µg/mL, respectively (Figure 2C,D; Figure S6C). STK848198 could also be synergy with some of the aminoglycosides (tobramycin [TOB] and streptomycin [SM]) inhibited the *S. aureus* biofilm formation on Ti (titanium)‐discs (Figure 2E). The presentative images of biofilms formed on the Ti‐discs were shown in Figure 2F by crystal violet staining. Moreover, all of the tested aminoglycosides exhibited synergistical eradicating effects with STK848198 against preformed biofilms (Figure 2G). Similarly, the SYTO9/PI staining also exhibited the biofilm inhibitory (Figure 2H) or eradicating (Figure 2I) activity of STK848198 with reduced biofilm biomass or increased proportion of impaired or dead bacterial cells.

As the first step, initial adherence is critical for the biofilm formation.^40^ As shown in Figure 2J, sub‐MIC (1/4×MIC) of STK848198 could effectively inhibit the bacterial cell adherence within 1 h, which further affected the biofilm‐related cell accumulation for the biofilm growth within 4 h (Figure 2K). STK848198 could also inhibit the auto‐aggregation capacity of *S. aureus* (Figure 2L) at the sub‐MIC without influencing the bacterial growth during the experiment (<1 h of incubation, Figure S7). To explore the alteration of biofilm components after the treatment of STK848198, the extracellular polysaccharides (EPS), extracellular DNA (eDNA), and extracellular protein (eProtein) assessments were performed. Conge red staining showed reduced EPS production with dark colonies in the control group and red colonies in the tested group (Figure 2M). The reduced EPS was quantified by the phenol‐sulfuric acid method as well (Figure 2N). Further, the agarose gel electrophoresis (Figure 2O) and its related grayscale quantification (Figure 2P) exhibited decreased eDNA production in the presence of STK848198. For eDNA visualization, we observed the diminished fluorescent intensity of nucleic acid binding dye STYO9 (Figure 2Q,R), and the spectrophotometry quantitatively also showed the reduced production of eDAN and eProtein by STK848198 treatment (Figure 2S). Meanwhile, STK848198 could also inhibit the hydrophobicity (another virulence factor essential for biofilm formation) of *S. aureus* (Figure 2T). By transcriptomic analysis and qRT‐PCR quantification, STK848198 was shown to upregulate the negative regulatory genes of *agrB* while downregulating the component‐related genes of *eno* and *cna* (Figure 2U,V). The biofilm inhibitory effect was also found by C218‐0546 (Figure S8A,B); however, it exhibited no biofilm‐eradicating activity against *S. aureus* (Figure S8C,D). In addition, similar biofilm inhibitory and eradicating activities were observed by STK848198 against *S. epidermidis* (Figure S6B,D).

### 2.3 Drug combination with conventional antibiotics

The combinational antimicrobial effects between C218‐0546 and convention antibiotics were determined by checkerboard assay. The synergy was observed between C218‐0546 and the antibiotics of amikacin (AMK), sisomicin (SIS), netilmicin (NET), SM, and TOB with the FICI ≤ 0.5 (Figure S9A,B). And the synergistic effects were also verified by the time‐killing assay (Figure S9C). Further, the abscess infectious model was used to assess the synergistical antimicrobial effects in vivo. As shown in Figure S9D, only AMK, NET, and SM exhibited statistical differences, and the most significant ΔLog10 was observed between C218‐0546 and AMK (Figure S9E). Similarly, the combination between C218‐0546 and AMK showed more obvious reduced inflammatory cell infiltration and inflammatory factors production (IL‐6, IL‐1β, and TNF‐α) than used alone by hematoxylin–eosin (H&E) staining as well as immunohistochemistry, respectively. The Giemsa staining also exhibited largely reduced bacterial loads in the abscess (Figure S10). In addition, sub‐MIC of C218‐0546 inhibited the resistance occurrence by AMK (Figure S9F).

As for STK848198, the checkerboard assay and its related fractional inhibitory concentration index (FICI) values indicated the synergistical antimicrobial activities between STK848198 and the conventional antibiotics of SM, ampicillin (AMP), SIS, TOB, fosfomycin, and gentamycin (Figure 3A,B). Similarly, the time‐killing curves also exhibited the synergy between STK848198 and the antibiotics at the concentrations of sub‐MIC (Figure 3C). Next, the in vivo synergistical antimicrobial effects were assessed by abscess infection models (Figure 3D). However, as shown in Figure 3E,F, only the group of AMP+STK848198 exhibited synergy against *S. aureus* in vivo (Figure 3E) with obviously reduced inflammatory infiltration, inflammatory factors production as well as bacterial loads counting (Figure 3F). Thus, the combination between AMP and STK848198 was selected for further study. By consecutive (Figure 3G) or one‐step (Figure S11) resistance‐inducing assay, sub‐MIC of STK848198 was found to inhibit the resistance occurrence of AMP. In addition, by using crystal violet staining (Figure 3H) and scanning electron microscope (SEM) observation (Figure 3I), AMP showed enhanced biofilm‐eradicating effects when combined with STK848198.

### 2.4 PMF disruption and aberrant ATP utilization by C218‐0546 and STK848198

Different phased detrimental bacterial cells were observed by SEM and transmission electron microscope (TEM) after being treated with C218‐0546 and STK848198 (Figure 4A). Specifically, as shown by SEM, the cells were undergone through shrinkage to rupture shown by SEM. The enlarged periplasmic space, decreased cell density, or cellular apoptosis were shown in TEM. Overall, STK848198 exhibited stronger antimicrobial activity against *S. aureus* than C218‐0546 with more aberrant cells emerged. Since the cell wall (Figure S12A) and cell membrane (Figure S12B) were not the potential targets by C218‐0546 and STK848198. We supposed that the enlarged periplasmic space, wrinkled cells, and cell contents extrusion were owning to disruption PMF, which is the main factor maintaining the osmotic pressure balance and cellular homeostasis.^41^ Thus, we used the probe BCECF‐AM for PMF quantification. As shown in Figure 4B, both compounds exhibited dose‐dependent PMF disruption activity. Meanwhile, the components of PMF were detected by DiSC3(5). A decreased fluorescence intensity by C218‐0546 or STK848198 indicated the ΔΨ disruption by the compounds (Figure 4C). As we expected, the antimicrobial activities of the compounds were changed in the presence of varying proton concentrations (Figure S13). Enhanced reactive oxygen species (ROS) production was observed by the compounds (Figure S12C), which was also connected with the PMF disruption. Noticeably, the intracellular ATP was significantly increased after being treated with sub‐MIC of the compounds (Figure 4D). Since the ATP synthesis was driven by PMF,^42^ we supposed that the compounds could largely inhibit the ATP utilization. Similarly, the accumulated intracellular ATP in the presence of sub‐MIC was also observed by ATP‐Red1 tracing (Figure 4E,F). As we expected, both compounds showed significant inhibitory effects against ATPase (Figure 4G). Overall, C218‐0546 and STK848198 disrupt the PMF balance and inhibit ATP utilization probably by interacting with ATPase (Figure 4H).

### 2.5 FtsH was a potential target of C218‐0546 and STK848198

**FIGURE 5:**
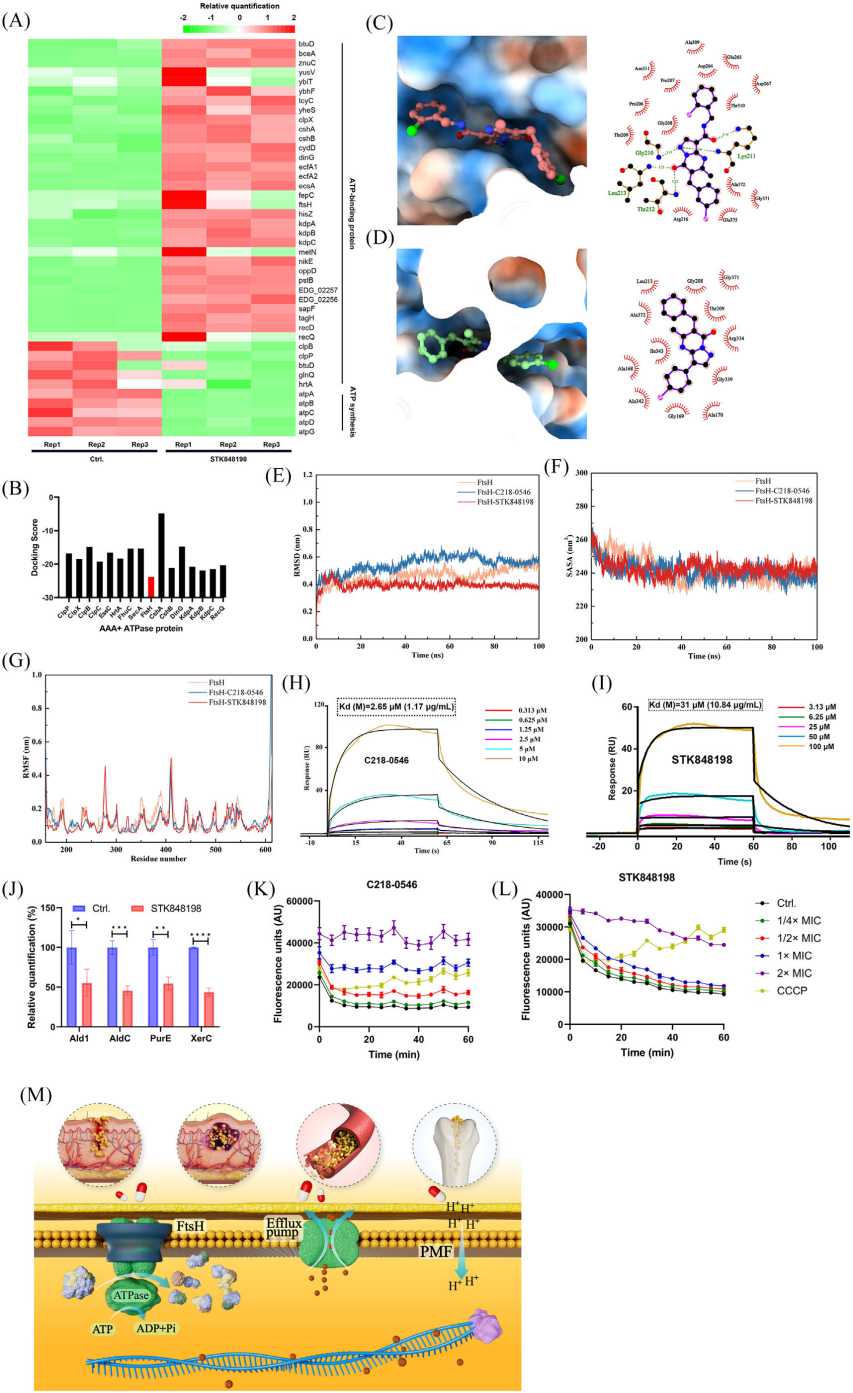
AAA+ ATPase FtsH was a potential target by C218‐0546 and STK848198. (A) The down‐/upregulated ATPase‐related genes after being treated with STK848198 by transcriptomic analysis. (B) FtsH exhibited the lowest docking score among significantly changed ATPase. (C, D) 3D binding model between C218‐0546 (C) or STK848198 (D) and FtsH by molecular docking. (E) RMSD by MD. (F) SASA by MD. (G) RMSF by MD. (H, I) Time–response curves of C218‐0546 (H) or STK848198 (I) interacted with FtsH by SPR. (J) Significantly changed substrate proteins of FtsH. (K, L) The efflux pumps inhibition activity by C218‐0546 (K) or STK848198 (L). (M) Mechanism hypothesis diagram. C218‐0546 and STK848198 exhibited antimicrobial activity against *S. aureus* mainly by the disruption of PMF as well as the inhibition of FtsH and efflux pumps. *p < 0.05, **p < 0.01, ***p < 0.001, ****p < 0.0001.

Transcriptomic analysis exhibited that STK848198 widely upregulated the expression of ATP‐binding proteins, (*ftsH*, *clpP*, and *kdpA*, etc.), while downregulated ATP synthase‐related genes (*atpA*, *atpB*, and *atpC*, etc.; Figure 5A). Thus, we inferred that STK848198 may inhibit the activity of ATP‐binding proteins, thereby negatively feedback the upregulation of their related genes. Meanwhile, inhibition of the ATP‐binding proteins may impair the utilization of ATP, and the intracellular ATP accumulation inhibits the expression of ATP synthase‐related genes. Due to the conservation of the catalytic domains of different ATP‐binding proteins for ATP hydrolysis, the action site of STK848198 may be located in the ATPase domain of the ATP‐binding proteins. Next, we screened the potential target of STK848198 to ATP‐binding proteins by molecular docking (Figure S14A). As shown in Figure 5B, FtsH exhibited the lowest docking score with the best affinity to STK848198 among all the ATP‐binding proteins. As we expected, both the C218‐0546 and STK848198 showed a high affinity with FtsH. In detail, C218‐0546 mainly binds to the hydrophilic cavity of FtsH with widely formed hydrogen bonding to the surrounding amino acid residues of Gly210, Lys211, Thr212, and Leu213. While the benzene rings at the ends of C218‐0546 formed hydrophobic interactions with surrounding amino acids (Figure 5C). STK848198 mainly bound to the hydrophobic cavity of FtsH, and the widely formed hydrophobic interactions were exhibited with the surrounding amino acids residues of Ala168, Ala170, Leu213, Ile343, Ala372, etc (Figure 5D). By homology analysis, FtsH exhibited high similarity with PBD: 2DHR (Figure S14B). Thus, homology modeling was conducted based on this (Figure S14C), and the active site of the FtsH was determined (Figure S14D,E). Further, by molecule dynamics (MD) simulation, root‐mean‐square deviation (RMSD) values of the FtsH or its combination with molecules showed fluctuations within 20 ns of the MD simulation. Subsequently, the RMSD values reached a relatively stable state with average values of 0.49 ± 0.046 nm, 0.58 ± 0.029 nm, and 0.39 ± 0.014 nm at 60 ns for FtsH, FtsH‐C218‐0546, and FtsH‐STK848198, respectively (Figure 5E). Similarly, fluctuations of the solvent accessible surface area (SASA) values were also observed within 40 ns, which is mainly due to the strong and unstable interaction between the proteins and surrounding water molecules in the early stage of the simulation. But the interaction gradually stabilized after 60 ns with the SASA values of 237.73 ± 3.42 nm^2^, 238.56 ± 3.23 nm^2^, and 241.86 ± 2.77 nm^2^ for FtsH, FtsH‐C218‐0546, and FtsH‐STK848198, respectively (Figure 5F). After the compound's binding, there was a significant decrease in the flexibility of the Asn350 to Glu400 region in the FtsH protein, which is just located in the binding site (Figure 5G). Overall, the MD results indicated that the binding of small molecules to FtsH protein could reach a stable state. Further, FtsH was synthesized and purified (Figure S15), and surface plasmon resonance (SPR) was used to explore the affinity in vitro. As shown in Figure 5H,I, both the C218‐0546 and STK848198 exhibited obvious responses in the presence of FtsH with the Kd values of 1.17 and 10.84 µg/mL, respectively. Meanwhile, the reduced production of FtsH catalytically generated proteins (Ald1, AldC, PurE, and XerC) after STK848198 treatment also validated the inhibitory effects of the compounds at the cellular level (Figure 5J). In addition, we detected the interaction between C218‐0546/STK848198 and efflux pumps. As shown in Figure 5K,L, both the compounds showed more effective inhibitory activities against efflux pumps than the positive control CCCP. Given that the efflux pumps are also ATP‐binding proteins,^43^ we could conclude that although strong affinity was detected between C218‐0546/STK848198 and FtsH, the compounds may also widely inhibit the function of some other ATP‐binding proteins, which finally lead to the cell death (Figure 5M).

The transcriptomic analysis also indicated that STK848198 could also inhibit the Fe(3+)‐acquisition‐related gene expression (Figure S16A) and protein production (Figure S16B). Given the fact that iron metabolism may be correlated with ATP depletion and ROS production, we further explore the antimicrobial activity of STK848198 by the addition of ion chelating agent (Figure S16C) or by the addition of exogenous iron ions (Figure S16D). However, no significant interaction between STK848198 and these agents was found, which could be due to the compensation mechanism by other pathways.

### 2.6 in vivo antimicrobial efficacy in sepsis and periprosthetic joint infection models

**FIGURE 6:**
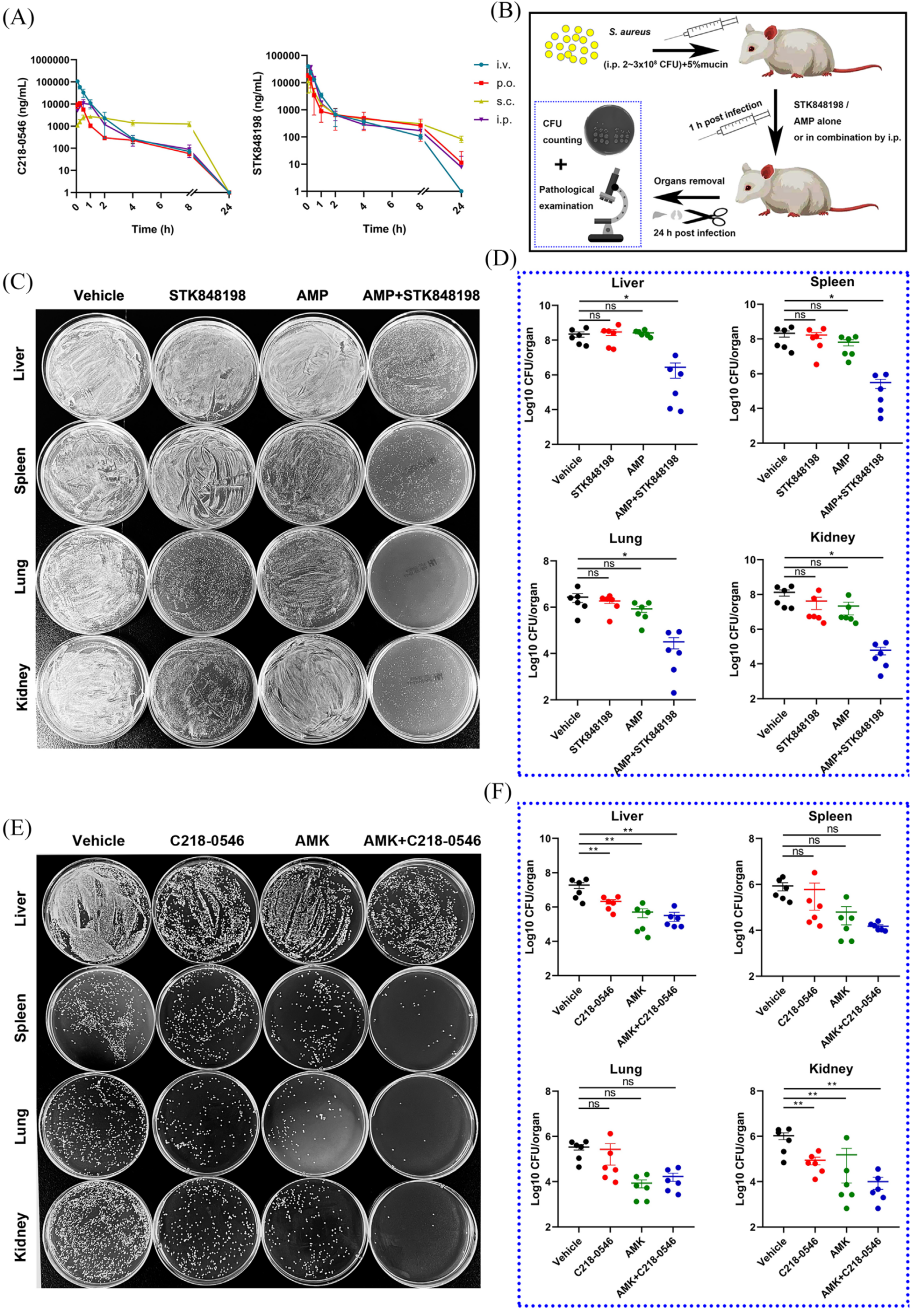
In vivo antimicrobial efficacy of C218‐0546 and STK848198 in a peritonitis‐sepsis model. (A) Pharmacokinetic curves of C218‐0546 and STK848198. (B) Diagram for peritonitis‐sepsis model preparation. (C) Representative images of the synergistical antimicrobial effects between AMP and STK848198 by CFU counting. The mice were inoculated with 3.5 McF of *S. aureus* ATCC 43300 by i.p. injection and treated with 30 mg/kg of STK848198 alone or in combination with 25 mg/kg of AMP. (D) AMP combined with STK848198 effectively reduced the viable bacterial cell loads in mice organs. (E) Representative images of the synergistical antimicrobial effects between AMP and C218‐0546. The mice were inoculated with 2.5 McF of *S. aureus* and treated with 30 mg/kg of C218‐0546 alone or in combination with 25 mg/kg of AMP. (F) The viable bacterial cell loads in mice organs after being treated with AMP and C218‐0546 alone or in combination. *p < 0.05, **p < 0.01, ***p < 0.001, ****p < 0.0001.

Both the C218‐0546 and STK848198 exhibited high plasma protein binding rate (Figure S17A) and stabilization in the presence of liver microsomes in vitro with *T*_1/2_ of 128.33 and 43.58 min, respectively (Figure S17B,C). Further, the pharmacokinetic parameters also indicated good in vivo performance in mice (Figure 6A; Tables S7 and S8). The blood drug concentration‐time curves indicated the half‐life of C218‐0546 and STK848198 were 0.98–2.38 h and 2.54–7.65 h, respectively, by all the administration routes. The quick effects could be reached with the low *T*_max_ of 0.08–2.33 h and 0.08–0.33 h for C218‐0546 and STK848198, respectively. Except for C218‐0546 by the administration route of s.c. with the *C*_max_ of 2.8 µg/mL, all other groups showed a varying *C*_max_ between 10.91 and 106.48 µg/mL, which were obviously higher than the values of MIC. The bioavailability of p.o., s.c., and i.p. were 23.4, 27.57, and 52.37 for C218‐0546 and 40.42%, 40.89%, and 87.02% for STK848198, respectively. Thus, we further study the in vivo systemic antibacterial activity of the compounds by i.p. injection administration due to its highest bioavailability, ideal peak concentration, and convenient operation.

Since the obvious in vitro synergistic antimicrobial effects between C218‐0546/STK848198 and AMP/AMK, respectively, as described above. The combinations were further determined in vivo by peritonitis‐sepsis models. The models were prepared by i.p. administration with *S. aureus* (2 × 10^8^ CFU and 3 × 10^8^ CFU for C218‐0546 and STK848198 treatment, respectively, due to the stronger in vitro antimicrobial efficacy of STK848198 than C218‐0546 and which was optimal for the observation of combinational effects) in the presence of 5% mucin. After administration (i.p.) with the compounds alone or in combination with antibiotics, the bacterial loads in organs were counted at 24 h postinfection (Figure 6B). Although single use of STK848198 or AMP showed no statistical difference compared with the vehicle group, the drug combination significantly reduced the bacterial loads in all the organs of the liver, spleen, lung, and kidney (Figure 6C,D). Differently, C218‐0546 alone or in combination with AMK showed no statistically different bacterial loads in the spleen and lung but obviously reduced bacterial loads were observed in the liver and kidney, which could be due to the varied organ distribution ability of the compound (Figure 6E,F).

**FIGURE 7:**
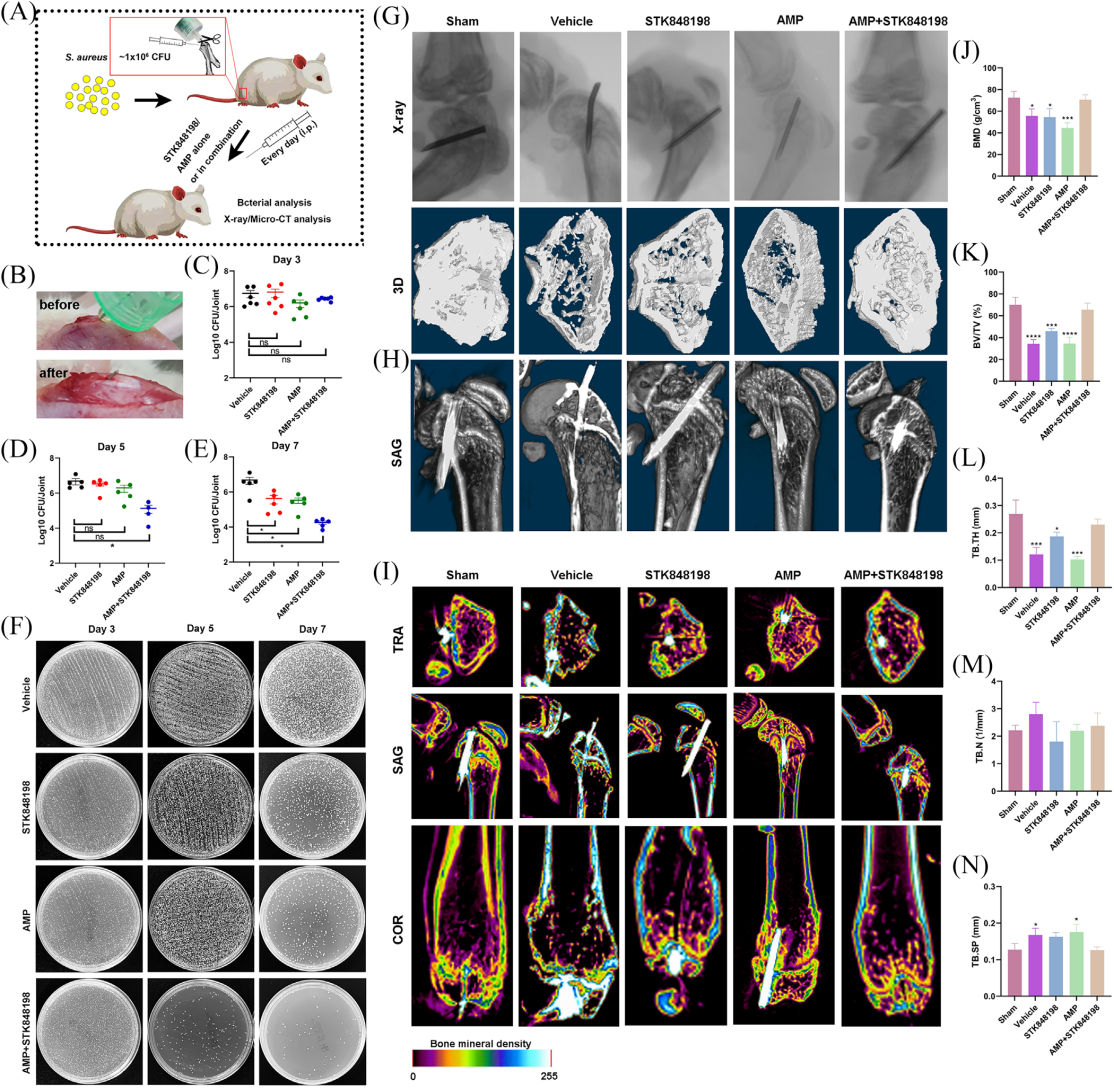
Effective in vivo antimicrobial efficacy of C218‐0546 and STK848198 in a periprosthetic joint infection (PJI) infection model. (A) Diagram for PJI infection model preparation. (B) Representative images of Ti needle implantation. (C–E) The viable cells counting of the infected joints after treated with AMP alone or in combination with STK848198 for 3 days (C), 5 days (D), and 7 days (E), respectively. (F) Representative images of the viable cells by CFU counting after being treated with the compounds. (G, H) The X‐ray (G) and micro‐CT images [including 3D reconstruction and sagittal (SAG) observation (H)] of the infected joints with implants 14 days postinfection. (I) Bone mineral density heat‐map of the infected joints 14 days postinfection. TRA, transverse. COR, coronal. (J–N) Quantitative analysis of bone tissue density‐related parameters, including bone mineral density (J), bone volume/total volume (K), trabecular thickness (L), trabecular number (M), and TB.SP (N). *p < 0.05, **p < 0.01, ***p < 0.001, ****p < 0.0001.

Next, due to the strong antimicrobial and antibiofilm activities of STK84898, we assessed the in vivo antimicrobial ability of STK848198 by the periprosthetic joint infection (PJI) model. The PJI model was prepared by implanting an insulin needle in the femur from the knee joint. Meanwhile, the prolonged needle into the knee joint cavity could also simulate the sustained tissue damage caused by joint implants (Figure 7A,B). Although no significant antimicrobial activity was found on day 3 by CFU counting (Figure 7C,F), AMP combined with STK848198 could effectively reduce the bacterial viable cell loads on day 5 (Figure 7D,F). Moreover, STK848198 and AMP alone or in combination could also reduce the bacterial loads in the joint and its surrounding tissue at day 7 (Figure 7E,F). Subsequently, pathological changes in the infected joint were detected by using X‐ray and micro‐CT, respectively. As shown in Figure 7G, X‐ray images indicated that all the insulin needles were successfully implanted into the femur. The 3D reconstruction exhibited that the joint surface of the combinational treated group healed well which was quite close to the sham group. Meanwhile, the sagittal section of the micro‐CT further demonstrated the appropriate depth and position of the implantations (Figure 7H). Bone density analysis revealed that the vehicle group showed a relatively low bone density, while higher density was observed in the combinational treated group (Figure 7I). In addition, a quantitative analysis of the bone density and trabecular recovery was performed. Although no significant change was observed by trabecular number analysis (Figure 7M), the parameters of bone mineral density (Figure 7J), bone volume/total volume (Figure 7K), trabecular thickness (Figure 7L), and trabecular separation/spacing (Figure 7N) showed no statistical difference compared with the sham group after 14 days of combinational treatment with AMP and STK848198, indicating well‐healed bone tissues at the injury sites.

**FIGURE 8:**
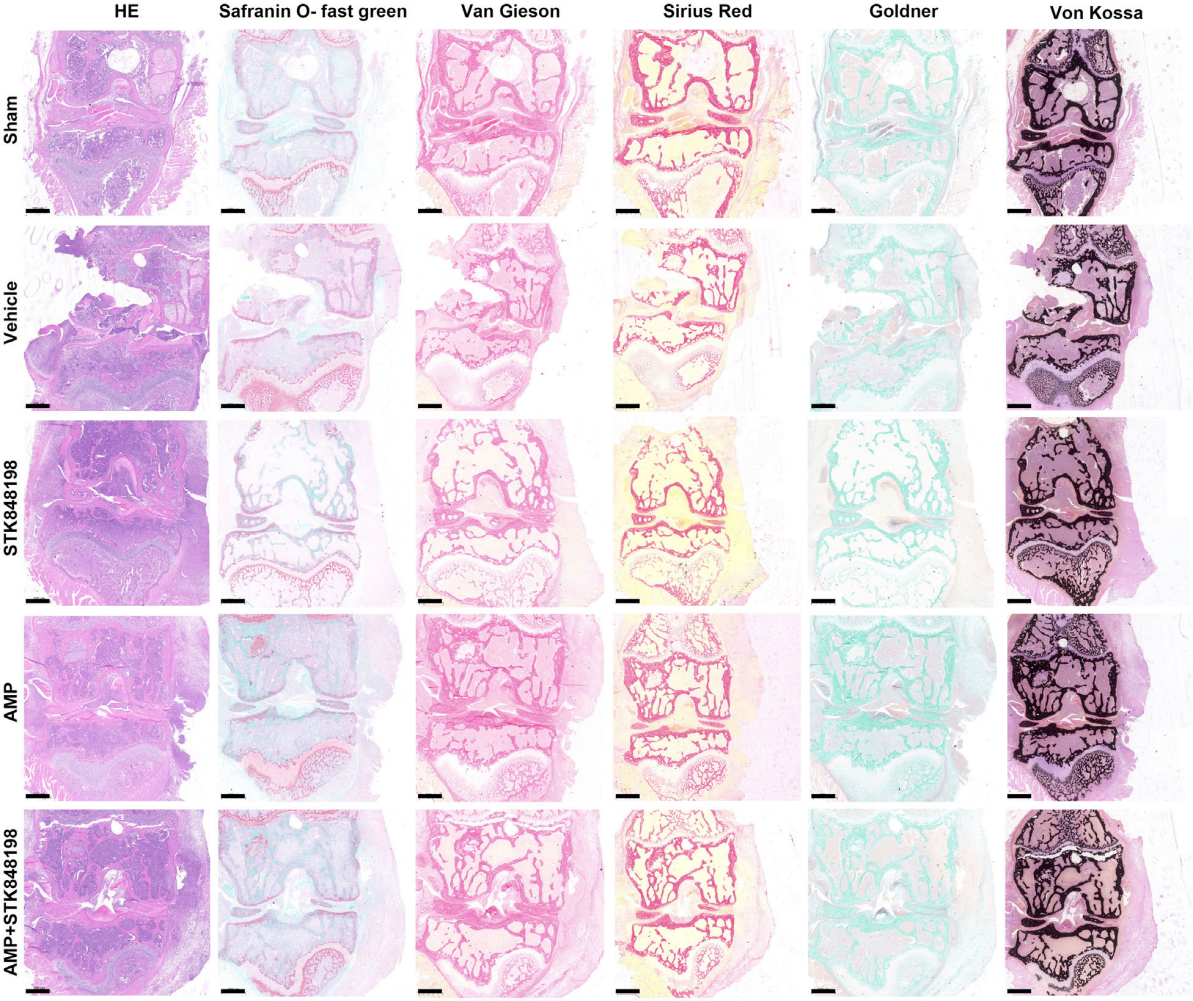
Pathological analysis of the implants and surrounding tissues at day 14. Scale: 500 µm.

In addition, the pathological analysis of the PJI was performed. As shown in Figure 8, the H&E staining showed that the joint morphology of the sham surgery group was relatively intact, whereas the joint junction of the vehicle group was damaged with lots of inflammatory cell infiltration in the joint cavity. The joint structure of the STK848198 monotherapy group was somewhat intact, but there was also inflammatory infiltration. There was a significantly reduced inflammatory infiltration in the AMP monotherapy group. Similar to the sham group, the joint structure of the combinational treatment group was relatively intact without obvious inflammatory infiltration. Safranin O‐fast green staining was used to observe the structure of articular cartilage, subchondral bone, and bone tissue. Compared with the sham group, the cartilage and osteogenesis of the vehicle group were significantly damaged. Although the monotherapy of STK848198 exhibited ineffectively in increasing the osteogenic ratio, the bone tissue morphology of the AMP monotherapy or STK848198/AMP combination group was basically normal, and the osteogenic ratio was similar to that of the sham group. By Van Gieson staining, it can be seen that the sham group showed abundant and colored collagen fibers, whereas the vehicle group only showed significant bone tissue damage with a decreased collagen fiber content. Although, STK848198 alone cannot improve the content of collagen fibers in bone tissue, its combination with AMP largely promoted collagen fiber regeneration. Similarly, the Sirius Red staining also showed that the sham group was normal with abundant collagen fibers, while the vehicle and STK848198 group presented a decrease in collagen fiber content. Although the AMP group exhibited some improvement, the stained collagen fiber in the combinational group was almost close to the sham group. By Goldner staining, the proportion of mineralized bone in the bone tissue was low in the Vehicle group and STK848198 group, while high in the sham group, AMP group, and combinational group. In addition, it can be seen from the Von Kossa staining that the calcium salt deposition in the bone tissue of each group was relatively abundant. This could be due to the fact that the deposition of calcium salts often takes a long time, the experimental period of this model may not be sufficient to observe the changes in calcium salt deposition.

C218‐0546 and STK848198 are well‐tolerant in vivo. First, Masson staining exhibited almost no collagen fiber alteration after treatment with the compounds by s.c. injection compared with the blank group (Figure S18), which could probably indicate the minimal toxicity in the abscess infection model. Meanwhile, the mice were tolerant to the compounds even up to 100 mg/kg (Figure S19A). Then, the acute in vivo toxicity was assessed by i.p. injection with a dose of 30 mg/kg (the dosage used for pharmacodynamics determination) of C218‐0546 or STK848198. No significant difference in erythrocyte parameters (Figure S19B), leukocyte parameters (Figure S19C), platelet counting (Figure S18D), liver biomarker glutamic‐pyruvic transaminase (ALT; Figure S19E), kidney biomarker blood urea nitrogen (Figure S19F) and cardiomyocyte biomarker creatine kinase (Figure S19G) was found between C218‐0546/STK848198 and the vehicle group. Consistently, no pathological change was observed in the myocardium, liver, spleen, lung, and kidney by H&E staining (Figure S19H). Even after 7 days of consecutive treatment with the compounds by i.p. injection, the body weight of the mice showed no difference compared with the vehicle group (Figure S20A). Except for the moderate increase of ALT by C218‐0546 treatment, all the other parameters, biomarkers, and organ‐related H&E staining also showed no difference between the C218‐0546/STK848198 and vehicle group (Figure S20B,C).

## 3 DISCUSSION

*S. aureus* biofilm is highly resistant to conventional antibiotics and can cause various infections.^44^, ^45^ The present study found that the small molecule STK848198 could effectively inhibit the formation of *S. aureus* biofilm at the concentration of sub‐MIC without influencing bacterial proliferation. Therefore, STK848198 may act on the regulatory system related to biofilm formation rather than directly killing the bacterial cells. STK848198 was found to significantly upregulate the negative regulatory gene *agr* in biofilm at the concentration of sub‐MIC while downregulating the biofilm components‐related genes *eno* and *ena*. As widely reported, *agr* is a quorum sensing system‐related gene and is a major gene in regulating various virulence factors production in *S. aureus* which includes biofilm formation.^46^ Similar to our study, many studies have also reported the antibacterial drugs targeting the Agr quorum sensing system in recent years. For example, Wang et al.^46^ found that Solonamide, Cochinmicin, and HQNO exhibited antibiofilm effects by competitively inhibiting the binding of AgrC protein. Zhang et al.^47^ reported that Naringenin could reduce the expression of *agrA* and *hla* genes and hence inhibit biofilm formation. Sully et al.^48^ found that the small molecule Savirin can inhibit the expression of *S. aureus* virulence by targeting the AgrA protein.

In this study, the small molecule compound STK848198 not only targeted the efflux pumps but also disrupted the balance of PMF between the cell membranes. The efflux pumps can expel antibiotics from the inner side of bacteria to the outside, finally leading to bacterial resistance.^49^ Studies have reported that efflux pumps are closely related to the formation of bacterial biofilms.^50^ Therefore, STK848198 is expected to become an ideal efflux pump inhibitor, enhancing the antibacterial activity of conventional antibiotics, reducing the occurrence of drug resistance, and meanwhile treating biofilm‐related diseases.^51^ Similarly, Stokes et al.^52^ found that halicin, a drug for diabetes therapy, can play a broad‐spectrum antibacterial activity by disrupting the transmembrane ΔpH. Dombach et al.^53^ found that the small molecule JD1 can effectively kill MRSA and its persister cells by disrupting transmembrane ΔΨ. Another high‐throughput screening experiment found that the small molecular compounds nordihydroguaiac acid, gossypol, trifluoperazine, and amitriptyline exerted bactericidal activity by disrupting the transmembrane ΔpH or ΔΨ.^54^ The DiSC3(5) fluorescent probe is often used to distinguish whether the antibiotics target transmembrane ΔpH or ΔΨ. Similar to our results, dihydroguaiac acid and gossypol were found to significantly reduce the fluorescence intensity of DiSC3(5), and their main mechanism was also mediated by the disruption of transmembrane ΔpH. However, trifluoperazine and amitriptyline could increase the fluorescence intensity of DiSC3(5), which is mainly mediated by disruption of the transmembrane ΔΨ.^54^ In addition, our previous study also found that eltrombopag could inhibit MRSA growth by disrupting PMF.^55^

Interestingly, efflux pumps are ATPase proteins,^56^ and the maintenance of PMF also depends on ATPase.^57^ Thus, we infer that the ATPase proteins could be potential targets for C218‐0546 and STK848198. We found that STK848198 can extensively upregulate the expression of AAA+ ATPase genes and downregulate the ATP synthase‐related genes. Thus, we supposed that the compound may exert inhibitory activity against the ATP‐binding conserved domain of these AAA+ ATPase, thereby upregulating the expression of these ATPase genes by negative feedback. Due to the compound targeting the ATPase proteins, which could inhibit the utilization of intracellular ATP, the accumulated ATP could also inhibit the expression of ATP synthesis‐related genes by negative feedback. Subsequently, through molecular docking‐based screening, we found that STK848198 has the best potential binding affinity to the AAA+ ATPase protein FtsH. It was further confirmed through PSR and proteomics. As previously reported,^58^, ^59^ ATP‐binding proteins play important roles in bacterial proliferation, cell division, signal transduction, stress response, and cell membrane homeostasis.^60^, ^61^ In addition, the Fts family proteins are also crucial in Gram‐negative bacteria.^60^, ^62^ But, C218‐0546 and STK848198 exhibited no antibacterial effect on them. This could probably be due to the outer membrane barrier by the Gram‐negative bacteria, which prevents small molecules from penetrating the bacterial cell and exerting their effects.

*S. aureus*‐associated osteomyelitis often leads to local inflammation, abscess, invasive osteolysis, and loosening of sepsis implants.^63^ Local medication and surgical treatment cannot be completely cured.^64^ Studies have reported that a biofilm can form on the surface of bone implants after being infected with *S. aureus* and hence spread within and around the bone tissues within 7 days. Thus, this model can reflect the chronic infections related to implants.^65^ Our study found that the combination of STK848198 and AMP significantly reduced the bacterial loads in bone tissues after only 3 days of treatment. And the combination can also effectively reduce the inflammatory infiltration in bone tissues and promote bone tissue recovery at 14 days postinfection. In addition, C218‐0546 and STK848198 were also shown to inhibit inflammatory progress by decreasing the production of IL‐1β, IL‐6, and TNFα, while enhancing the production of IL‐10 (Figure S21), which could also be the underlying mechanism of the rapid healing in infection models in vivo after treatment with the compounds.

C218‐0546 and STK848198 have significant antibacterial activity against *S. aureus* without detectable resistance. Their in vivo pharmacokinetic and toxicological profiles are excellent, and STK848198 has great therapeutic potential for *S. aureus*‐related bone implantation infections. Mechanism studies have shown that these compounds can exert antibacterial effects by inhibiting the efflux pumps and PMF and by specifically targeting the FtsH protein. Therefore, C218‐0546 and STK848198 are expected to become an alternative treatment for refractory chronic *S. aureus* implant‐related infections. Although STK848198 targeting *S. aureus* quorum sensing system has great potential for application in clinical settings, there are also some inevitable drawbacks. For example, almost no small molecule can simultaneously target all four types of Agr quorum sensing systems. These systems are interrelated with replaceable bypasses. In addition, the small molecules targeting *S. aureus* Agr system may also have a certain impact on the normal Staphylococcus flora.

## 4 MATERIALS AND METHODS

### 4.1 Chemicals, strains, cell lines, and culture conditions

*S. aureus* ATCC 29213, ATCC 25923, ATCC 43300, USA300, and *Acinetobacter baumannii* ATCC 19606 were purchased from the American Type Culture Collection (ATCC). *S. aureus* Newman and RJ‐2 were given by Min Li (Shanghai Jiaotong University, Shanghai, P.R. China). *S. epidermidis* RP62A and ATCC 12228 were kindly provided by Di Qu (Shanghai Medical College, Fudan University). *Escherichia coli* ATCC 25922, *Enterococcus faecalis* ATCC 29212, and *Klebsiella pneumoniae* ATCC 700603 were provided by Juncai Luo (Tiandiren Biotech). *Pseudomonas aeruginosa* PAO1 was provide by Minqiang Qiao (College of Life Sciences of Nankai University). The clinical strains were collected from the Third Xiangya Hospital of Central South University and dual identified by VETIK 2 Compact (bioMerieux) and matrix‐assisted laser desorption ionization‐time of flight mass spectrometry (MALDI‐TOF). All strains were stored at −80°C.

Enterococcus strains were grown in brain‐heart infusion broth. Staphylococci strains were cultured in trypsin soybean broth while other Gram‐negative bacteria were cultured in Luria‐Bertani broth. All media were autoclaved at 121°C for 15 min before use. All bacteria were propagated at 37°C 200 rpm. Cell lines of LO2, HepG2, HSF, and RAW264.7 were cultured in RPMI medium or Dulbecco's modified Eagle's medium supplemented with 10% fetal bovine serum. The compounds C218‐0546 and STK848198 were synthesized by WuXi AppTec. Other compounds were mainly purchased from MedChem Express. For in vitro experiments, all compounds were prepared according to the reagent vendor's instructions. For in vivo experiments, compounds were formulated using a mixture of Cremophor EL and ethanol in a ratio of 1:1 (vol/vol).

### 4.2 Animal experiments

All experimental designs and animal procedures were approved by the Ethics Committee of the Third Xiangya Hospital of Central South University (no: CSU‐2022–0599) and in compliance with the NIH Guide for Care and Use of Laboratory Animals guidelines. Female, 6–8‐week‐old ICR mice were obtained from Hunan SJA Experimental Animal Co. Ltd. The construction methods of in vivo infection models are shown in the Supporting Information.

### 4.3 Statistical analysis

All the statistical analysis was performed by GraphPad Prism 8.0 software. Student's *t*‐test was used to analyze the data for two‐group comparisons and one‐way analysis of variance was used to analyze the data for multiple groups. *p* < 0.05 was considered a statistically significant difference.

## AUTHOR CONTRIBUTIONS

She Pengfei and Wu Yong participated in the planning of this study. She Pengfei, Yang Yifan, and Li Linhui performed most of the experiments. She Pengfei, Wu Yong, Li Yimin, Xiao Dan, Guo Shaowei, and Huang Guanqing analyzed the data and wrote the manuscript. All authors have read and approved the final manuscript.

## CONFLICT OF INTEREST STATEMENT

The authors declare no conflict of interest.

## ETHICS STATEMENT

All experimental designs and animal procedures were approved by the Ethics Committee of the Third Xiangya Hospital of Central South University (no: CSU‐2022–0599) and in compliance with the NIH Guide for Care and Use of Laboratory Animals guidelines.

## Supporting information



Supporting information

## Data Availability

The transcriptomic raw data have been deposited in the NCBI (https://www.ncbi.nlm.nih.gov/sra/PRJNA1111951) with an identifier of PRJNA1111951. Proteomic raw data have been deposited in the ProteomeXchange (https://www.iprox.cn//page/project.html?id = IPX0008823000) with an identifier of PXD052423.
