# Supplementary material for: Novel antibiotics against Staphylococcus aureus without detectable resistance by targeting proton motive force and FtsH
Source: MedComm (2020). 2025 Jan 8;6(1):e70046. doi: 10.1002/mco2.70046 (PMC11707430; doi:10.1002/mco2.70046)
Supplement: Supplementary file 1 — Supporting information [file MCO2-6-e70046-s001.docx]

Supplementary Materials for

**Novel antibiotics against *Staphylococcus aureus* without detectable resistance**

She Pengfei^1^, Yang Yifan^1^, Li Linhui^1^, Li Yimin^2^, Xiao Dan^2^, Guo Shaowei^2^, Huang Guanqing^2^, Wu Yong^2,*^

**Supplementary Results**

**The PDF file includes:**

Materials and Methods

Fig. S1. Structure-activity relationship of C218-0546 and its analogues.

Fig. S2. Bacterial killing dynamics of C218-0546 and STK848198 against *S. aureus.*

Fig. S3. Resistant mutation inducing by molecules.

Fig. S4. Bacterial killing dynamics of C218-0546/STK848198 against *S. epidermidis*, *E. faecium*, and *E. faecalis*.

Fig. S5. Low cytotoxicity of C218-0546 and STK848198.

Fig. S6. Antibiofilm activity of STK848198 against *S. aureus* and *S. epidermidis* determined by crystal violet staining.

Fig. S7. Viable cell counts of *S. aureus* ATCC 43300 after treated with 1/2×MIC of STK848198 for 1h.

Fig. S8. Biofilm inhibiting but no eradicating activity by C218-0546.

Fig. S9. Combinational antimicrobial effects between C218-0546 and conventional antibiotics.

Fig. S10. Pathological analysis of the abscess after treatment with AMK alone or in combination with C218-0546.

Fig. S11. One-step resistance inducing by AMP alone or in the presence of sub-MIC (0.5×MIC) of STK848198.

Fig. S12. Other potential targets of C218-0546/STK848198.

Fig. S13. Growth inhibition effects of C218-0546/STK848198 in the presence of varied pH values.

Fig. S14. Homology modeling of FtsH.

Fig. S15. FtsH synthesis and purification.

Fig. S16. Effects of STK848198 on ferric ion metabolism.

Fig. S17. Plasma protein binding ability and metabolism in liver microsomes.

Fig. S18. Collagen fibers observation by Masson staining.

Fig. S19. *In vivo* one-day toxicity by C218-0546 and STK848198.

Fig. S20. *In vivo* 7-day toxicity by C218-0546 and STK848198.

Fig. S21. Inflammation inhibitory effects of C218-0546 and STK848198.

Table S1. ADME/T prediction of C218-0546 and STK848198

Table S2. Antimicrobial activity of molecules against clinical isolates

Table S3. Spontaneous resistance frequencies of antimicrobials against *S. aureus* ATCC 43300

Table S4. MIC values of C218-0546/STK848198 against conditional pathogens

Table S5. Ames test of C218-0546

Table S6. Ames test of STK848198

Table S7. The pharmacokinetic parameters of C218-0546

Table S8. The pharmacokinetic parameters of STK848198

Table S9. Primers of biofilm-related genes used for qRT-PCR

Table S10. Primers of cytokines related genes used for qRT-PCR

Synthesis of C218-0546

Synthesis of STK848198

**Materials and Methods**

**Kirby-Bauer (K-B) test.** An overnight culture of *S. aureus* was adjusted to a McFarland (McF) standard equivalent of 0.5 and then spread onto a Mueller-Hinton (MH) agar plate using a sterile cotton swab. The blank discs loaded with compounds or equal volume of DMSO were placed on the agar surface and incubated at 37°C for 16-18 h. Then, the antimicrobial activity was determined by observing the diameter of the inhibition zones ^1^.

**Minimal inhibitory concentration (MIC) determination.** The detection of MIC was performed by the microbroth dilution method with reference to the previously report by Wang et al^2^. Briefly, the fresh bacterial suspension was adjusted to a McF standard equivalent of 0.5 (~1.5 × 10^8^ CFU/mL) and then 100-fold diluted with MH broth. Fifty microliters of bacterial suspension with equal volume of serially diluted compounds (1-128 µg/mL) were added into a 96-well cell plate (Corning Costar, the United States) and incubated at 37°C for 16-18 h. The bacterial suspension without compounds was used as a negative control. The lowest concentration that inhibited visible bacterial growth was considered as the value of MIC.

**Concentration-dependent growth inhibition.** *S. aureus* was cultured in TSB to log phase, and diluted with MH broth to ~2×10^6^ CFU/mL. One hundred microliters of the bacterial suspension with an equal volume of 2-fold diluted compounds were added in a 96-well cell culture plate. After incubation at 37 ℃ for 16h, the bacterial growth was quantified by measuring the OD value at 630nm (OD630nm).

**Resistance inducing assay.** Consecutive and one-step resistance experiments were performed to assess the resistance-inducing ability of the compounds against *S. aureus*. For consecutive resistance assay, the MICs of compounds and ciprofloxacin (CIP) were determined on the first day. After incubation at 37°C for 16-18h, the bacterial culture of 1/2× MIC was diluted by fresh MH broth at the ratio of 1: 1000. Subsequently, the equal volume of bacterial suspension was added to the serial concentrations of compounds or CIP into a 96-well plate, the new MICs were recorded every 24h for a total of 15 days. After 15 days of passages, the CIP-induced resistant *S. aureus* strains were obtained. And the antimicrobial activity of the compounds against the CIP-induced strains were assessed by microdilution assay as described above. For one-step resistance assay, overnight cultured *S. aureus* was adjusted to a final concentration of approximately OD630=0.5 with sterile saline. One hundred microliters of the bacterial suspension were uniformly spread in pre-prepared MH agar plates containing a specific concentration of compounds. Rifampicin (RFP) was used as a positive control. The viable cells were counted on the plates after incubation at 37°C for 48h. The frequency of spontaneous drug resistance mutation was defined as the ratio of the number of resistant CFU counts to the initial CFU counts^3^.

**Live/dead cells detection by SYTO9/ Propidium Iodide (PI) fluorescent probes.** Log phased *S. aureus* was adjusted to OD630 = 0.2 in 1× PBS in the presence of indicated concentrations of compounds. After incubation at 37 ℃ for 2h, the bacteria were collected in 1 mL 1×PBS by centrifugation at 3500×g for 10 min. The cells were then incubated with 10 μM of SYTO9 and PI mixture at the ratio of 1:1 (vol/vol) in the dark for 15 min. After washing the excess probes, the bacteria viability was visualized by a confocal laser microscope (CLSM, LSM800, ZESS, Germany)^4^.

**Wound infection model.** Six to seven-week-old ICR female mice were selected and a wound was built by cutting 1cm-diameter skin on the back. Then 50 μL of 1×10^8^ CFU/mL bacterial suspension were dropped on the wound of the mice. After 1h post infection, the indicated concentrations chemicals were spread on the infected area. After 24h treatment, the infected skin was removed, homogenized, and the number of viable bacteria in the tissue was counted. Meanwhile, the infected skins were cut for histopathological analysis by H&E staining ^5^.

**Biofilm determination by crystal violet staining.** The biofilm was formed as described above. The planktonic (non-adherent) bacteria were removed by washing the wells with PBS. Added a solution of 0.15% (wt/vol) crystal violet to each well and incubated for 15 min at room temperature. Then, the plates were gently wash with PBS to remove excess crystal violet. One hundred microliters of ethanol were added to each well to solubilize the crystal violet that has bound to the biofilm. The absorbance at 570nm (A570nm) of the solubilized crystal violet was measured with a microplate reader^6^.

**Biofilm determination by XTT assay.** To determine the biofilm inhibition, overnight cultures of *S. aureus* were diluted with TSB containing 2-fold diluted antimicrobials at the ratio of 1: 100 and added to a 96-well plate. After incubation statically at 37 ℃ for 24h, the supernatant was carefully removed and washed with PBS. (2,3-Bis-(2-methoxy-4-nitro-5-sulfophenyl)-2H-tetrazolium-5-carboxanilide) (XTT, 0.2 mg/ml) and Phenazin (methylsufate) (PMS, 0.02 mg/ml) were mixed with 1× PBS and added to each well of the 96-well plate (100 μL/well). The plate was then incubated for 3h in the dark, and the absorbance was measured at 490 nm (A490nm). For biofilm eradication, overnight cultures of *S. aureus* were diluted with fresh TSB at 1:100 and incubated for 24h to pre-form biofilms. Subsequently, the supernatant was removed and washed with PBS, and 200 μL of TSB in the presence of 2-fold diluted antimicrobials was added to each well. After incubation at 37 ℃ for 24h, the supernatant was removed and stained with XTT as described above^7^.

**Biofilm formation on titanium (Ti) discs.** To evaluate the anti-biofilm effects by C218-0546/STK848198 on medical implants, we attempted to establish biofilms on Ti discs. Briefly, for biofilm inhibition, overnight cultures of *S. aureus* were diluted 1: 100 with TSB in the presence or absence of serially diluted compounds. Three milliliters of the bacterial suspension were inoculated into a 6-well plate in the presence of sterile Ti discs in each well and incubated at 37°C for 24 h. The unattached cells were removed by PBS washing, and the remained biofilms were quantified by crystal violet staining and XTT staining, respectively, as described above. For biofilm eradication, the biofilms on Ti discs were pre-formed for 24h in the absence of any chemicals as described above, followed by the addition of serial concentrations of the compound. After further incubated at 37°C for 24h, the biofilms were quantified by violet staining and XTT staining as well^8^.

**Biofilm observation by confocal laser scanning microscopy (CLSM).** To further evaluate the biofilm inhibitory ability of the compounds, overnight cultures of *S. aureus* ATCC 43300 were diluted by TSB containing serial concentrations of compounds at the ratio of 1: 100. The bacterial suspension was added into 6-well plates (2 mL per well) fitted with sterile coverslips, and incubated statically at 37°C for 24h. Subsequently, the planktonic cells were carefully removed and the biofilm was washed with 1× PBS. Then, the biofilm was stained with SYTO9 and PI at a final concentration of 10 μM in dark for 15 min. The residual dye was washed by PBS and the biofilm was visualized by a CLSM (LSM800, Zeiss, Jena, Germany). For biofilm eradication, overnight cultured *S. aureus* ATCC 43300 was diluted with fresh TSB at the ratio of 1:100 and added to a 6-well plate in the presence of sterile coverslip in each well. After incubation at 37°C for 24 h, the planktonic cells were removed by PBS washing and the biofilm was continued incubated for another 24 h with indicated concentrations of STK848198. Subsequently, the biofilm was stained with SYTO9/PI as described above and observed by the CLSM.

**Initial adherence determination.** Overnight cultured *S. aureus* was diluted with fresh TSB to 0.5 McF. Two hundred microliters of the bacterial suspension in the presence of 1/4 or 1/2×MIC of STK848198 were added to a 96-well cell culture plate. After incubation at 37℃ for 1, 2 and 4h, respectively, the plate was washed with PBS and the OD630nm was recorded. Meanwhile, CLSM was used for the observation of initial adherence. Briefly, sterilized coverslides were added in a 6 well cell-culture plate, and 1 mL of the bacterial suspension combined with sub-MIC of STK848198 was added into each well. After incubation at 37 ℃ for an indicated time period (20 - 60 min), the coverslides were washed and stained with 10 μM of SYTO9 in the dark for 10 min. The fluorescence was acquired with the excitation and emission wavelength of 485 and 542 nm, respectively.

**Auto-aggregation.** Overnight grown *S. aureus* ATCC 43300 was diluted 1: 1 with fresh TSB in the presence or absence of indicated concentrations of STK848198. DMSO (1%, vol/vol) was used as a control. After treatment for 4 h at 37°C, the bacteria were washed twice with 1×PBS and resuspended to OD600=0.5. Then, the bacterial suspensions were statically incubated at 37°C for 2, 4, and 6h, respectively, the optical density at 630nm (OD630nm) was determined^9^.

**Extracellular polysaccharides (EPS) observation by Congo red agar.** The Congo red agar assay was performed to assess the EPS production in *S. aureus*. Firstly, the Congo red agar was prepared with 37 g/L BHI broth, 50 g/L sucrose, and 10 g/L agarose powder. The medium was autoclaved at 121 ℃ for 15 min. After cooling to 55 ℃, Congo red indicator solution (0.8 g/L) was added to the medium. Then, indicated concentrations of drugs were then added separately. The plate with DMSO was used as a negative control. Finally, the strains were incubated on the plates at 37°C for 24h. Black colonies indicated biofilms with plenty of EPS production, while red colonies indicated none ESP production^10^.

**EPS quantification by phenol-s****ulphoacid method.** The phenol-sulphoacid method was used to quantify the total EPS produced by *S. aureus*. Briefly, log phase *S. aureus* was treated with STK848198 at 37℃ 180 rpm for 12h, and DMSO treated *S. aureus* served as a control. The suspension was centrifuged at 8000 × g for 10 min at 4 ℃. The supernatant was mixed with 99% ethanol in a ratio of 1:2 and incubated overnight at 4°C. After centrifugation at 14000× g for 5 min at 4℃, the precipitate was suspended in distilled water. The cell suspension was vortex-mixed with 5% phenol and 95% sulphoacid and allowed to stand for 10 min at 30℃. Finally, the absorbance was measured at 490 nm (A490nm) and the total EPS production rate (%) was calculated as: (A490nm_treatment_/A490nm_ctrl._) × 100%^9^.

**Extracellular DNA (eDNA) and extracellular protein (eProtein) determination by spectrophotometry.** *S. aureus* biofilm was cultured in 6-well plates as described above for 24 h. The biofilm was gently washed twice with PBS to remove unattached cells. TSB medium containing sub-MIC concentrations of STK848198 was added to each well for 4h of treatment at 37 ℃. Then, the biofilm was collected in 5 mL of PBS and dispersed by Sonic Vibra Cell VCX processor. After centrifugation at 8000 rpm 4°C for 30 min, the supernatant was removed into a 2-fold volume of 95% ethanol and stored overnight at 4°C. The precipitate was collected at 10000 rpm 4°C for 2 min, dried at 60°C to remove residual alcohol and resuspended in 100 μL of PBS. The concentration of eDNA and eProtein were quantified by NanoDrop 2000 spectrophotometer (Thermo Fisher Scientific, Waltham, MA) at the absorbance of 260 nm (A260nm) and 280 nm (A280nm), respectively^11^.

**eDNA detection by agarose electrophoresis.** The eDNA was extracted as described above, followed by agarose gel electrophoresis. Briefly, 0.6 g of agarose was added into 30 mL of 1× Tris-acetate-EDTA buffer and melted in a microwave oven for 3-4 min. When the agar was completely dissolved, 3 µL of GoldenView (Transgene, Beijing, P.R. China). After the agarose gel has solidified, 5 µL of eDNA samples or DNA marker were added to the gel and further electrophoresed at 120 mA 25°C for 40 min. Finally, the eDNA fragments were visualized by a DNA visualizer^12^.

**eDNA observation by SYTOX Green staining.** Overnight cultured *S. aureus* was 1: 200 diluted with TSB. Two milliliters of the bacterial suspension in the presence or absence of 1/4×MIC of STK848198 were added to a 6-well cell culture plate. After incubation at 37℃ for 24h, the biofilms were washed, stirred and collected by centrifugation at 3500×g for 10 min. Then the supernatant was filtered through a 0.22 μm filter, 10 μM of SYTOX Green (Thermo Fisher, the United States) was used to stain the eDNA and further observed by the CLSM (ZEISS, Germany)^13^.

**Hydrophobicity detection.** The hydrophobicity assay was performed as previously described^9^. Briefly, overnight cultured *S. aureus* was incubated with TSB in the presence or absence of STK848198 for 3 h at 37 °C 180 rpm. After centrifugation, the precipitate was mixed with 2 mL of PBS to the optical density of 0.5 at 630 nm (OD_0_) and then vortexed with 0.5 mL of xylene for 2 min. After 15 minutes of static incubation, the optical density of the lower aqueous phase was measured at 630 nm (OD_t_). The formula was calculated as follows: Hydrophobicity (%) = (1- OD_t_/OD_0_) × 100%.

**RNA-seq.** Log-phased *S. aureus* ATCC 43300 was treated with 5×MIC of STK848198 at 37℃ 180 rpm for 1 h. The bacterial cells were collected centrifugation, and the total RNA was extracted using the TRIzol® reagent (Invitrogen, USA), with the addition of lysozyme to facilitate the release of the RNA. Then, the rRNA was removed with Ribo-Zero Magnetic kit (Illumina, USA) and the mRAN was fragmented to 200 bp size with fragmentation buffer. Next, the fragmented mRAN was further reverse transcribed, linked with adaptor with TruseqTM Stranded RNA sample prep Kit (Illumina, USA). The RNA-seq was performed with Truseq SBS kit (300 cycles, Illumina, USA) after digested with UNG enzyme (Illumina, USA). The raw data were collected and the differential expressed genes (DEGs) was further determined with R edgeR package software^14^. The raw data have been deposited in the NCBI (https://www.ncbi.nlm.nih.gov/sra/PRJNA1111951) with an identifier of PRJNA1111951.

**qRT-PCR for biofilm related genes quantification.** Log phased *S. aureus* ATCC 43300 was cultured in TSB with or without 5×MIC of STK848198 at 37 ℃ 180 rpm for 1h. Then the bacteria were harvested by centrifugation at 3000 × g for 15 min. The total RNA was extracted by using the E.Z.N.A. Bacterial RNA Kit (Omega, the United States) according to the manufacturer's instructions. The total RNA was reversely transcribed by using the TransScript All-in-One First-Strand cDNA Synthesis SuperMix (Transgene, Beijing, P.R. China). Quantitative PCR (qPCR) was used to quantify mRNA levels of *agrB*, *agrR*, *eno*, and *cna* relative to the control gene (16S rRNA) by using TransStart Tip Green qPCR SuperMix (Transgene, Beijing, P.R. China)^15^. The primers used were shown in Table S9.

**Red blood cell (RBC) lysis assay.** Human RBCs (Hemo Pharmaceutical and Biological Co.) were suspended in 1× PBS and diluted to a final concentration of 4% (vol/vol). The RBCs suspension was then incubated with serially diluted compounds at 37℃ for 1h. Then, the supernatant was collected by centrifugation and the absorbance at 570 nm (A570nm) was measured. Triton X-100 (0.1%, vol/vol) and DMSO (1%, vol/vol) were served as positive and negative controls, respectively^16^.

**Cytotoxicity assessment by Cell Counting Kit-8 (CCK-8).** The CCK-8 (DojinDo, Japan) was used to detect the cytotoxicity of compounds on human cell lines (LO2, HepG2, and HSF). Briefly, log phased cells were diluted with its optimal medium to ~4000 cells/well in a 96-well plate. After incubation at 37 ℃ with 5% CO2 for 24h, the unadhered cells were removed by PBS washing, and serially diluted compounds were added to each well for 24h incubation at 37 ℃ with 5% CO2. Then, 10 µL of CCK-8 was added to each well and the absorbance at 450 nm (A450nm) was recorded after 3 h of incubation^17^.

**Live/dead cell detection by Calcein-AM/PI dual staining.** Cell lines of LO2, HepG2 and HSF treated with indicated concentrations of C218-0546 or STK848198 for 24h were collected and resuspended in 1×PBS. Subsequently, Calcein-AM and PI were added to the cell suspension to the final concentration of 2 μmol/L and 8 μmol/L, respectively. Following a 30-min incubation at room temperature in the dark, the cells were observed by CLSM^18^.

**Apoptosis detection by cytometer.** Cell lines of LO2, HepG2, and HSF were cultured in RPMI medium or DMEM supplemented with 10% FBS and 1% (wt/vol) penicillin‐streptomycin. After incubation at 37℃ 5% CO_2_ to log phase, the cells were washed and resuspended in culture medium to a final density of 1×10^5^ cells per culture dish. The cells were fully adherent after cultured at 37℃ with 5% CO_2_ for 24h, washed and added with culture medium in the presence of 32 μg/mL of C218-0546 or STK848198. DMSO (1%) was used as a control. After incubation at 37℃ 5% CO_2_ for another 24h, the cells were digested with EDTA-free trypsin, collected, and washed twice with 1×PBS. The cells were harvested by centrifugation at 2000 rpm for 5 min, and resuspended in binding buffer in the presence of Annexin V-FITC and PI. Then, the suspension was incubated at room temperature for 10 min protected from light, and the apoptosis rate was detected by a cytometer (BD, the United States).

**hERG.** hERG channels expressed HEK293 cell line was cultured in DMEM at 37℃ with 10% FBS to ~70% of log phase. Then, the cells were washed with PBS and digested with trypsin. After incubation at 37 ℃ for 2-3 min, the cells were washed again and adjusted to ~ 2×10^3^/mL. The patch clamp detection experiment was performed after the cells attached to the cell wall. The extracellular fluid was prepared as 137 mM NaCl,1 mM MgCl_2_‧6H_2_O, 4 mM KCl,1.8 mM CaCl_2_‧2H_2_O, 10 mM D-Glucose, 10 mM HEPES, pH=7.4. And the intracellular fluid was prepared as 140 mM KCl,10 mM EGTA, 5 mM MgCl_2_‧6H_2_O, 10 mM HEPES, 5 mM Mg-ATP, pH=7.2. The current data was collected by using Pathmaster software. And the peak current inhibition rate was calculated as: $(1-\frac{Peak tail current compound}{Peak tail current vehicle})$. The concentration-effects were fitted by the Hill equation: *I*=*I*max∙(1/［1+（C_1/2_/［C］）^h^), where [C], C_1/2_, and h represented the drug concentration, half inhibitory concentration (IC_50_), Hill coefficient, respectively^19^.

**Drug combination assay.** The potential combinational antimicrobial effects between individual drugs were determined by checkerboard dilution assay. Briefly, overnight cultured *S. aureus* was diluted in MH broth to ~1× 10^6^ CFU/mL. Compounds were two-fold diluted by MH broth, then 50 μL of each compound were added to each well vertically and horizontally, respectively, in a 96-well cell culture plate. After incubation at 37 ℃ for 16-18h, the optical density at 630 nm (OD630nm) was recorded, and the MIC values used alone or in combination were determined. The fractional inhibitory concentration index (FICI) was calculated as (MIC_A in combination_/MIC_A alone_) + (MIC_B in combination_/MIC_B alone_)^20^.

**Bactericidal kinetics.** The mid-log phase S. aureus was diluted with MH broth to a concentration of 1 × 10^6^ CFU/mL. Then, the bacterial suspension was incubated with indicated concentrations of C218-0546 or STK848198 at 37°C and 180 rpm, and the viable cells was counted at the time point of 0, 2, 4, 8, 12, and 24 h, respectively^21^.

***S. aureus*-related abscess model.** Six to seven-week-old female, specific pathogen free outbred ICR mice were anesthetized with 1% sodium pentobarbital (50 mg/kg). The back hair was shaved with an electric razor. A 50 μL of *S. aureus* ATCC 43300 suspension containing 1× 10^8^ CFU/ml cells was subcutaneously injected (s.c.) into the back of the mice. The mice were randomized into four groups (N = 5-6 mice per group): (1) Vehicle group, (2) C218-0546 (30 mg/kg) or STK848198 (30 mg/kg) treatment group, (3) Amikacin (25 mg/kg) treatment group, (4) C218-0546 (30 mg/kg) or STK848198 (30 mg/kg) combined with amikacin (25 mg/kg) treatment group. Each group was administrated by s.c. after 1h post infection. The mice were sacrificed at 24h post infection, and their lesions were removed and homogenized for viable bacterial cells counting. Meanwhile, the lesions were dislocated and immersed in 4% paraformaldehyde for hematoxylin-eosin (H&E) staining, Giemsa staining and immunohistochemical analysis, respectively^22^.

**Scanning electron microscopy (SEM).** The bacterial strain of *S. aureus* was cultured in TSB medium at 37 °C 180 rpm to log phase. Then, the cultures were subsequently washed twice with sterile saline and diluted to a final concentration of 0.5 McF in fresh TSB medium containing 5 × MIC of C218-0546 or STK848198. The bacteria treated with 1% DMSO was served as a negative control. Following a one-hour incubation at 37 °C 180 rpm, the bacterial suspension was centrifuged at 4000 rpm for 8 min, and the resulting pellets were washed with PBS before being fixed in a 2.5% glutaraldehyde solution overnight. After washing twice with PBS, the samples were dehydrated through a graded ethanol series (30, 50, 70, 80, 90 and 100%). Finally, the samples were coated with gold-palladium for observation using SEM (Hitachi, Japan)^23^.

**Transmission electron microscopy (TEM).** The bacterial suspension was prepared as described above, and the suspension was subjected to centrifugation at 4000 rpm for 8 min, after which the supernatants were decanted. The specimens were then fixed in a solution consisting of 0.2 M sodium cacodylate buffer, 2.5% paraformaldehyde, 5% glutaraldehyde, and 0.06% picric acid. The fixed cells were first washed with 0.1 M cacodylate buffer and subsequently postfixed with a solution containing 1% osmium tetroxide and 1.5% potassium ferrocyanide for a duration of 60 min. Following this, the samples underwent three washes with maleate buffer and were then exposed to 1% uranyl acetate in maleate buffer for an additional 60 min. The bacterial cells were then dehydrated using a series of ethyl alcohol concentrations (30%, 50%, 70%, 90%, and 100%). After a 1-hour incubation in propylene oxide, the samples were infiltrated with a 1:1 (vol/vol) mixture of propylene oxide and Epon for another hour. Finally, the specimens were polymerized at a temperature of 60 °C for a period of 48 h. Ultrathin sections (60 nm) cut using an ultramicrotome (Reichert Ultracut-S) and stained with lead citrate. Finally, the cells were observed using TEM (Hitachi, Japan)^24^.

**Proton motive force (PMF) determination by BECEF-AM probe.** The pH-sensitive fluorescent probe BCECF-AM was employed to measure the PMF of *S. aureus*. Briefly, *S. aureus* ATCC 43300 were grown to OD630nm = 0.5 in TSB. Then, the bacterial cells were centrifuged, washed with PBS, and resuspended in 5 mL of HEPES buffer (pH=7.2) in the presence of indicated concentrations of C218-0546 or STK848198. And BCECF-AM probe was added to the suspension to the final concentration of 20 mM. Glucose (25 mM) was used as a control. After incubation at 37 ℃ for 30 min, the fluorescence was monitored by using a fluorescence spectrometer at excitation and emission wavelengths of 500 nm and 522 nm, respectively^25^.

**PMF determination by DiSC3(5) fluorescent probe.** Exponential phased *S. aureus* cells were subjected to three washes with PBS and subsequently adjusted to an optical density of 0.05 at 630 nm in HEPES buffer containing 5 mM glucose and 100 mM KCl at a pH of 7.2. Next, the bacterial suspension was incubated with 2 μM DiSC3(5) for 30 min. A mixture of 90 µL of the bacterial suspension and 10 µL of C218-0546 or STK848198 at specified concentrations was dispensed into a 96-well black-walled plate. Melittin (16 μg/mL) was served as a positive control in this experiment. Fluorescence intensity was recorded with the excitation and emission wavelengths of 622 nm and 670 nm, respectively^26^.

**Intracellular ATP quantification.** The *S. aureus* ATCC 43300 was overnight cultured in TSB, and centrifuged at 3000 × g for 15 min to collect the precipitate. The bacterial cells were washed for 3 times with 1×PBS (pH 7.4) and resuspended to OD630 = 0.5. Then, C218-0546 or STK848198 was added to the bacterial suspension to achieve the final concentrations of 1 - 32 μg/mL. After incubation at 37 ℃ for 1h, the bacterial suspension was centrifuged at 4 ℃ 3000 × g for 15 min to obtain precipitation. Subsequently, the intracellular ATP was ultrasonically extracted, and the supernatant was harvested. The detection reagent was mixed with the supernatant at the ratio of 1: 9, and the luminescence was measured by a fluorescence spectrometer^27^.

**ATP quantification by ATP-Red1 probe.** The intracellular ATP observation and quantification were performed by ATP-Red1 fluorescent probe. Briefly, log-phased *S. aureus* was treated or untreated with C218-0546 or STK848198 for 30 min, harvest by centrifugation at 3500 × g for 15 min. After washed with 1×PBS for 3 times, the bacteria were resuspended in PBS in the presence of 5 μM of ATP-Red1 probe. After incubation at room temperature for 15 min in the dark, the fluorescence intensity was monitored by a microplate reader with the excitation and emission wavelength of 570 nm and 590 nm, respectively^28^.

**ATPase activity determination.** ATPase activity detection was performed using an ATPase kit (BB-4719, Besbio, Shanghai, P.R. China). Briefly, log phased bacteria were adjusted to OD630 = 0.5 in 1× PBS in the presence of indicated concentrations of compounds. After incubating at 37 ℃ for 1h, the bacteria were washed and resuspended in 1-2 mL PBS. Then, cell ultrasonic crusher was used to fully crush the bacteria cell wall to release all ATPase in the bacteria. Refer to the instruction manual of the kit for subsequent operations. The optical density at 630 nm (OD630nm) was recorded and the ATPase concentration was calculated.

**Molecular dynamics (MD) and molecular docking.** Homology modeling was performed by Modeller v9.19 software based on the three-dimensional (3-D) structure and the sequence alignment of FtsH. Structural optimization of FtsH was carried out by using Amber14SB force field with a 2000-step steepest descent method as well as a 2000-step conjugate gradient method, respectively. The model was evaluated by PROCHECK program.

The structure of C218-0546 and STK848198 was optimized by MOPAC program. Autodock 4.2.6 And Autodock Tools 1.5.6 software were used for molecular docking assay. The docked boxes were set to wrap around the entire protein with the center point coordinates of -2.20, 99.75, and 38.22. The number of lattice points in XYZ direction was set to 60×60×60 with a space of 0.375 Å. The number of molecular docking times was set to 100, and other parameters were set to default values. Dock C218-0546 and STK848198 to the active site of FtsH protein, respectively.

The MD stimulation was performed by Gromacs 2019.6 software. The protein and water molecules were analysis by Amber14SB all atomic force field TIP3P model. During the simulation, all hydrogen bonds were constrained using the LINCS algorithm, with an integration step of 2 fs. The electrostatic interaction was calculated using the Particle-mesh Ewald method. The cutoff value for non-bonding interactions was set to 10 Å and updated every 10 steps. The stimulation temperature was set to 300 K with V-rescale method, and the Parrinello-Rahman method was used to control the pressure to 1 bar. Firstly, the steepest descent method was used to minimize the energy of the three systems (FtsH, FtsH-C218-0546, and FtsH-STK848198). Then, perform 100 ps NVT equilibrium at 300 K. Finally, 100 ns MD simulations were performed on each of the three systems. The simulation results were visualized using Gromacs program and VMD^29-34^.

**Surface plasmon resonance (SPR).** To detect the affinity between the small molecular compounds and FtsH, SPR analyses were performed on a multiple flow cell Biacore T200 instrument (Cytiva, the United States). Briefly, FtsH (35.5 µg/mL in 10 mM sodium acetate buffer, pH= 4.0) was covalently immobilized on a dextran matrix CM5 sensor chip surface in three different flow cells. The excessive active groups on the dextran matrix were blocked using 1 M ethanolamine at the velocity of 10 μL/min for 15 min. The uncoated reference flow cell on the fourth flow cell was prepared on the dextran matrix treated as described above but without any ligand. 1×PBS (pH 7.4) containing 2 mM KH2PO4, 137 mM NaCl, 10 mM Na2HPO4, 2.7 mM KCl and 5%DMSO was used as the running buffer. In the running buffer, a 2-fold serially diluted of compounds was passed over the ligand at a flow rate of 30 µL/min. The sensorgrams were recorded at 25 °C. The response units at the steady state were fitted to the Langmuir equation to yield the Kd values^35^.

**Proteomics.** *S. aureus* strains were cultivated to a mid-log phase and adjusted to an OD630 of 0.2. The bacterial suspension was treated with 5 × MIC of C218-0546 or STK848198 for 1 h. Meanwhile, the bacteria treated with DMSO were set as control. Then, the bacterial sediment was collected at 4℃ and washed with 1×PBS and quick-frozen with liquid nitrogen. The bacterial was stored at −80°C. Proteins extraction was performed by lysis with SDT buffer containing 4% SDS, 1 mM DTT, and 100 mM Tris-HCl (pH=7.6). The proteins were digested with trypsin to peptides quantified by BCA Protein Assay Kit (Bio-Rad, USA). Then, peptides desalt was performed with a C18 Extraction Disk (3 M Empore), enriched by vacuum centrifugation, and reconstituted in the presence of formic acid. TMT mass spectrometry analysis was performed on Thermo Q Exactivetm HF-x. The differentially expressed proteins (DEPs) were considered as a |fold change|≥1.5 with *P* < 0.05 ^2^. All the raw data have been deposited in the ProteomeXchange (https://www.iprox.cn//page/project.html?id=IPX0008823000) with an identifier of PXD052423.

**Efflux pump inhibition assay.** The mid-log phase *S. aureus* strains were adjusted to an OD630 of 0.3 in 1× PBS, containing 0.6% (vol/vol) glucose with 2 μg/mL of ethidium bromide (EtBr). After incubation for 5 min in the dark, the labelled bacterial suspension was treated with 1/4-2×MIC of C218-0546 or STK848198 in a 96-well cell culture plate. 1/2×MIC of CCCP (1 μg/mL) was used as a positive control. The fluorescence intensity was measured every 5 min for a period of 1 h with the excitation and emission wavelengths of 485/530 nm and 525/585 nm, respectively^36^.

**Pharmacokinetics determination.** Six to seven-week-old female ICR mice were administrated by s.c., intravenously (i.v.), intraperitoneally (i.p.) or orally (p.o.) with C218-0546 or STK848198 at the dosage of 30 mg/kg. Blood samples were extracted from the jugular vein at intervals of 0.083, 0.25, 0.5, 1, 2, 4, 8, and 24h post-treatment, respectively. The plasma was separated following centrifugation at 6800 × g for 6 min. Concentrations of these compounds in the plasma were quantified by using the LC-MS/MS-04 instrument (TQ4000, JEOL, Japan), and pharmacokinetic parameters were calculated by using Phoenix WinNonlin 7.0 software (Certara, Princeton, NJ, USA)^37^.

**Peritonitis-sepsis model.** Six to seven-week-old female ICR mice were included. Log phased *S. aureus* was washed with saine and resuspended in the saline to a turbidity of 2.5 and 3.5 McF for C218-0546 and STK848198 respectively (higher concentration of bacterial cells for STK848198 treatment was prepared due to the stronger antimicrobial activity by STK848198 than C218-0546, which could be better for its synergy observation). Mice were injected with 500 μL of the suspension in the presence of 5% mucin by i.p.. An hour post-infection, the mice were administered with: 1) EL+ethanol, 2) C218-0546 (30 mg/kg), and 3) STK848198 (30 mg/kg) alone or in combination with ampicillin (AMP, 25 mg/kg)^38^ or amikacin (AMK, 25 mg/kg). The mice were euthanized with CO2 inhalation at 24h post infection, and organs of liver, spleen, and kidney were collected for CFU counting^39^.

**Periprosthetic joint infection (PJI) model.** PJI model was performed as previously reported by Jiang et al.^13^ with minor modifications. Briefly, 6-week-old female ICR mice were randomly divided into 4 groups: 1) Sham group without infection or drug treatment. 2) Vehicle group (infected mice treated with EL+Ethanol). 3) Infected mice treated with 30 mg/kg STK848198. 4) Infected mice treated with 25 mg/kg AMP. 5) Infected mice treated with 30 mg/kg STK848198 + 25 mg/kg AMP. *S. aureus* ATCC 43300 was overnight cultured at 37 ℃ 180 rpm, washed twice with PBS and resuspended in saline. Mice were anesthetized with 1% pentobarbital sodium. Their knees were disinfected with 75% ethanol, and then dissected, exposed the femur. A 0.3 mm×0.8 mm needle (Sinocare, Changsha, P.R. China) was inserted into the femur as an implant. The skin was sutured and injected with 1×10^6^ CFU *S. aureus*. Then, the mice were administrated with the reagents by i.p. injection as described above in each group. At 3, 5, and 7 days post infection, the bacterial burden of joint bone tissue and surrounding soft tissue were counted by CFU counting. Meanwhile, at 14 days after infection, the knees with implants were collected for X-ray and micro-CT scanning (Bruker SkyScan1276, Bruker Micro-CT, Germany) analysis. The parameters of bone mineral density (BMD), bone volume/total volume (BV/TV), trabecular thickness (TB.TH), trabecular number (TB.N), and trabecular Separation/Spacing (TB.SP) of each group were analyzed by the CTAn (version 1.18.4.0) program (Skyscan Company, Bruker Micro-CT, Germany). In addition, the femurs of the mice were harvested, fixed with 4% paraformaldehyde, and performed with pathological examination.

**Absorption, distribution, metabolism, excretion/toxicity (ADME/T) prediction.** The ADME/T assessment was performed by using StarDrop V7.3, ADME/TLab v2.0 software (<http://admetmesh.scbdd.com>) and ADME/TSAR v2.0 (<http://lmmd.ecust.edu.cn/admetsar2>). The absorption, distribution, metabolism and excretion of the compounds in humans was evaluated by the Prediction Deck tool^40^.

**Effects of proton concentrations on the antimicrobial activity of C218-0546/STK848198.** The mid-log phase *S. aureus* strains were diluted to the indicated concentrations of C218-0546 or STK848198 in varied pH-adjusted MH broth. Subsequently, the bacteria were incubated at 37°C for 16 h, and the growth turbidity of the bacteria was quantified by measuring the OD630nm^41^.

**Protein synthesis and purification.** The primers in this study were designed according to the sequence of *S. aureus* ATCC 43300 genome published on NCBI. The full-length fragment of FtsH gene was amplified by PCR and was inserted into the pET28a plasmid and transferred into *E. coli* BL21 to obtain the recombinant expression strain BL21-PET28a-FtsH after BamH I and XhoI double restriction digestion. The strain of BL21-PET28a-FtsH was cultured in LB broth at 37℃ 250 rpm to OD60=0.6, then incubated with 0.5 mM IPTG at 18 ℃ for 16h. The bacteria were collected by centrifugation at 4℃, resuspended in lysis buffer (50 mM Tris, 0.5 M NaCl, pH=8.0) and lysed by ultrasonic crack (work 3s, off 2s, 15 min, twice). After centrifugation at 4℃, the precipitate supernatant was collected and dissolved in denatured buffer (50 mM Tris, 0.15 M NaCl, 8 M urea, pH=8.0). The supernatant was filtered by a 0.45 μm filter membrane, and purified with Ni-NTA Agarose affinity chromatography column. Finally, the target protein was detected by SDS-PAGE. The FtsH protein was stored at -80℃.

**Competitive growth inhibition by addition of peptidoglycan (PGN).** Log-phased *S. aureus* was diluted with fresh TSB in the presence of 1-2×MIC of C218-0546 or STK848198 with or without 40 μg/mL of PGN. DMSO was used as a control. The bacterial suspension was incubated at 37 ℃ 180 rpm at indicated time, and the growth turbidity was determined by measuring the OD values at 630 nm. Meanwhile, 5 μL of the bacterial suspension was dropped onto the sheep blood agar at the time point of 12h. After incubation at 37 ℃overnight, the colonies on the agar were recorded.

**SYTOX Green staining.** Log-phased *S. aureus* ATCC 43300 was collected by centrifuged at 4000× g for 15 min, washed three times with 1×PBS (pH = 7.4) and resuspended to OD630= 0.5. Then STYOX Green was added to the bacterial suspension to a final concentration of 2 μM. After incubation in the dark for 15 min, the mixture was added to a black 96-well plate in the presence of serially diluted compounds. DMSO (1%) and 16 μg/mL of melittin were prepared as positive and negative control, respectively. The fluorescence intensity was measured every 5 min for a total of 30 min by a microplate reader at the excitation and emission wavelengths of 485 and 525 nm, respectively^2^.

**Intracellular ROS quantification.** The level of intracellular ROS after treated with C218-0546/STK848198 was determined by a 2’,7-dichlorofluorescein diacetate (DCFH-DA, Beyotime, Shanghai, P.R. China) probe. Briefly, *S. aureus* ATCC 43300 was grown to log phase in TSB. The cells were washed for 3 times with 1×PBS (pH = 7.4) and resuspended in the PBS to OD630 = 0.5. Then, the DCFH-DA probe was added to the bacterial suspension to a final concentration of 10 μM. After incubation for 30 min, the suspension was washed twice with PBS, 90 μL of the probe-labeled cells with 10 μL of the tested compounds were added into a 96-well plate. After incubation at 37°C for 30 min, the fluorescence intensity was measured by a microplate reader with the excitation and emission wavelengths of 488 nm and 525 nm, respectively^42^.

**Plasma protein binding rate calculation.** Human plasma was purchased from IPHASE (Beijing, China). C218-0546 or STK848198 was added into human plasma to a final concentration of 0.5, 2 and 5 μg/mL, respectively. Warfarin sodium and PBS was used as positive control and blank control, respectively. The samples were added to the upper side of the dialysis membrane (IPHASE, Beijing, China) and PBS was added to its lower side. After incubation at room temperature for 12h, the concentration of C218-0546 and STK848198 at the both sides were detected by the LC-MS. The plasma protein binding rate (%) was calculated as: (1-Cf/Cp) × 100%. Cf, concentration of free compounds (lower side). Cp, plasma protein binding compounds (upper side).

**Liver microsomal metabolism analysis.** Human liver microsome (20 mg protein/mL) was purchased from IPHASE (Beijing, China). One microliter of C218-0546 or STK848198 (200 μM) was incubated with 2 μL of liver microsome in the presence or absence of NADPH. Phenacetin (100 μM) was used as a positive control. After incubation at 37℃ for 0, 15, 30, 45, 60 and 90 min, respectively, the samples were cooled on ice and added with 500 μL of stop solution. The remained compounds were quantified with a LC-MS (Triple Quad™ 5500, AB SCIEX, USA).

**Ames test.** Genotoxicity of the compounds was determined by Ames Kit (IPHASE, Beijing, China) containing *Salmonella typhimurium* (TA97a, TA98, TA100, and TA1535) and *E. coli* WP2 *uvrA* (pKM101). Diclofenac and 2-aminofluorene were used as positive controls for TA97a and TA98. Methyl methanesulfonate and 2-aminofluorene were used as positive controls for TA100. Methyl methanesulfonate and 2-aminoanthracene were used as positive controls for WP2 *uvrA* (pKM101). NaN3 and 2-aminoanthracene were used as positive controls for TA1535. The bottom and top layer culture medium, and S9 mixed solution were prepared according to the manufacturer’s instructions. After incubation at 37℃ for 48h, the colonies were counted.

**Masson staining.** Specific 6-8-week old female ICR mice were subcutaneously administrated with 30 mg/kg of C218-0546 or STK848198, respectively. Meanwhile, EL+ethanol was used as a control. After 24h post administration, the mice were euthanized, and the skin located on the treated area was scissored, and immersed in 4% paraformaldehyde. Then, Masson staining was performed to assess the dermal toxicity of the compounds.

***In vivo* toxicity analysis.** For acute *in vivo* toxicity determination, specific ICR female mice (N = 6 per group) were randomly divided into 3 groups. The mice were intraperitoneally injected with EL+ ethanol (vehicle group), or 30 mg/kg of C218=0546 or STK848198. Twenty-four hours post-administration, blood samples were collected from the ophthalmic venous plexus and the hematological parameters of white blood cell count (WBC), neutrophil ratio (N%), red blood cell count (RBC), hemoglobin levels (HGH), and platelet count (PLT) were measured. Meanwhile, serum samples were also collected by centrifuged at 4000 rpm for 10 min. And the biomarkers of alanine aminotransferase (ALT), urea nitrogen (BUN), and creatine kinase (CK) for liver, kidney and heart function monitoring, respectively, were also analyzed. Then, the mice were euthanized, and the organs of heart, liver, spleen, lungs and kidneys were removed and fixed in 4% neutral paraformaldehyde for Hematoxylin and eosin (H&E) staining. In addition, for 7-day *in vivo* toxicity detection, specific ICR female mice (N=5 per group) were intraperitoneally injected with 30 mg/kg of C218=0546 or STK848198 every day for a total of 7 days. The body weight was monitored, and the blood samples and organs were also collected and analyzed as described above^43^.

**Inflammatory factors inhibition by compounds.** Cell line of RAW264.7 was cultured in DMEM medium with 10% FBS. After incubation at 37 ℃ 5% CO2 to log phase, the attached cells were washed with 1× PBS and added with 1 μg/mL of LPS. After incubation at 37 ℃ 5% CO2 for 30 min, 16 μg/mL of C218-0546 or STK848198 was added to each well. The cells were further cultured for 12h, and the cell precipitate was collected for qRT-PCR while the supernatant was collected for ELISA.

For gene expression determination. One milliliter of Trizol was added to lysis the cells. Then, the suspension was incubated with 200 μL chloroform for 3 min. After centrifugation at 12000 rpm 4℃ for 15 min, the aqueous phase was removed to RNase-free tubes, and added with equal volume of isopropanol. After centrifugation at 12000 rpm 4℃, the RNA precipitation was collected and wash with 75% ethanol. The RNA was air dried for 5-10 min, and dissolved in RNase-free water. Absorbance at 260nm and 280 nm was detected to determine the RNA concentration and purity. cDNA was synthesized with HiFiScript cDNA Synthesis Kit (CoWin Biosciences, Beijing, China) according to the manufacture’s specification, and UltraSYBR Mixture (2×, CoWin Biosciences, Beijing, China) was used for qPCR determination. The sequences of the primers were shown in Table S10.

ELISA kit (Proteintech, Wuhan, China) was used for cytokines production quantification. Briefly, 100 μL of the cell supernatant or the standardized solution was added to antibody coated 96-well cell culture plate. After incubation at 37 ℃ for 2h, the plate was washed with PBS, and added with 100 μL of antibody working solution, sealed and incubated at 37℃ for 1h. The plate was washed again with PBS, and added with 100 μL of HPR-labeled avidin for 40 min incubation at 37 ℃. Then, 100 μL substrate was added to each well and the absorbance at 450 nm was determined.

**Supplementary Figures:**


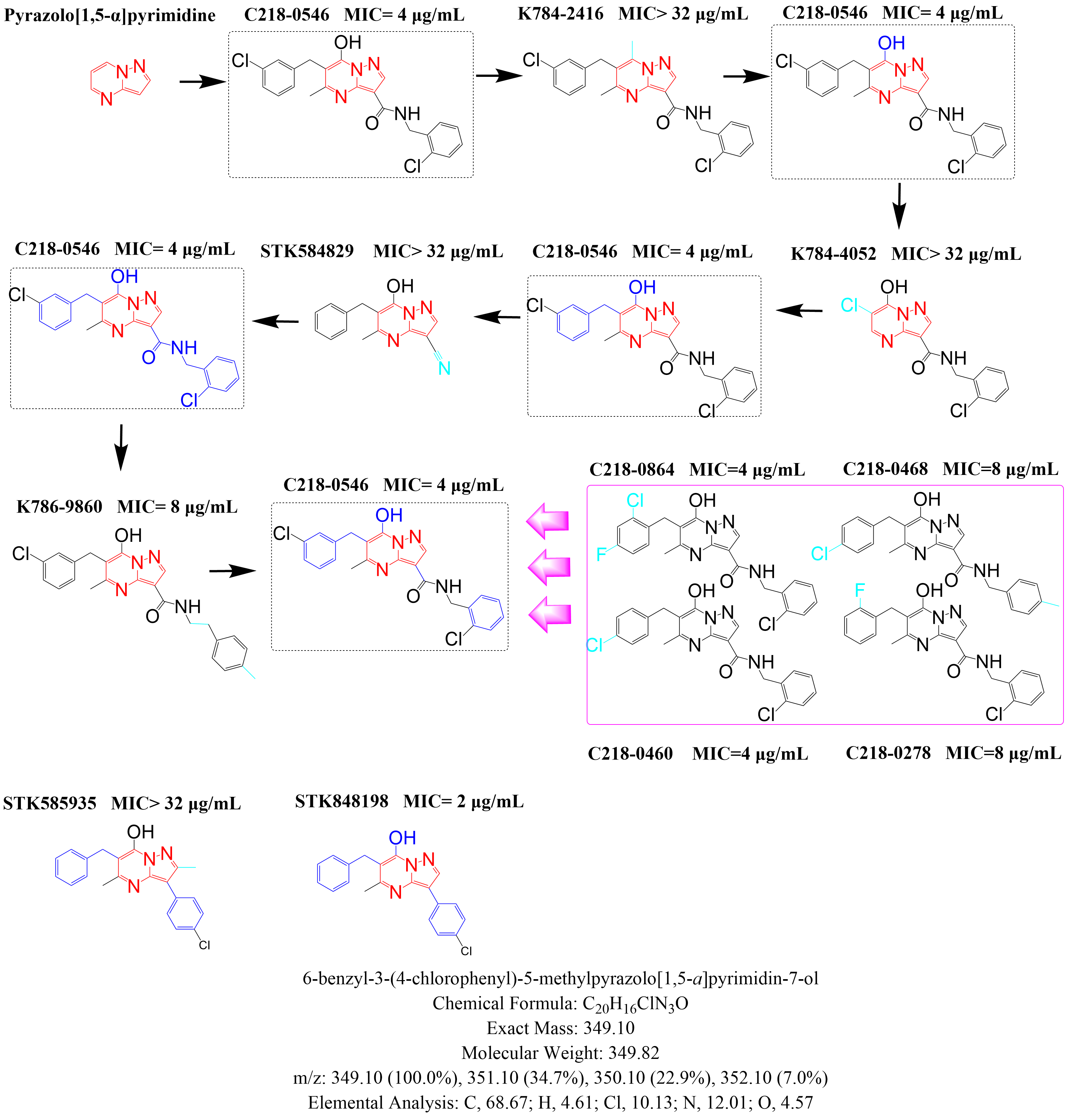
**Supplementary Figure 1. Structure-activity relationship of C218-0546 and its analogues.** Red sticks indicated the scaffold of C218-0546 and its analogues. Blue sticks indicated the indispensable groups for the antimicrobial activity of C218-0546. Bright green sticks indicated the substitutional or additional groups for the exploring of functional groups on C218-0546.


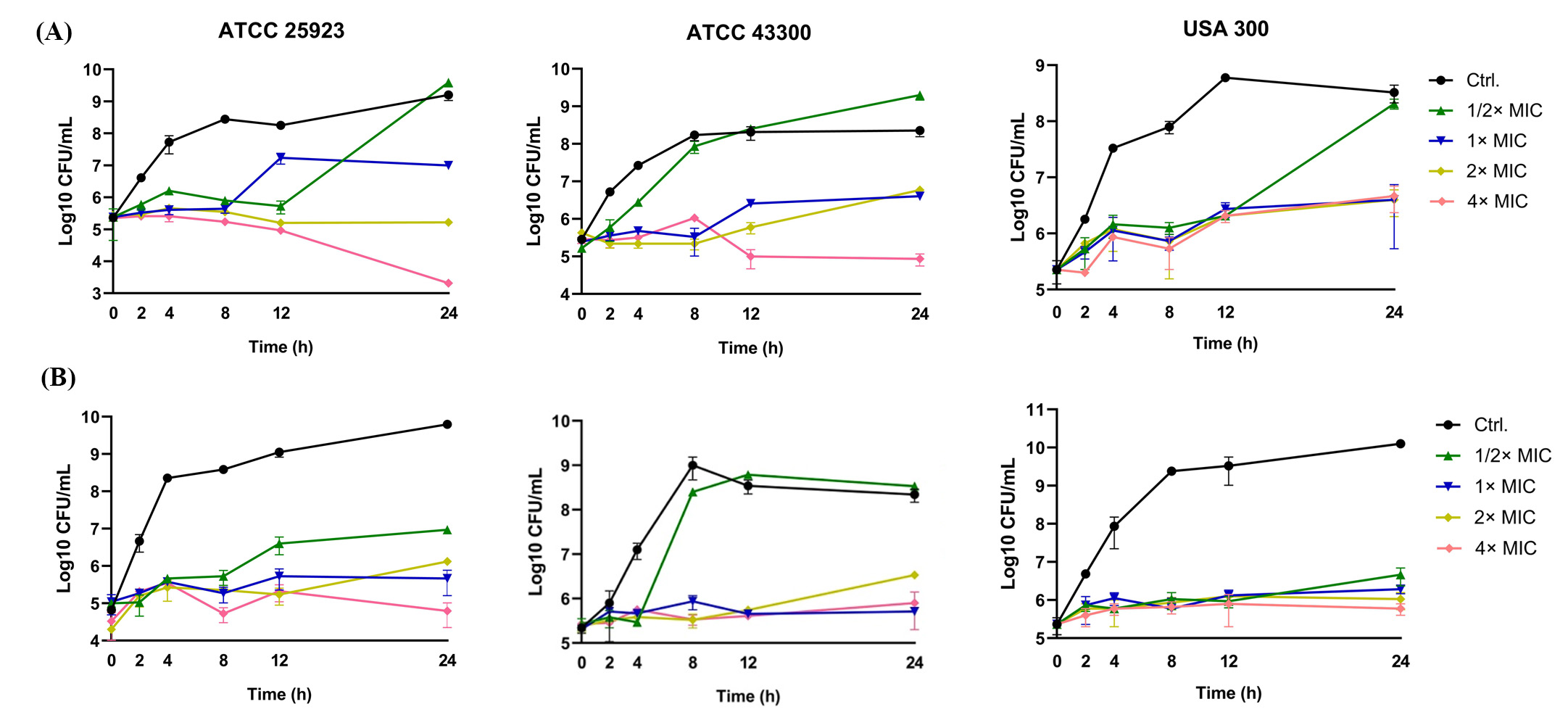
**Supplementary Figure 2.** Bacterial killing dynamics of C218-0546 (A) and STK848198 (B) against *S. aureus* type strains MSSA ATCC 25923, MRSA ATCC 43300 and MRSA USA300.


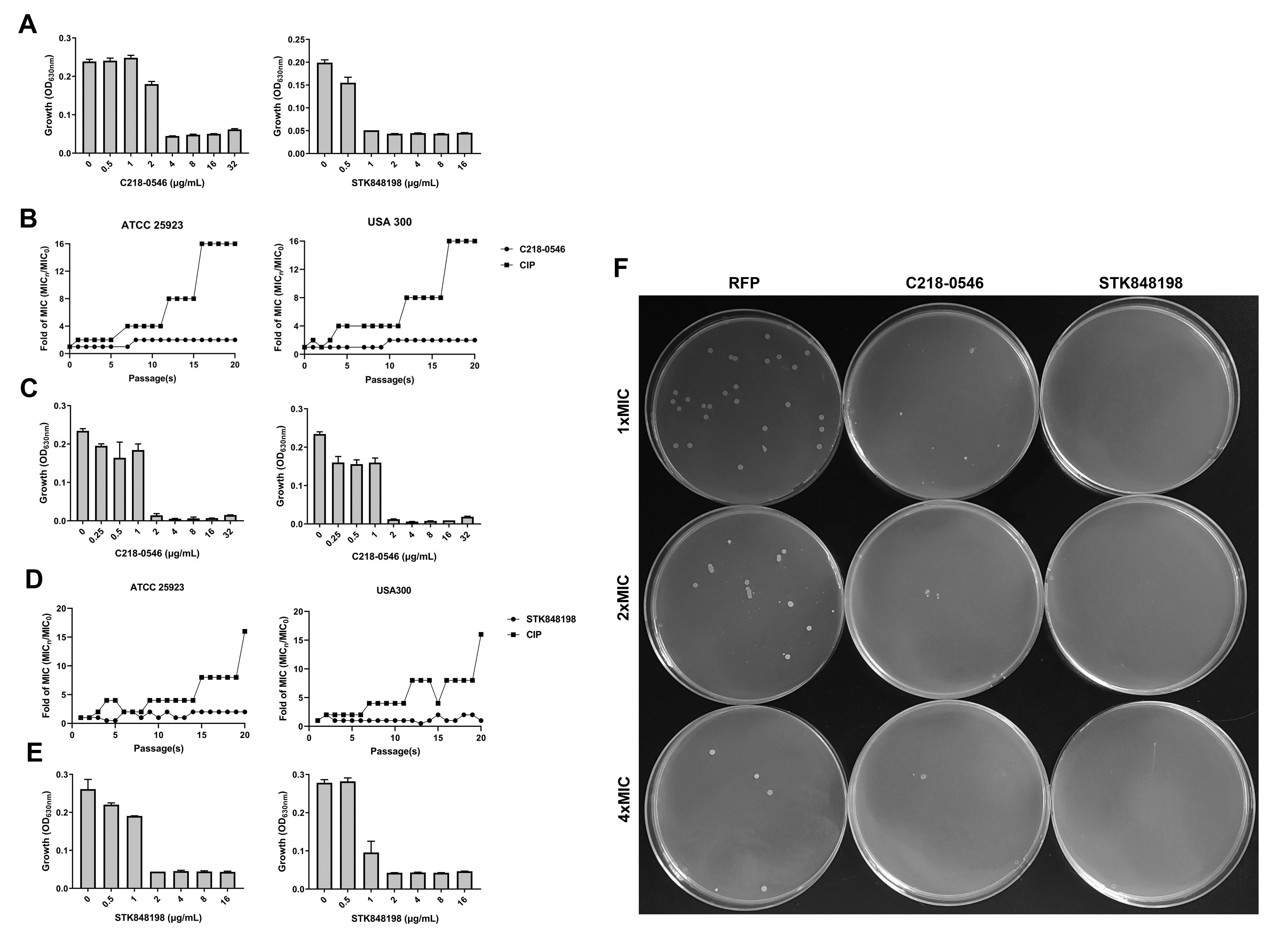
**Supplementary Figure 3. Resistant mutation inducing by molecules.** (A) Antimicrobial effects of C218-0546/STK848198 against CIP-induced *S. aureus* ATCC 43300. The CIP-induced *S. aureus* was induced by CIP for 20 passages, and its MIC by CIP was 32-fold higher than the initial generation. (B) Resistance mutation detection of ATCC 25923 and USA300 by consecutive sub-MIC of CIP or C218-0546 inducing. (C) Antimicrobial susceptibility of C218-0546 against CIP-induced strains at the 20^th^ passage. (D) Resistance mutation detection of ATCC 25923 and USA300 by consecutive sub-MIC of CIP or STK848198 inducing. (E) Antimicrobial susceptibility of STK848198 against CIP-induced strains at the 20^th^ passage. (F) Representative images of the resistance occurrence by one-step induction. RFP was used as a control.


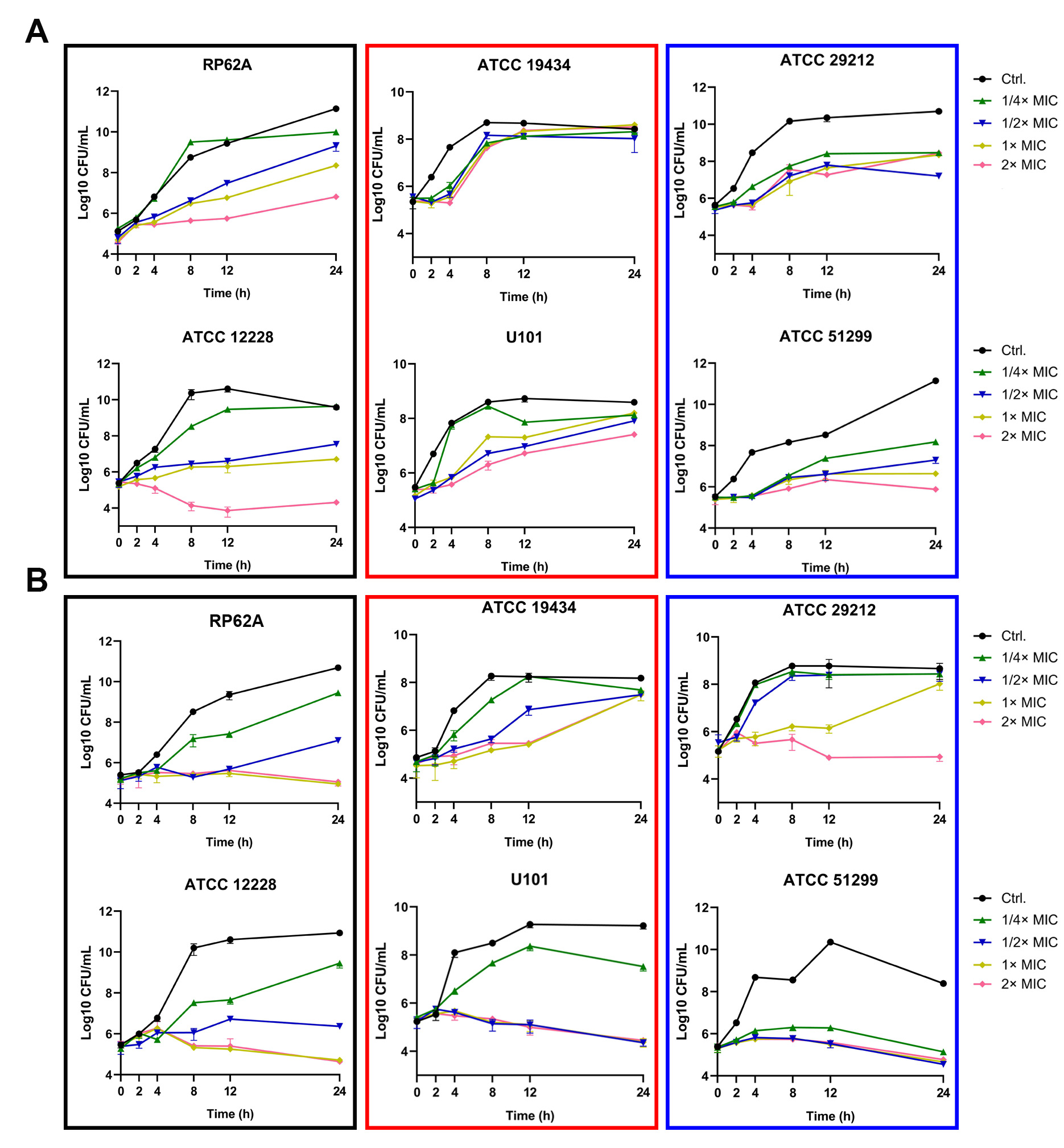


**Supplementary Figure 4**. Bacterial killing dynamics of C218-0546 (A) and STK848198 (B) against *S. epidermidis* (RP62A and ATCC 12228), *E. faecium* (ATCC 19434 and U101), and *E. faecalis* (ATCC 29212 and ATCC 51299), respectively.


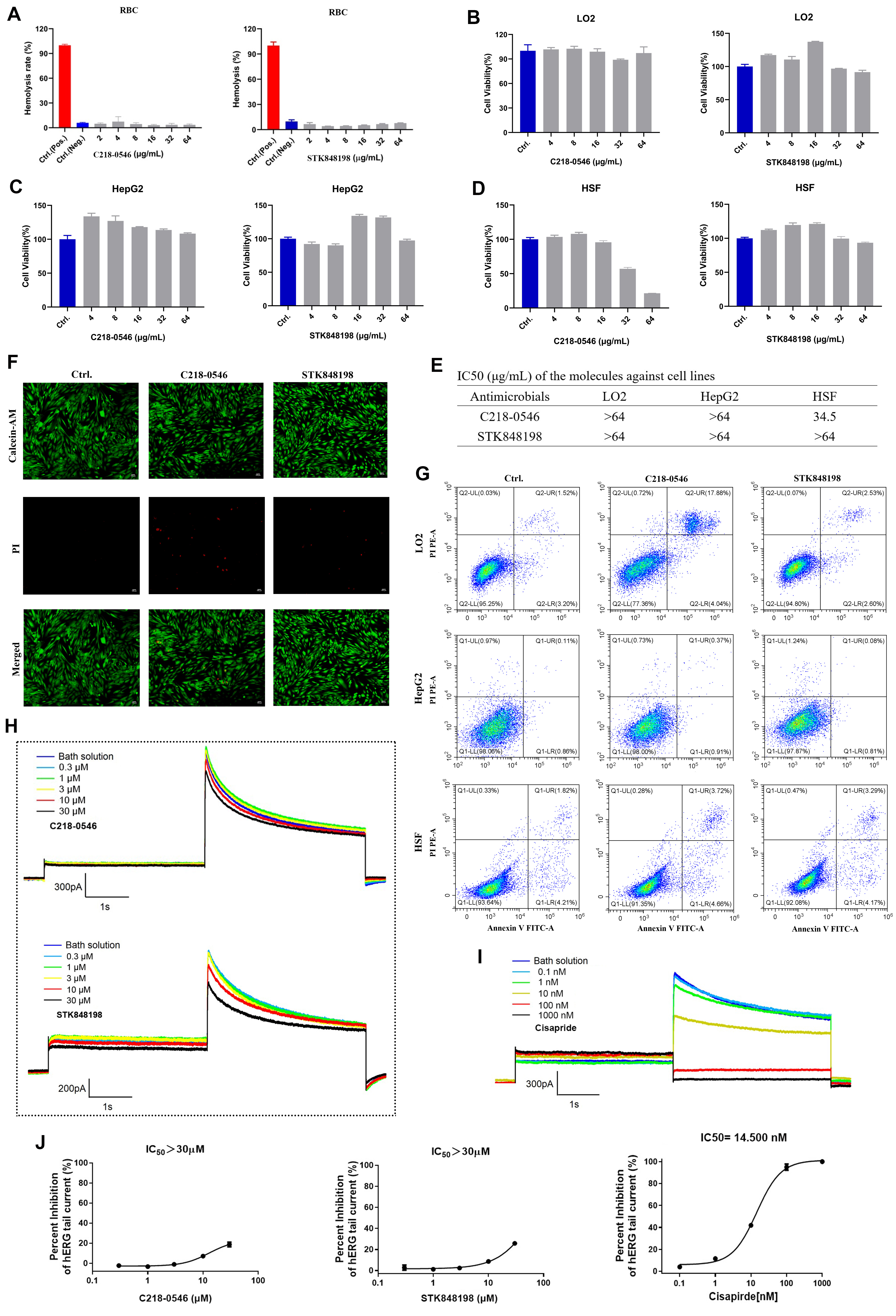


**Supplementary Figure 5. Low cytotoxicity of C218-0546 and STK848198.** (A) No RBC lysis activity was detected by C218-0546/STK848198 treatment. (B, C) No detectable cytotoxicity to the cell lines of LO2 (B) and HepG2 (C) was observed after treatment with the compounds. (D) Moderate cytotoxicity of C218-0546 against HSF at the concentration up to 32 μg/mL, but no cytotoxicity observed by STK848198. (E) IC_50_ of the compounds to the tested cell lines. (F) No dead HSF cells was observed after treated with 32 μg/mL of the compounds for 24h by Calcein-AM/PI staining. (G) Cell apoptosis assessment by Annexin V/PI staining. The cell lines of LO2, HepG2, and HSF were treated with 32 μg/mL of C218-0546/STK848198 for 24h. (H) No detectable cytotoxicity of C218-0546/STK848198 to potassium channels by hERG assay. (I) Concentration-dependent myocardial cytotoxicity of cisapride. (J) IC_50_ calculation of the hERG assay.


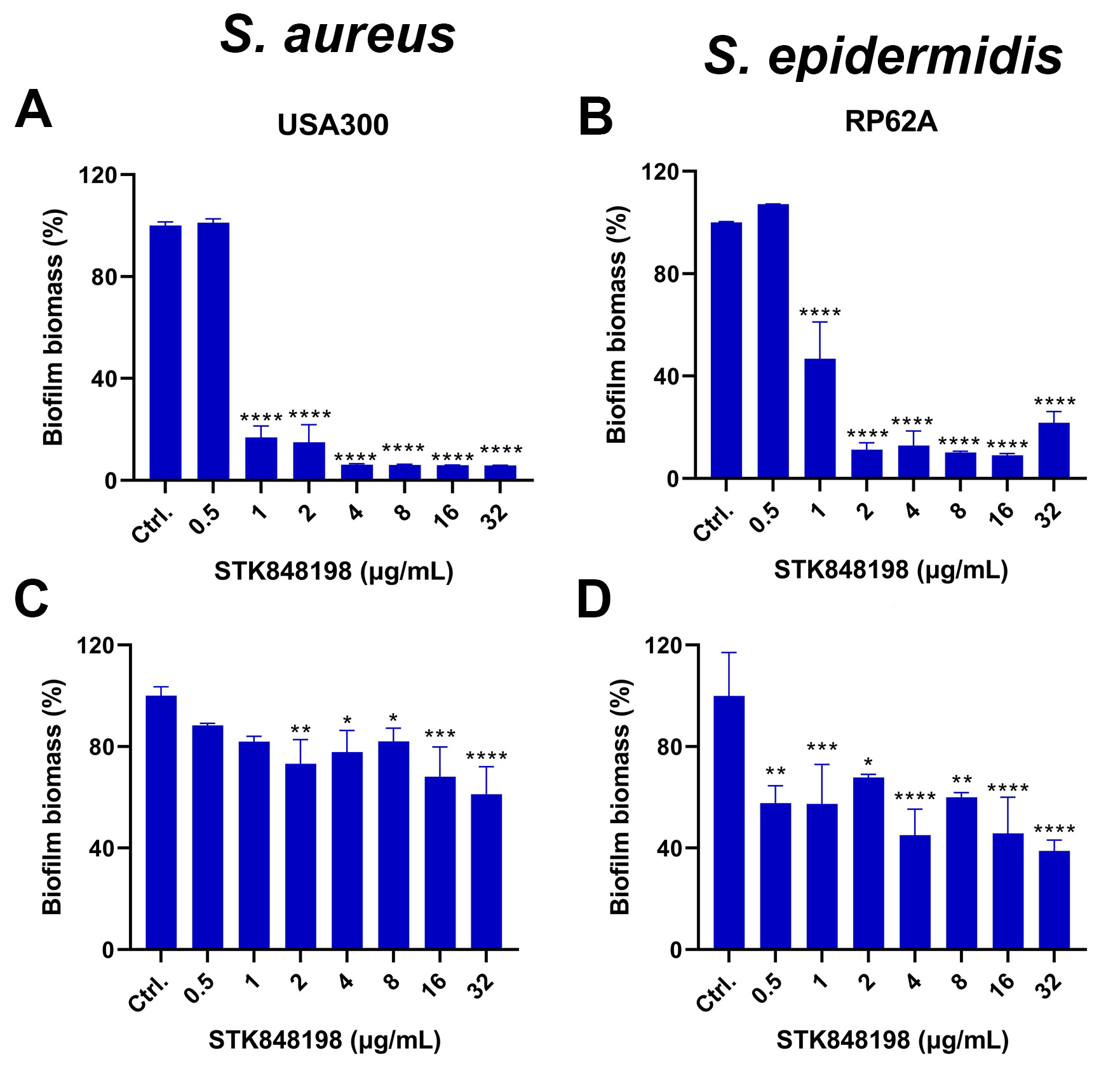


**Supplementary Figure 6. Antibiofilm activity of STK848198 against *S. aureus* and *S. epidermidis* determined by crystal violet staining.** (A) Biofilm inhibitory effects of STK848198 against *S. aureus* USA300. (B) Biofilm inhibitory effects of STK848198 against *S. epidermidis* RP62A. (C) Biofilm eradication effects of STK848198 against *S. aureus* USA300. (B) Biofilm eradication effects of STK848198 against *S. epidermidis* RP62A. *: *P*<0.05. **: *P*<0.01. ***: *P*<0.001. ****: *P*<0.0001.


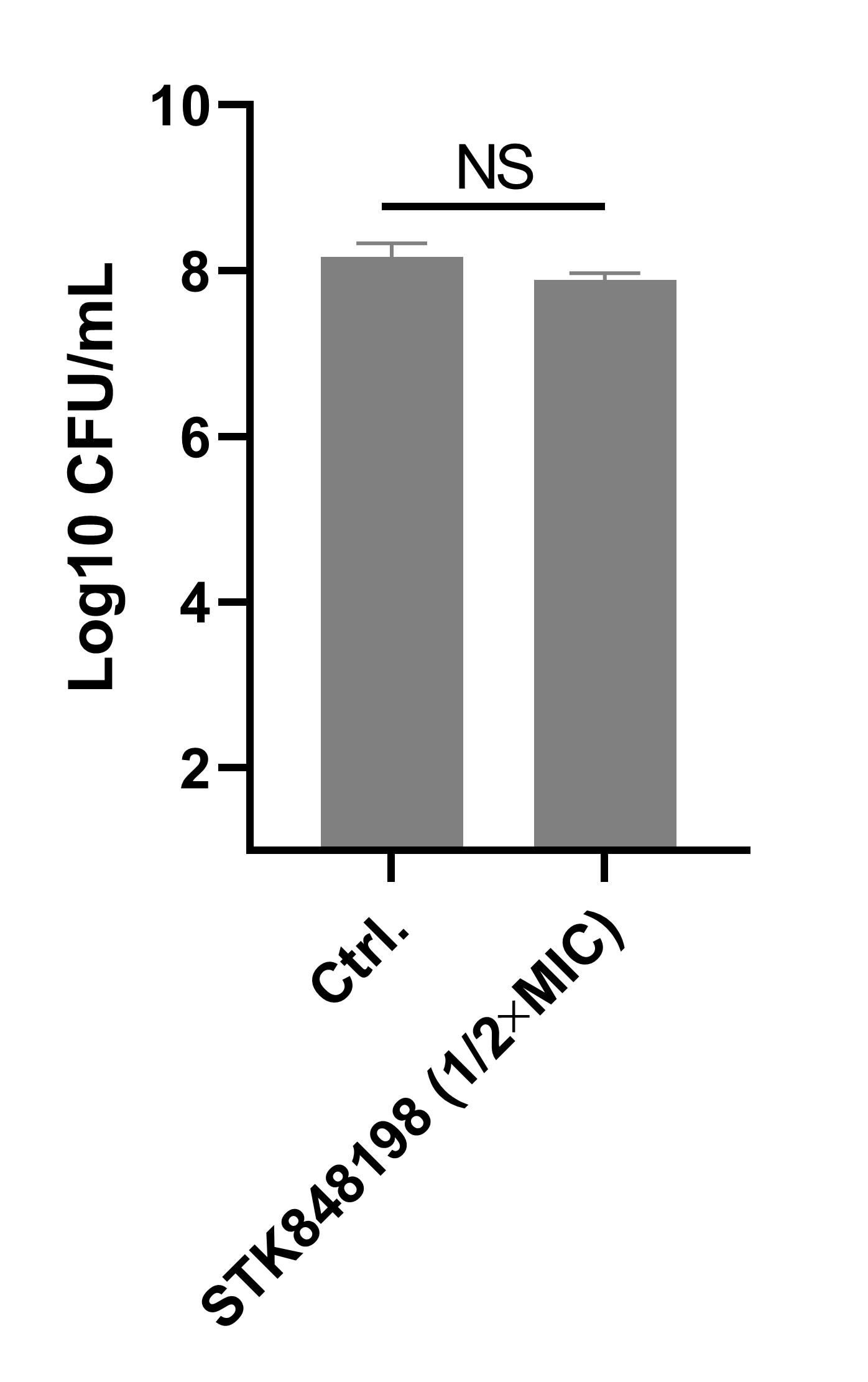


**Supplementary Figure 7.** Viable cell counts of *S. aureus* ATCC 43300 after treated with 1/2×MIC of STK848198 for 1h. NS: no statistical difference.


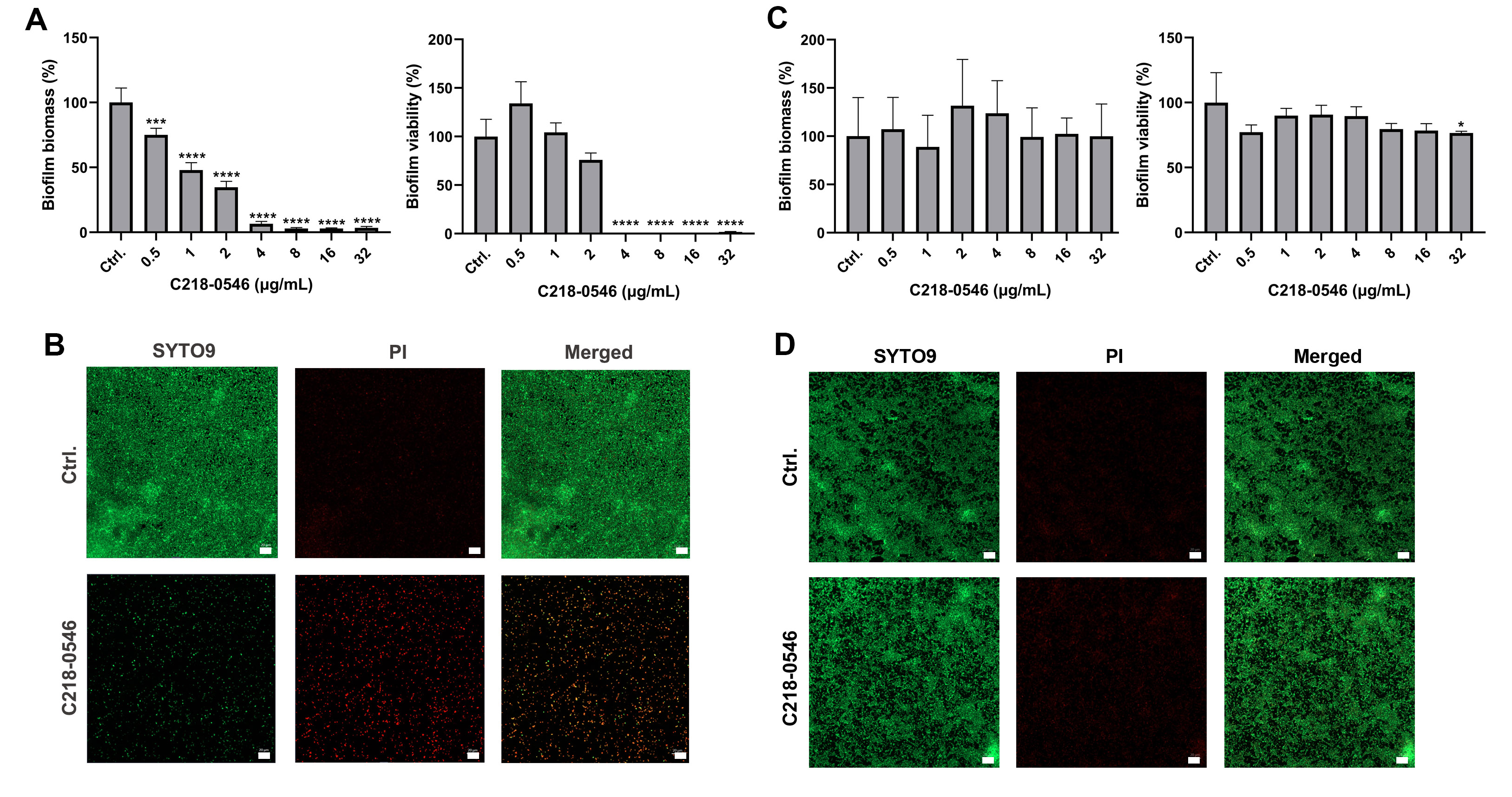


**Supplementary Figure 8. Biofilm inhibiting but no eradicating activity by C218-0546.** (A) Biofilm inhibitory effects of C218-0546 against *S. aureus* ATCC 43300 determined by crystal violet staining (left panel) and XTT staining (right panel), respectively. (B) Biofilm inhibitory observation after treated with 4 μg/mL of C218-0546. The biofilms were stained with SYTO9/PI and observed by CLSM. Scale: 20 μm. (C) No biofilm eradication was detected of C218-0546 by crystal violet staining (left panel) and XTT staining (right panel), respectively. (D) Biofilm eradication after treated with 16 μg/mL of C218-0546 and observed by CLSM. Scale: 20 μm. *: *P*<0.05. **: *P*<0.01. ***: *P*<0.001. ****: *P*<0.0001.


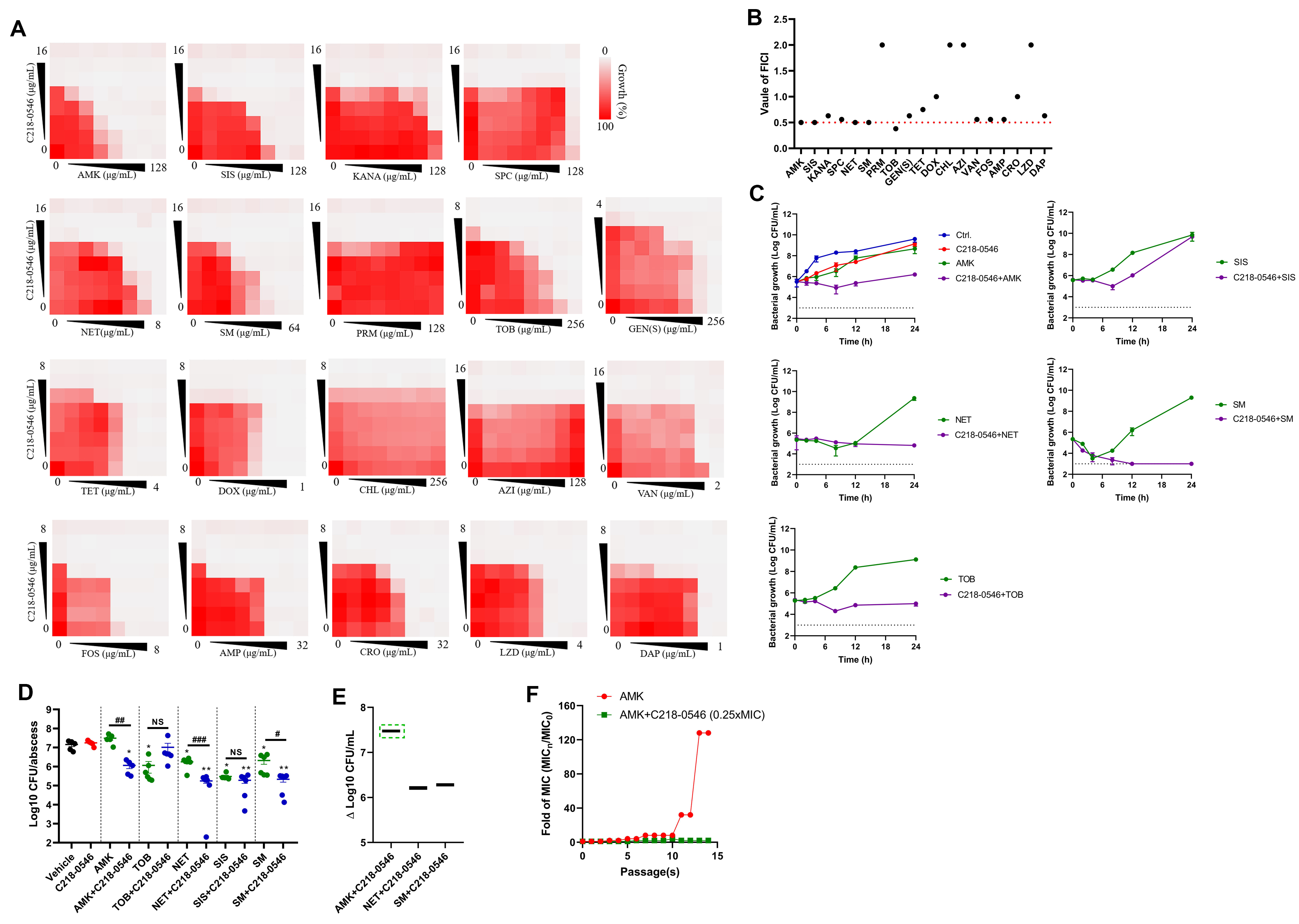


**Supplementary Figure 9. Combinational antimicrobial effects between C218-0546 and conventional antibiotics.** (A) Drug combination determined by checkerboard micro-dilution assay. (B) FICI values distribution of the checkerboard micro-dilution assay. (C) Synergistic antimicrobial efficacy between C218-0546 (1 μg/mL) and AMK (8 μg/mL), SIS (16 μg/mL), NET (2 μg/mL), SM (8 μg/mL), or TOB (32 μg/mL), respectively, determined by time-killing assay. (D) Antimicrobial synergy determination *in vivo* by abscess infection model. (E) Quantification of the bacterial loads reduction in the abscess with statistical differences. (F) Reduced resistance inducing ability of AMK in the presence of sub-MIC (0.25×MIC) of C218-0546. *: *P*<0.05. **: *P*<0.01. ***: *P*<0.001. ****: *P*<0.0001.


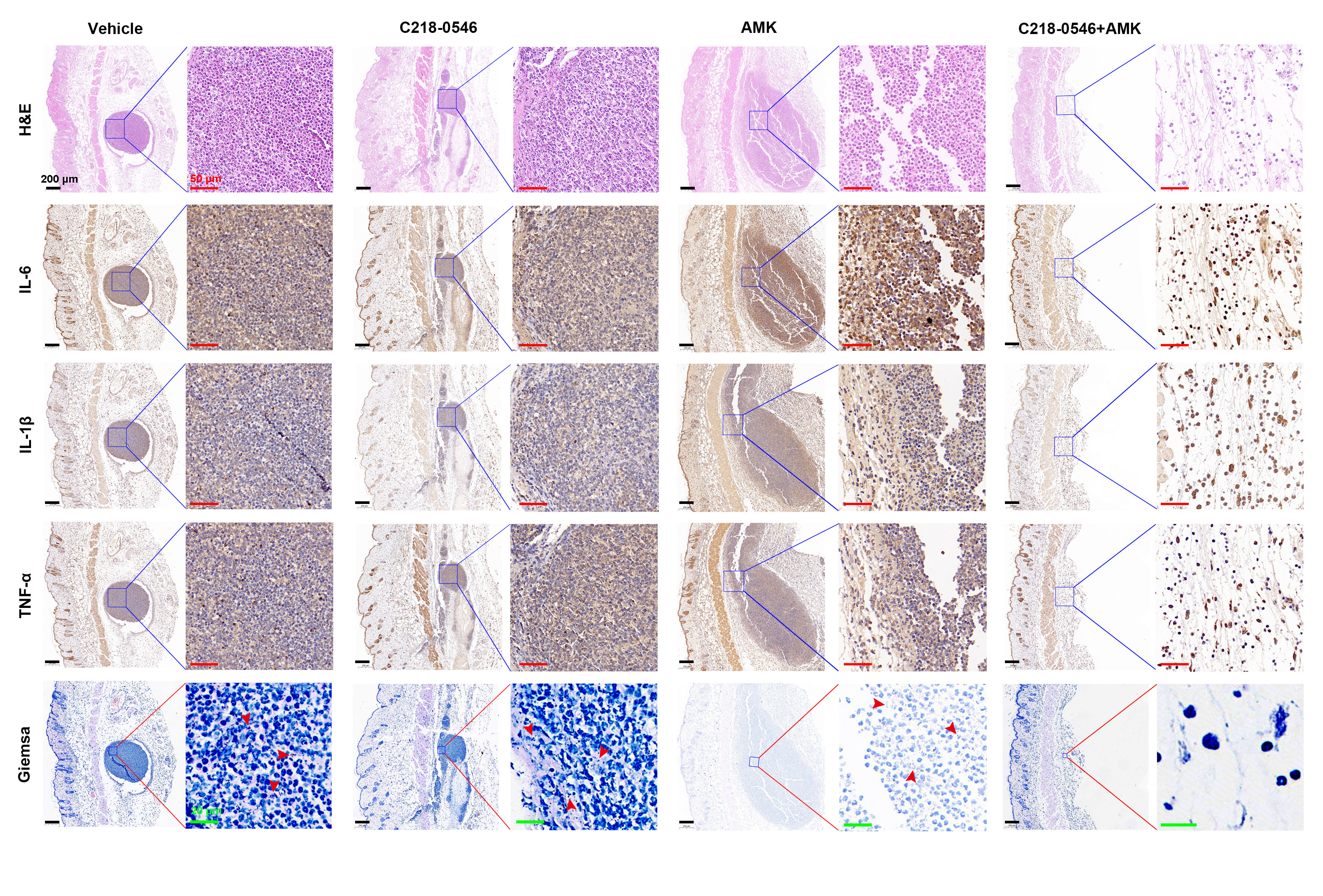


**Supplementary Figure 10. Pathological analysis of the abscess after treatment with AMK alone or in combination with C218-0546.** Black scale: 200 μm. Red scale: 50 μm. Green scale: 20 μm.


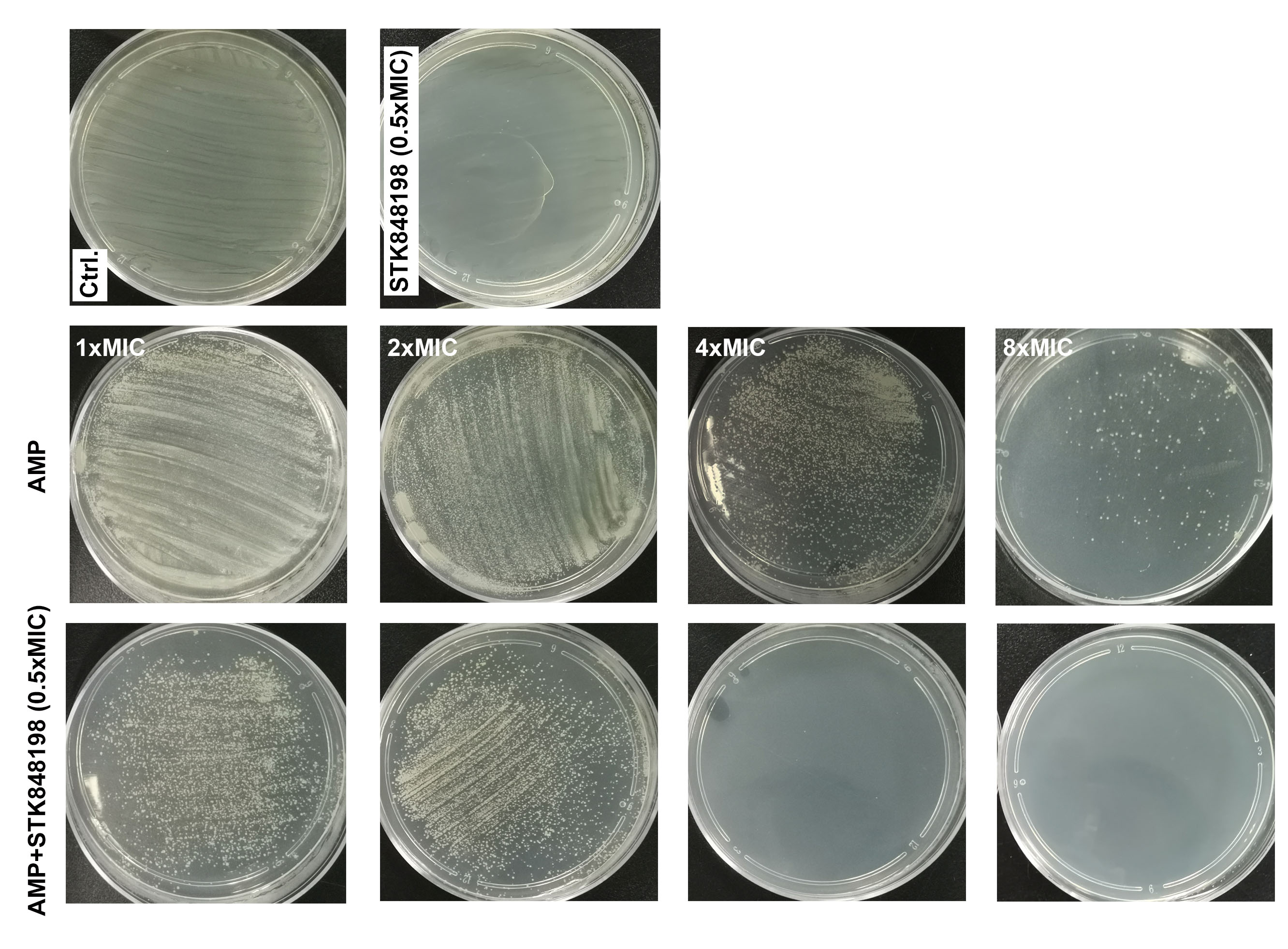


**Supplementary Figure 11.** One-step resistance inducing by AMP alone or in the presence of sub-MIC (0.5×MIC) of STK848198.


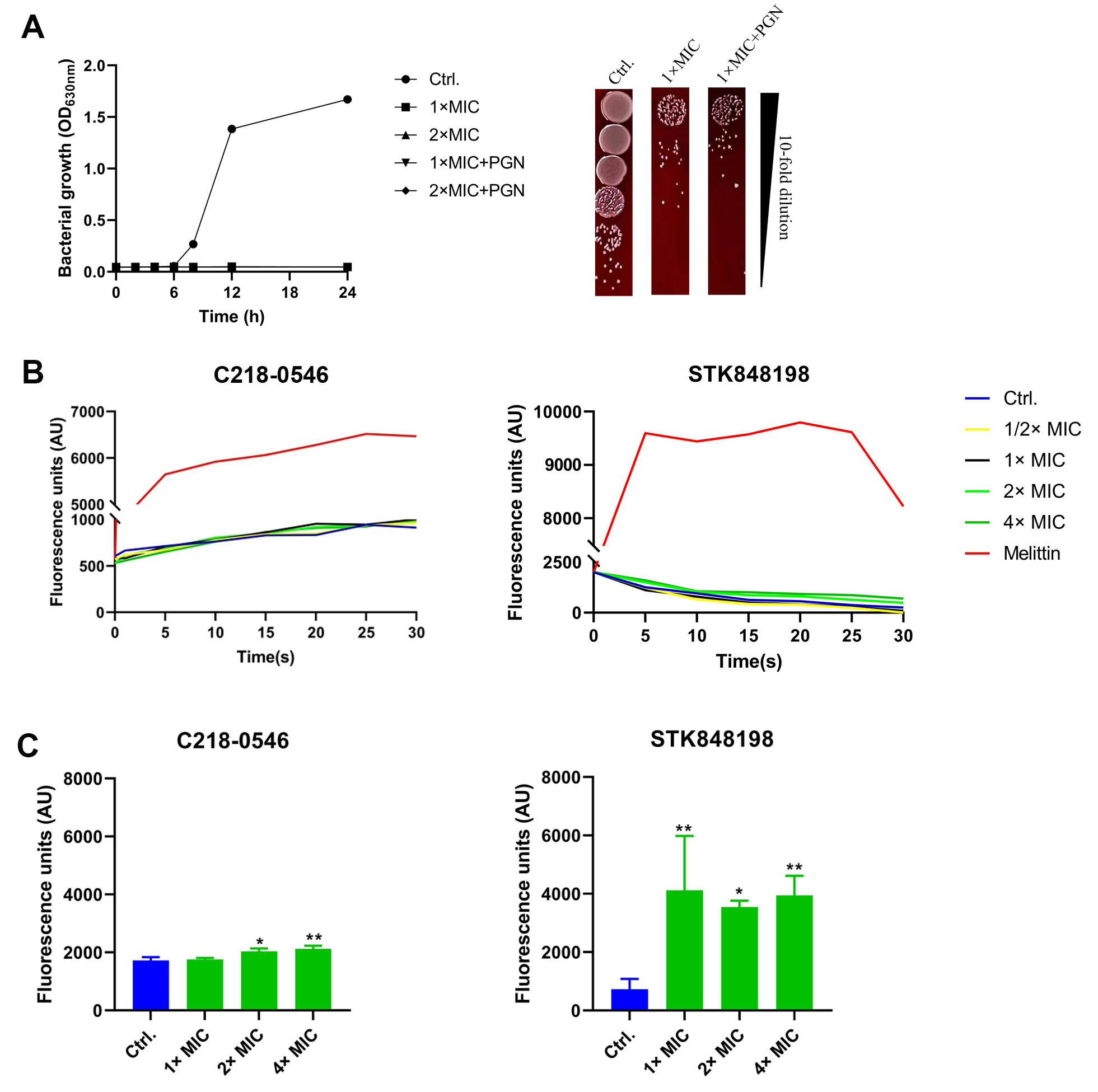


**Supplementary Figure 12.** **Other potential targets of C218-0546/STK848198.** (A) Growth curve (left panel) and 24h viable cell count (right panel) of *S. aureus* ATCC 43300 in the presence or absence of 40 μg/mL PGN. (B) Cell membrane permeability detection by SYTOX Green. Melittin (16 μg/mL) was used as a positive control. (C) ROS quantification by DCFH-DA probe. *: *P*<0.05. **: *P*<0.01. ***: *P*<0.001. ****: *P*<0.0001.


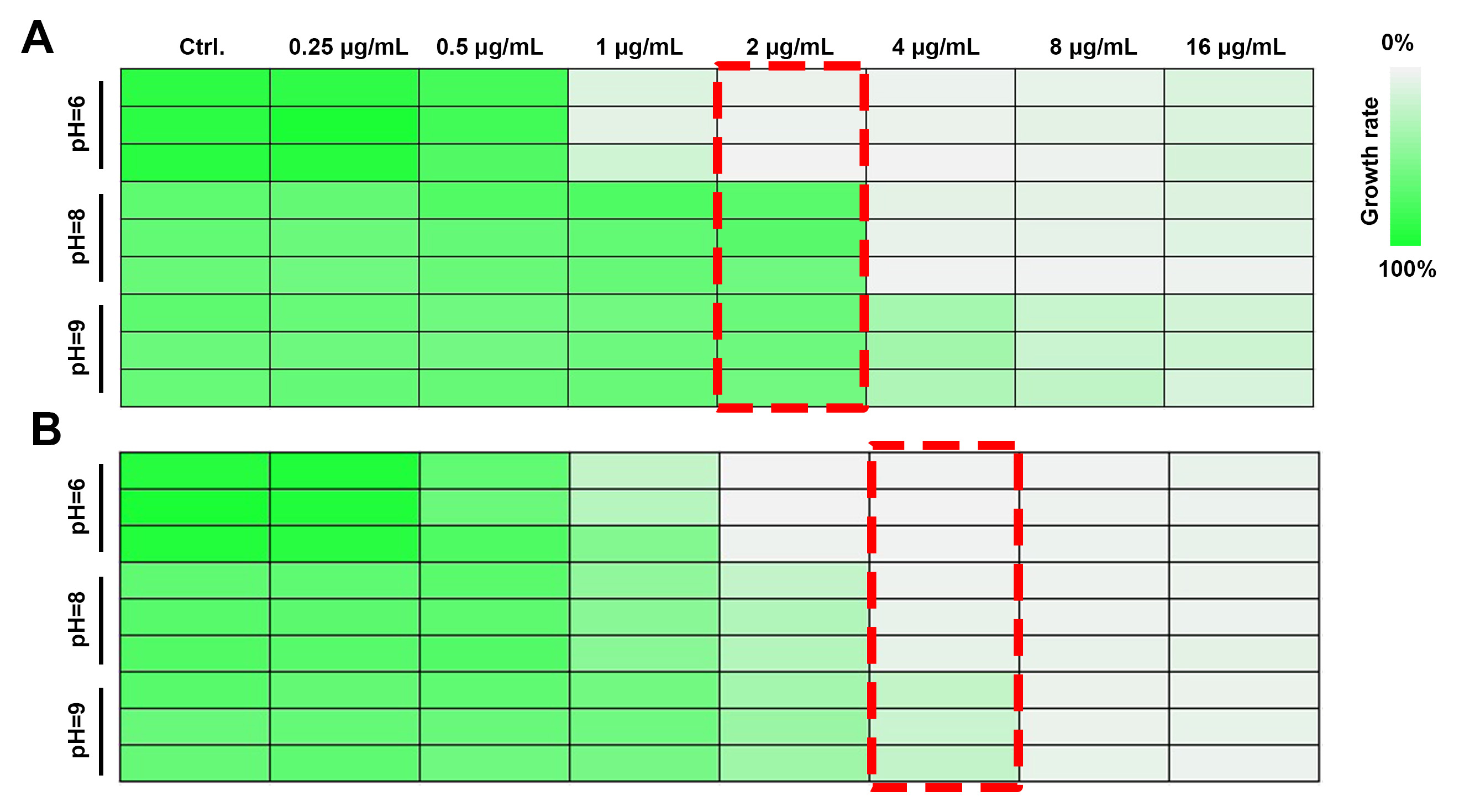


**Supplementary Figure 13. Growth inhibition effects of C218-0546/STK848198 in the presence of varied pH values.** Log-phased *S. aureus* ATCC 43300 were treated with serially diluted C218-0546 (A) or STK848198 (B) for 16h in MH broth in the presence of varied pH values. The bacterial growth turbidity was determined by measuring the OD630nm.


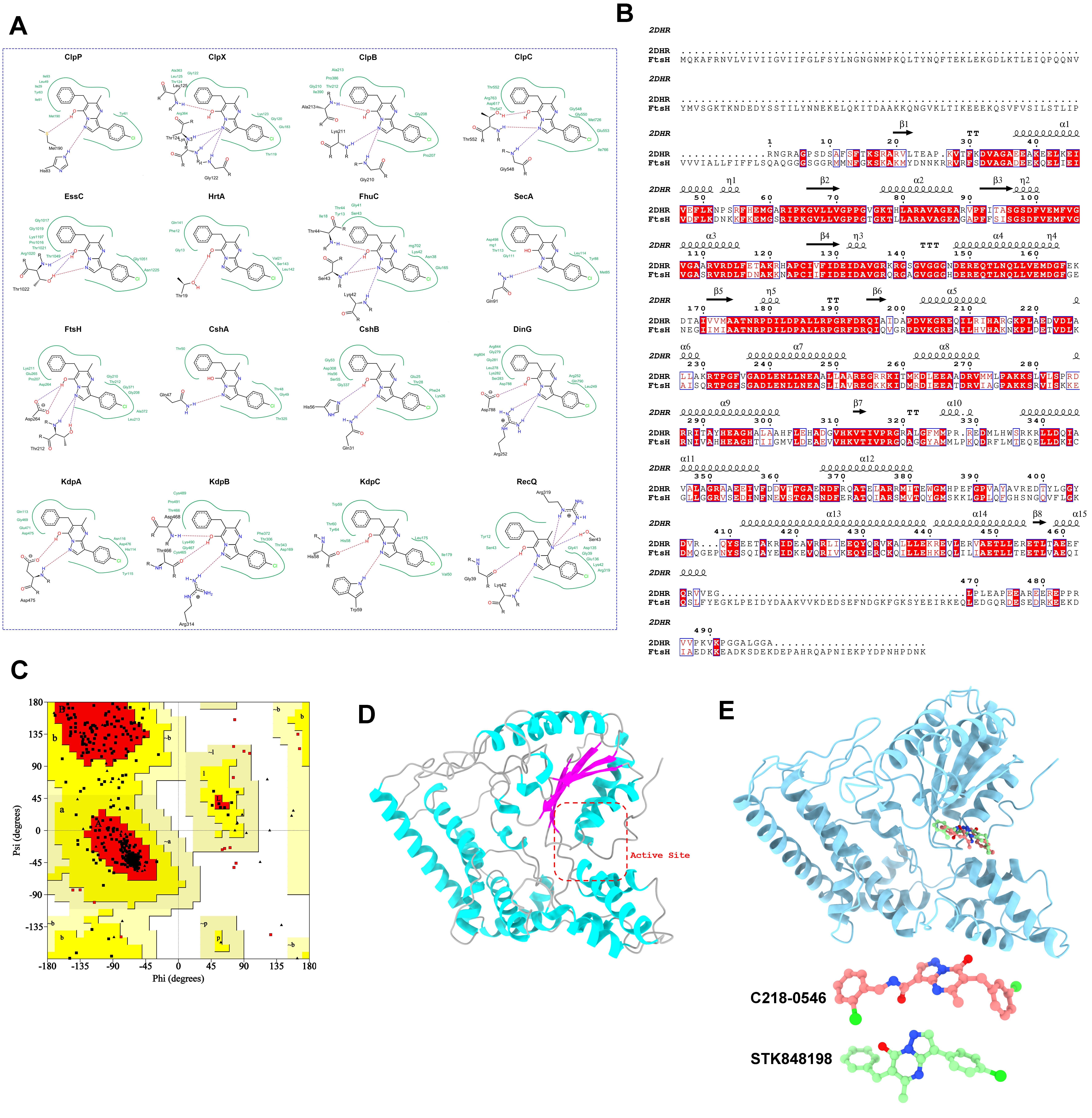


**Supplementary Figure 14. Homology modeling of FtsH.** (A) Binding models between STK848198 and candidate targets by molecule docking. (B) Amino acid alignment between FtsH and homology protein (PBD: 2DHR). (C) Ramachandran plot of FtsH. (D) Structural model of FtsH. Red dash line indicated active site. (E) Structure of molecules and its binding model with FtsH.


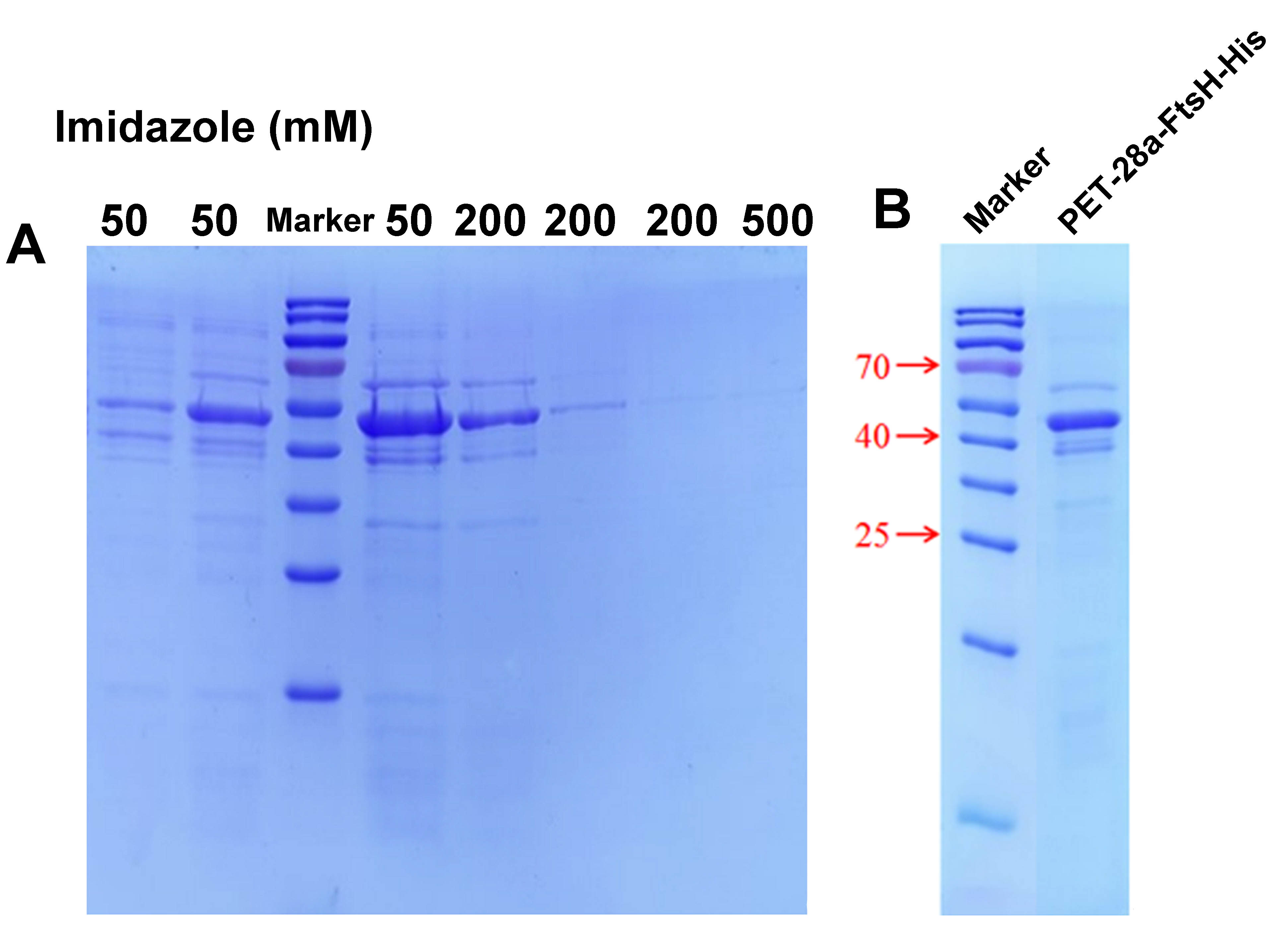


**Supplementary Figure 15. FtsH synthesis and purification.** (A) FtsH elution with indicated concentrations of imidazole. (B) FtsH identification.


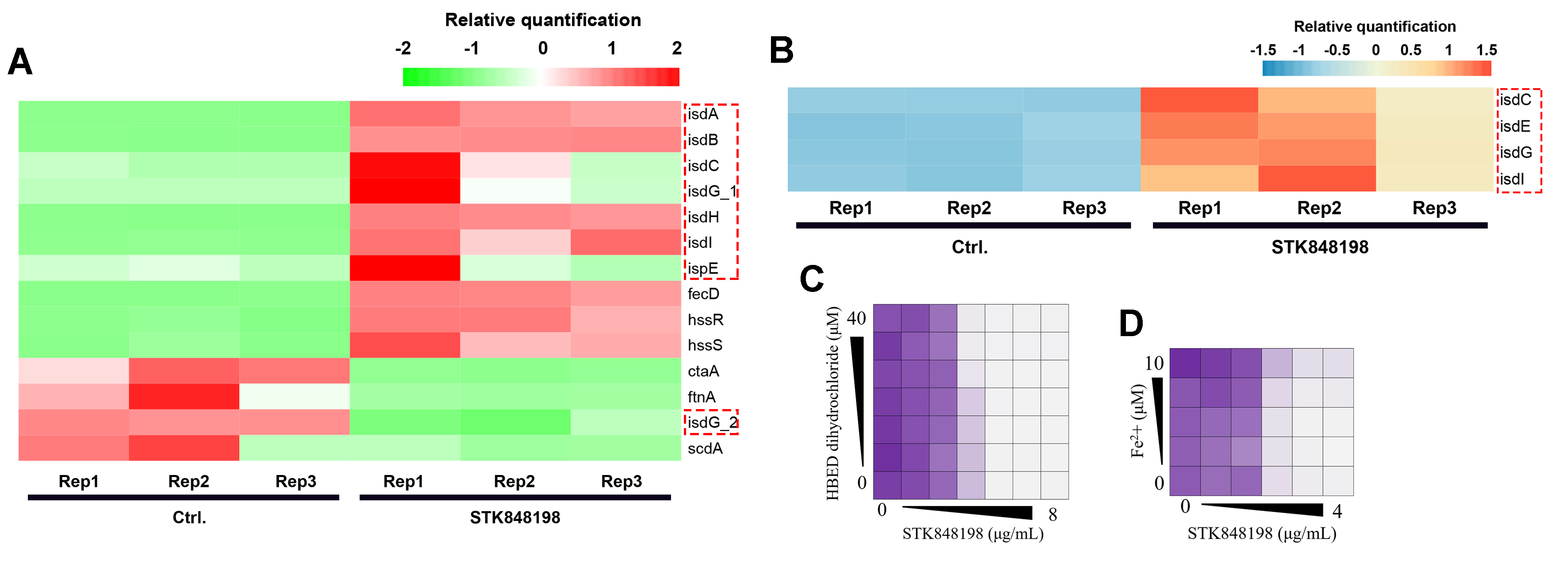


**Supplementary Figure 16**. **Effects of STK848198 on ferric ion metabolism.** Ferric ion metabolism related transcriptomic (A) and proteomic (B) analysis of *S. aureus* ATCC 43300 after treated with 5 ×MIC of STK848198 for 1h. (C) Effects of ferric iron chelator HBED dihydrochloride on the antimicrobial effects of STK848198. (D) Effects of exogenous ferrous ion on the antimicrobial effects of STK848198.


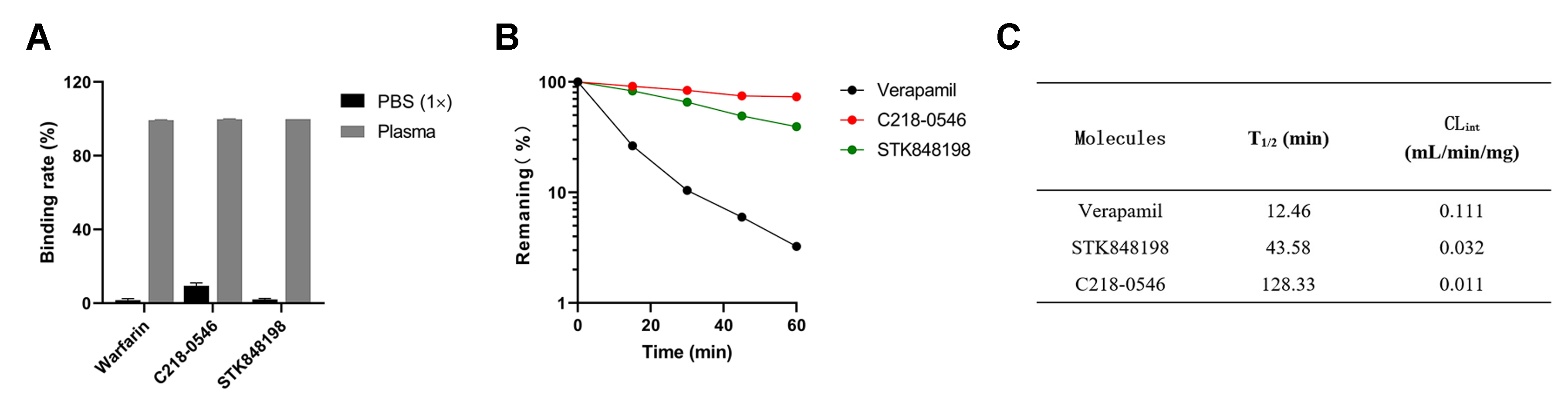


**Supplementary Figure 17**. **Plasma protein binding ability and metabolism in liver microsomes.** (A) Plasma protein binding ratio by C218-0546/STK848198. Warfarin was used as a positive control. (B) Enzyme kinetics of C218-0546/STK848198 metabolism in the presence of mice liver microsomes. (C) Half-life and clearance rate calculation.


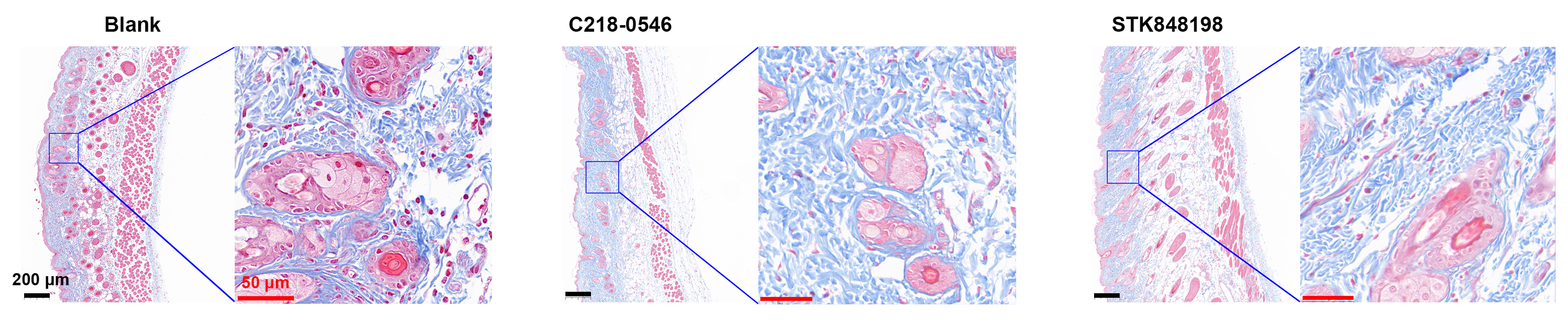


**Supplementary Figure 18. Collagen fibers observation by Masson staining.** The mice were treated with 30 mg/kg of C218-0546 and STK848198 by s.c. injection, respectively. Black scale: 200 μm. Red scale: 50 μm.


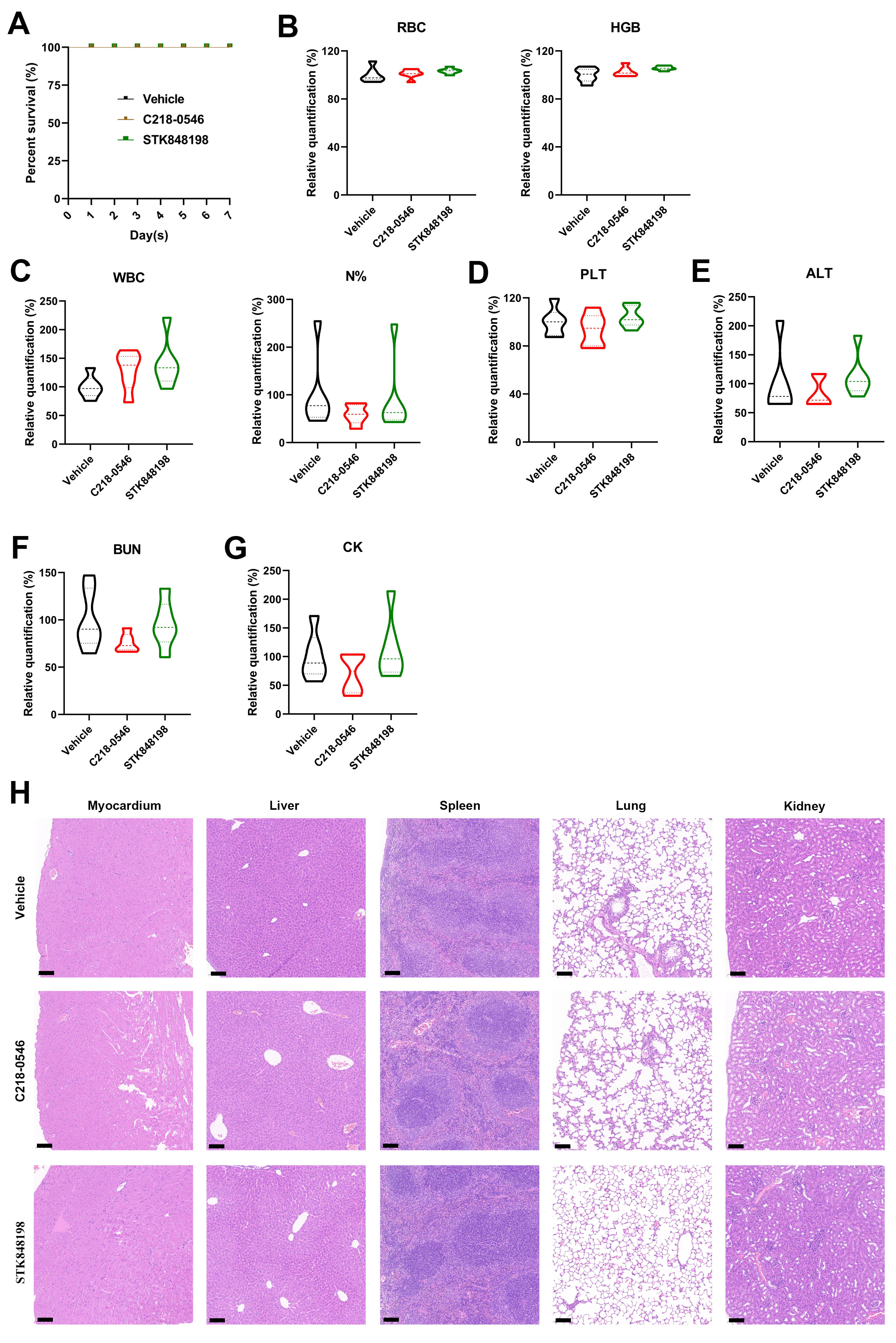


**Supplementary Figure 19.** ***In vivo* one-day toxicity by C218-0546 and STK848198.** (A) Mice survival rate after consecutive treatment of 100 mg/kg of molecules. (B) RBC-related parameters of RBC counting (left panel) and hemoglobin (HGB) quantification (right panel). (C) White blood cell (WBC)-related parameters of WBC counting (left panel) and neutrophils proportion (N%, right panel). (D) Platelet (PLT) quantification. (E) Quantification of liver biomarker glutamic-pyruvic transaminase (ALT). (F) Quantification of renal biomarker blood urea nitrogen (BUN). (G) Quantification of myocardial biomarker creatine kinase (CK). (H) H&E staining of organs.


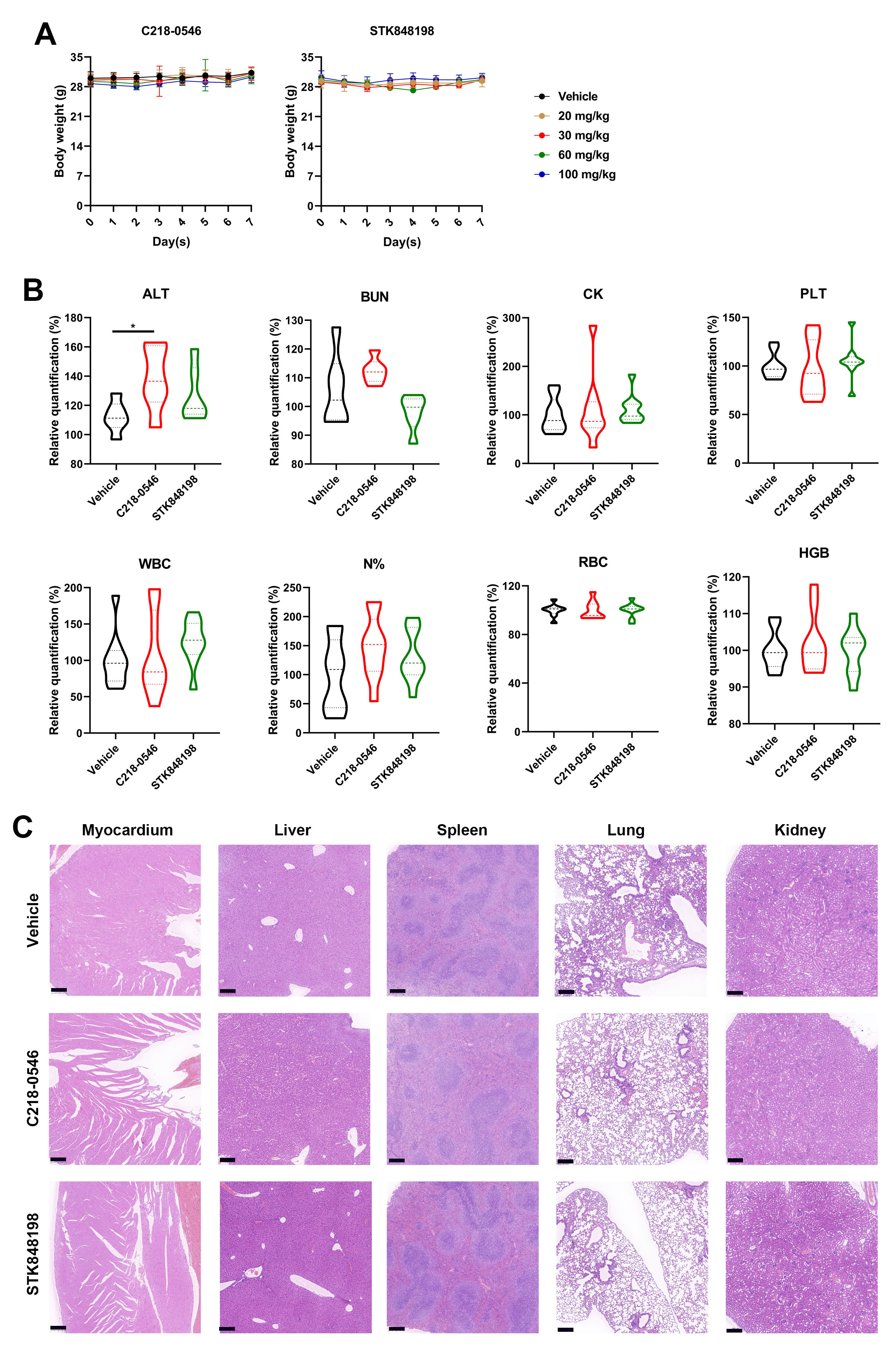


**Supplementary Figure 20. *In vivo* 7-day toxicity by C218-0546 and STK848198.** (A) Mice body weight monitoring during 7 days of consecutive treatment. (B) Mice biomarkers quantification at the 7^th^ day. *: *P*<0.05. No statistical difference was found except for ALT. (C) H&E staining of the mice organs after 7 days of treatment. Scale: 200 μm.


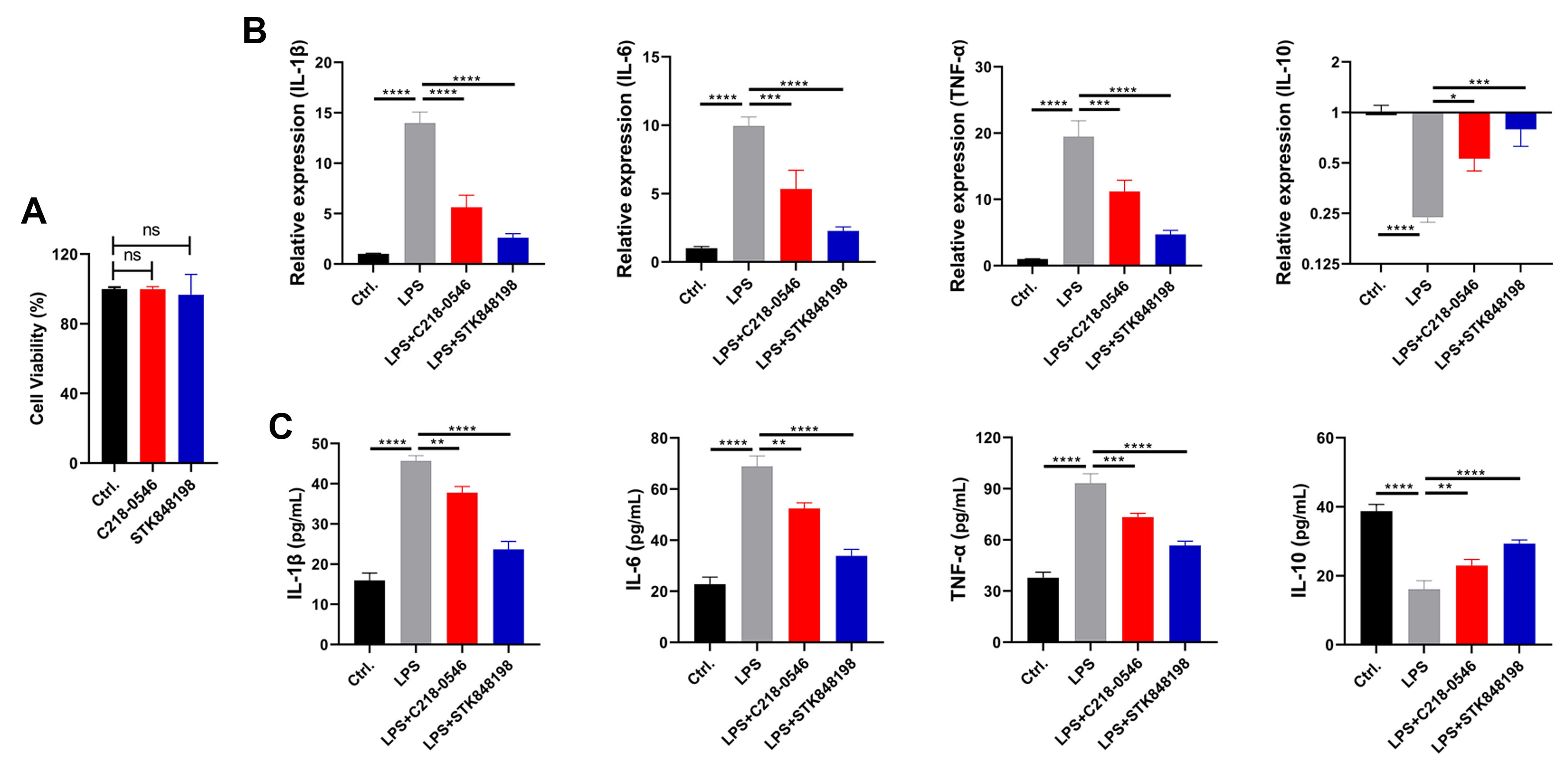


**Supplementary Figure 21. Inflammation inhibitory effects of C218-0546 and STK848198.** (A) RAW264.7 viability after treated with 16 μg/mL of C218-0546 and STK848198 for 24h. (B) Cytokines expression quantification by qRT-PCR after treated with C218-0546/STK848198 (16 μg/mL) for 12h. (C) Cytokines production quantification by ELISA after treated with C218-0546/STK848198 (16 μg/mL) for 12h. *: *P*<0.05. **: *P*<0.01. ***: *P*<0.001. ****: *P*<0.0001.

**Supplementary Tables**

**Supplementary Table 1. ADME/T prediction of C218-0546 and STK848198**

| Parameters | C218-0546 | STK848198 |
| --- | --- | --- |
| Lipinski Rule of Five_Score | 0.973 | 0.915 |
| Oral Non CNS Scoring Profile_Score | 0.021 | 0.021 |
| LogP | 4.302 | 4.583 |
| LogD | 4.302 | 4.583 |
| BBB log([brain]:[blood]) | -0.591 | -1.244 |
| HIA category | + | + |
| 2C9 p^Ki^ | 6.26 | 6.068 |
| 2D6 affinity category | Medium | Medium |
| HBD | 2 | 1 |
| HBA | 6 | 4 |
| TPSA | 79.52 | 50.42 |
| Rotatable Bonds | 6 | 3 |
| Flexibility | 0.182 | 0.107 |
| hERG p^IC^_50_ | 5.142 | 5.643 |
| Ames Toxicity | Non | Non |
| Carcinogens | Non | Non |

BBB: blood-brain barrier. Number of hydrogen bond donors. HBA: Number of hydrogen bond acceptors. TPSA: Topological polar surface area.

**Supplementary Table 2. Antimicrobial activity of molecules against clinical isolates**

| Strains | VAN | DAP |
| --- | --- | --- |
| ATCC 43300 | 1 | 1 |
| USA300 | 1 | 1 |
| ATCC 25923 | 1 | 1 |
| Newman | 2 | 2 |
| LZB1 | 1 | 1 |
| SAJ1 | 4 | 32 |
| SA1901 | 1 | 1 |
| SA1902 | 0.5 | 1 |
| SA1909 | 1 | 1 |
| SA1910 | 1 | 1 |
| SA1911 | 1 | 2 |
| SA1912 | 1 | 1 |
| SA1913 | 1 | 1 |
| SA1914 | 1 | 2 |
| SA1915 | 1 | 1 |
| R82174 | 1 | 2 |
| R0645 | 1 | 2 |

**Supplementary Table 3.** **Spontaneous resistance frequencies of antimicrobials against *S. aureus* ATCC 43300**

| Antimicrobial | Spontaneous resistance frequency (mean±SD) | | |
| --- | --- | --- | --- |
|  | 1◊MIC | 2◊MIC | 4◊MIC |
| RFP | (2.83±2.9) ×10^-7^ | （1.65±1.28) ×10^-7^ | （6.27±6.92) ×10^-8^ |
| C218-0546 | （1.41±1.05) ×10^-8^ | （1.79±2.28) ×10^-8^ | （2.56±5.72) ×10^-9^ |
| STK848198 | N.D. | N.D. | N.D. |

N.D.: Not detectable.

**Supplementary Table 4. MIC values of C218-0546/STK848198 against conditional pathogens**

| Strains | C218-0546 (μg/mL) | STK848198 (μg/mL) |
| --- | --- | --- |
| *S. epidermidis* |  |  |
| ATCC 12228^a^ | 4 | 1 |
| RP62A^b^ | 4 | 1 |
| *E. faecalis* |  |  |
| ATCC 29212 | 4 | 4 |
| ATCC 51299^c^ | 4 | 4 |
| *E. faecium* |  |  |
| ATCC 19434 | >32 | 8 |
| U101^c^ | 2 | 4 |
| *E. coli* |  |  |
| ATCC 25922 | >32 | >32 |
| *P. aeruginosa* |  |  |
| PAO1 | >32 | >32 |
| *A. baumannii* |  |  |
| ATCC 19606 | >32 | >32 |
| *K. pneumoniae* |  |  |
| ATCC 700603 | >32 | >32 |

a: biofilm formation positive strain; b: biofilm formation negative strain; c: vancomycin resistant strain.

| **Supplementary Table 5. Ames test of C218-0546** | | | | | | |
| --- | --- | --- | --- | --- | --- | --- |
| Strains | Groups | Concentration | -S9 | | +S9 | |
|  |  | (μg/well) | CFU($\bar{x}$±SD) | Fold Change | CFU($\bar{x}$±SD) | Fold Change |
| TA 97a | Background | - | 26±1 | N/A | 26±1 | N/A |
|  | Negative | - | 25±3 | 1 | 25±3 | 0.9 |
|  | Positive | - | 901±24 | 36.5 | 587±40 | 23.8 |
|  | C218-0546 | 400 | 13±5 | 0.5 | 14±4 | 0.6 |
|  |  | 200 | 16±4 | 0.6 | 16±3 | 0.6 |
|  |  | 100 | 16±6 | 0.6 | 16±5 | 0.7 |
|  |  | 50 | 16±4 | 0.7 | 19±3 | 0.8 |
|  |  | 25 | 19±3 | 0.8 | 18±3 | 0.7 |
| TA 98 | Background | - | 6±1 | N/A | 6±1 | N/A |
|  | Negative | - | 6±1 | 1 | 7±1 | 1.1 |
|  | Positive | - | 651±128 | 108.4 | 1349±72 | 202.4 |
|  | C218-0546 | 400 | 5±1 | 0.9 | 6±1 | 1 |
|  |  | 200 | 5±1 | 0.8 | 5±1 | 0.8 |
|  |  | 100 | 5±1 | 0.8 | 5±1 | 0.8 |
|  |  | 50 | 5±2 | 0.9 | 4±1 | 0.7 |
|  |  | 25 | 6±1 | 1 | 6±1 | 0.9 |
| TA 100 | Background | - | 34±3 | N/A | 33 ±6 | N/A |
|  | Negative | - | 33±3 | 1.0 | 35 ±2 | 1.1 |
|  | Positive | - | 776±30 | 23.5 | 724 ±37 | 20.5 |
|  | C218-0546 | 400 | 14±2 | 0.4 | 18 ±1 | 0.5 |
|  |  | 200 | 20±3 | 0.6 | 14 ±4 | 0.4 |
|  |  | 100 | 16±3 | 0.5 | 16 ±3 | 0.5 |
|  |  | 50 | 14±1 | 0.4 | 15 ±3 | 0.4 |
|  |  | 25 | 17±5 | 0.5 | 16 ±4 | 0.5 |
| WP2*uvrA* (pKM101) | Background | - | 24±2 | N/A | 31 ±4 | N/A |
|  | Negative | - | 23±1 | 1 | 32 ±4 | 1 |
|  | Positive | - | 211±20 | 9.2 | 207 ±24 | 6.4 |
|  | C218-0546 | 400 | 20±2 | 0.9 | 26 ±2 | 0.8 |
|  |  | 200 | 27±5 | 1.2 | 29 ±5 | 0.9 |
|  |  | 100 | 21±2 | 0.9 | 28 ±6 | 0.9 |
|  |  | 50 | 22±5 | 1 | 30 ±4 | 0.9 |
|  |  | 25 | 23±2 | 1 | 28 ±3 | 0.9 |
| TA 1535 | Background | - | 3±2 | N/A | 3±1 | N/A |
|  | Negative | - | 3±2 | 1 | 2±2 | 0.9 |
|  | Positive | - | 416±121 | 156 | 181±37 | 77.7 |
|  | C218-0546 | 400 | 3±2 | 1.3 | 3±2 | 1.3 |
|  |  | 200 | 3±1 | 1.3 | 3±1 | 1.1 |
|  |  | 100 | 2±0 | 0.8 | 3±1 | 1.3 |
|  |  | 50 | 2±2 | 0.9 | 3±1 | 1.3 |
|  |  | 25 | 3±1 | 1.1 | 3±1 | 1.3 |

**Supplementary Table 6. Ames test of STK848198**

| Strains | Groups | Concentration | -S9 | | +S9 | |
| --- | --- | --- | --- | --- | --- | --- |
|  |  | (μg/well) | CFU($\bar{x}$±SD) | Fold Change | CFU($\bar{x}$±SD) | Fold Change |
| TA 97a | Background | - | 26±1 | N/A | 26±1 | N/A |
|  | Negative | - | 25±3 | 1 | 25±3 | 0.9 |
|  | Positive | - | 901±24 | 36.5 | 587±40 | 23.8 |
|  | STK848198 | 400 | 4±3 | 0.2 | 5±2 | 0.2 |
|  |  | 200 | 10±4 | 0.4 | 4±1 | 0.2 |
|  |  | 100 | 8±2 | 0.3 | 4±4 | 0.2 |
|  |  | 50 | 11±1 | 0.4 | 6±2 | 0.2 |
|  |  | 25 | 14±2 | 0.6 | 6±5 | 0.3 |
| TA 98 | Background | - | 6±1 | N/A | 6±1 | N/A |
|  | Negative | - | 6±1 | 1 | 7±1 | 1.1 |
|  | Positive | - | 651±128 | 108.4 | 1349 ±72 | 202.4 |
|  | STK848198 | 400 | 0±1 | 0.1 | 4 ±0 | 0.6 |
|  |  | 200 | 1±2 | 0.2 | 5 ±1 | 0.7 |
|  |  | 100 | 3±2 | 0.5 | 4 ±1 | 0.7 |
|  |  | 50 | 5±1 | 0.8 | 5 ±1 | 0.8 |
|  |  | 25 | 5±3 | 0.8 | 6 ±1 | 0.9 |
| TA 100 | Background | - | 34±3 | N/A | 33±6 | N/A |
|  | Negative | - | 33±3 | 1 | 35±2 | 1.1 |
|  | Positive | - | 776±30 | 23.5 | 724±37 | 20.5 |
|  | STK848198 | 400 | 4±2 | 0.1 | 8 ±2 | 0.2 |
|  |  | 200 | 9±3 | 0.3 | 8±3 | 0.2 |
|  |  | 100 | 5±2 | 0.1 | 9 ±3 | 0.3 |
|  |  | 50 | 6±2 | 0.2 | 7 ±1 | 0.2 |
|  |  | 25 | 5±2 | 0.2 | 8 ±2 | 0.2 |
| WP2*uvrA* (pKM101) | Background | - | 24±2 | N/A | 31 ±4 | N/A |
|  | Negative | - | 23±1 | 1 | 32 ±4 | 1 |
|  | Positive | - | 211±20 | 9.2 | 207 ±24 | 6.4 |
|  | STK848198 | 400 | 23±1 | 1 | 28 ±3 | 0.9 |
|  |  | 200 | 24±3 | 1 | 29 ±3 | 0.9 |
|  |  | 100 | 21±1 | 0.9 | 26 ±3 | 0.8 |
|  |  | 50 | 23±4 | 1 | 26 ±4 | 0.8 |
|  |  | 25 | 22±2 | 1 | 29 ±4 | 0.9 |
| TA 1535 | Background | - | 3±2 | N/A | 3±1 | N/A |
|  | Negative | - | 2±1 | 0.9 | 2±2 | 0.9 |
|  | Positive | - | 416±121 | 178.3 | 181±37 | 77.7 |
|  | STK848198 | 400 | 1±1 | 0.6 | 2±1 | 0.7 |
|  |  | 200 | 1±1 | 0.6 | 2±1 | 0.7 |
|  |  | 100 | 2±2 | 1 | 2±1 | 1 |
|  |  | 50 | 2±2 | 1 | 2±1 | 0.9 |
|  |  | 25 | 2±1 | 0.9 | 2±1 | 0.9 |

**Supplementary Table 7. The pharmacokinetic parameters of C218-0546**

| Route | mg/kg | T1/2(h) | Tmax(h) | Cmax(μg/mL) | AUC(0-t) | AUC(0-∞） | MRT(0-t) | MRT(0-∞) | C0 | Vss | Vz | Cl | F(%) |
| --- | --- | --- | --- | --- | --- | --- | --- | --- | --- | --- | --- | --- | --- |
|  |  |  |  |  | h*μg/mL | h*μg/mL | h | h | μg/mL | L/kg | L/kg | mL/min/kg |  |
| i.v. | 20 | 1.08±0.57 | 0.08±0.00 | 106.48±6.39 | 56.60±14.45 | 56.72±14.43 | 0.51±0.10 | 0.53±0.09 | 143.32±13.14 | 0.19±0.01 | 0.59±0.38 | 6.11±1.36 | ‐ |
| p.o. | 20 | 1.41±0.08 | 0.50±0.00 | 10.91±1.65 | 13.24±1.60 | 13.36±1.59 | 1.18±0.11 | 1.26±0.13 | ‐ | ‐ | ‐ | ‐ | 23.40±2.83 |
| s.c. | 20 | 2.38±2.20 | 2.33±1.53 | 2.80±0.37 | 15.60±0.78 | 14.19±12.37 | 3.59±0.27 | 4.10±3.70 | ‐ | ‐ | ‐ | ‐ | 27.57±1.38 |
| i.p. | 20 | 0.98±0.23 | 1.33±0.58 | 12.38±2.73 | 29.64±6.17 | 29.78±6.14 | 1.76±0.11 | 1.80±0.12 | ‐ | ‐ | ‐ | ‐ | 52.37±10.90 |

**Supplementary Table 8. The pharmacokinetic parameters of STK848198**

| Route | mg/kg | T1/2(h) | Tmax(h) | Cmax(μg/mL) | AUC(0-t) | AUC(0-∞） | MRT(0-t) | MRT(0-∞) | C0 | Vss | Vz | Cl | F(%) |
| --- | --- | --- | --- | --- | --- | --- | --- | --- | --- | --- | --- | --- | --- |
|  |  |  |  |  | h*μg/mL | h*μg/mL | h | h | μg/mL | L/kg | L/kg | mL/min/kg |  |
| i.v. | 20 | 2.54±0.70 | 0.08±0.00 | 40.11±2.18 | 22.01±1.96 | 22.39±1.99 | 0.67±0.10 | 0.86±0.05 | 51.90±1.01 | 0.38±0.02 | 1.66±0.57 | 7.48±0.69 |  |
| p.o. | 20 | 3.43±1.53 | 0.25±0.00 | 18.71±3.43 | 17.80±10.54 | 17.80±10.54 | 1.62±0.75 | 2.21±0.79 | ‐ | ‐ | ‐ | ‐ | 40.42±23.93 |
| s.c. | 20 | 7.65±1.16 | 0.33±0.14 | 11.64±6.89 | 18.00±3.64 | 18.00±3.64 | 3.50±1.22 | 5.19±2.09 | ‐ | ‐ | ‐ | ‐ | 40.89±8.28 |
| i.p. | 20 | 3.15±2.38 | 0.33±0.14 | 39.03±5.47 | 38.31±7.02 | 38.31±7.02 | 1.02±0.16 | 1.17±0.21 | ‐ | ‐ | ‐ | ‐ | 87.02±15.94 |

**Supplementary Table 9.** **Primers of biofilm-related genes used for qRT-PCR**

| Genes | Forward (3’→5’) | Reverse (3’→5’) | Product size (bp) |
| --- | --- | --- | --- |
| *agrB* | TACTAAGAAGAAGCCTATCC | TTAGTCCTCCTTTGAATAG | 184 |
| *icaR* | TTATCTAATACGCCTGAGGA | TCTTCCACTGCTCCAAAT | 206 |
| *eno* | AAACTGCCGTAGGTGACGAA | TGTTTCAACAGCATCTTCAGTACCTT | 68 |
| *cna* | CACAAGGCTATGGGTCAC | TTAAATGCTTTCCCGTTC | 142 |

**Supplementary Table 10. Primers of cytokines related genes used for qRT-PCR**

| Genes | Forward (3’→5’) | Reverse(3’→5’) | Product size (bp) |
| --- | --- | --- | --- |
| *actin* | ACATCCGTAAAGACCTCTATGCC | TACTCCTGCTTGCTGATCCAC | 223 |
| *IL-10* | GTTCCCCTACTGTCATCCCC | AGGCAGACAAACAATACACCA | 149 |
| *IL-6* | GACTTCCATCCAGTTGCCTT | ATGTGTAATTAAGCCTCCGACT | 150 |
| *TNFα* | AGCACAGAAAGCATGATCCG | CACCCCGAAGTTCAGTAGACA | 162 |
| *IL-1β* | TGAAATGCCACCTTTTGACAGT | TTCTCCACAGCCACAATGAGT | 189 |

**Synthesis of C218-0546.**

***Synthetic route:***

***General procedure for preparation of compound* *3***

To a solution of NaH (3.89 g, 97.3 mmol, 60.0% purity, 2.00 eq) in THF (60.0 mL) was added a solution of compound **2** (12.6 g, 97.3 mmol, 12.3 mL, 2.00 eq) in THF (20.0 mL) slowly at 0 °C under N_2_, the mixture was stirred at 0 °C for 0.5 h under N_2_, then compound **1** (10.0 g, 48.6 mmol, 6.37 mL, 1.00 eq) in THF (10.0 mL) was added. The mixture was stirred at 25 °C for 3 h under N_2_. TLC (Dichloromethane: Petroleum ether = 1: 1) showed compound **1** (R_f_ = 0.90) was consumed completely and two spots (R_f_ = 0.30, R_f_ = 0.40) were detected. The spot (R_f_ = 0.30) is the spot of compound **2**. The spot (R_f_ = 0.40) is the desired spot. 1 M HCl (20.0 mL) was added to reaction mixture slowly at 0 °C till pH = 2.00. The reaction mixture was extracted with ethyl acetate (50.0 mL). The organic phase was washed with NaCl saturated solution (20.0 mL), dried on Na_2_SO_4_ and evaporated to dryness. The residue was purified by column chromatography (SiO_2_, Petroleum ether: Dichloromethane = 30: 1 to 10: 1, R_f_ = 0.40). The compound **3** (8.00 g, 31.4 mmol, 64.5% yield) was obtained as a colorless oil.

***General procedure for preparation of compound* *5***

To a solution of compound **3** (8.00 g, 31.4 mmol, 1.00 eq) in EtOH (80.0 mL) was added compound **4** (4.87 g, 31.4 mmol, 1.00 eq) and phosphoric acid (6.16 g, 53.3 mmol, 3.66 mL, 85.0% purity, 1.70 eq), then stirred at 78 °C for 7 h. TLC (Petroleum ether: Ethyl acetate = 1: 1) showed compound **3** (R_f_ = 0.50) was consumed completely, 30% of compound **4** (R_f_ = 0.20) was remained, and the desired spot (R_f_ = 0.30) was detected. The reaction mixture was quenched by addition saturated NaHCO_3_ (100 mL) to pH = 7 - 8. Then the reaction mixture was concentrated under reduced pressure to remove EtOH. Then the mixture was extracted with ethyl acetate (100 mL * 2). The combined organic layers were washed with brine (100 mL), dried over Na_2_SO_4_, filtered and concentrated under reduced pressure to give a residue. The residue was purified by column chromatography (SiO_2_, Petroleum ether: Ethyl acetate = 30: 1 to 8: 1, R_f_ = 0.30). Compound **5** (7.00 g, 20.2 mmol, 64.4% yield) was obtained as a white solid confirmed by HNMR [DMSO-*d_6_*, 400 MHz, δ: 8.19 (s, 1H), 7.31-7.19 (m, 4H), 4.31 (q, *J* = 7.2 Hz, 2H), 3.89 (s, 2H), 2.45 (s, 3H), 1.31 (t, *J* = 6.8 Hz, 3H)] as follows:


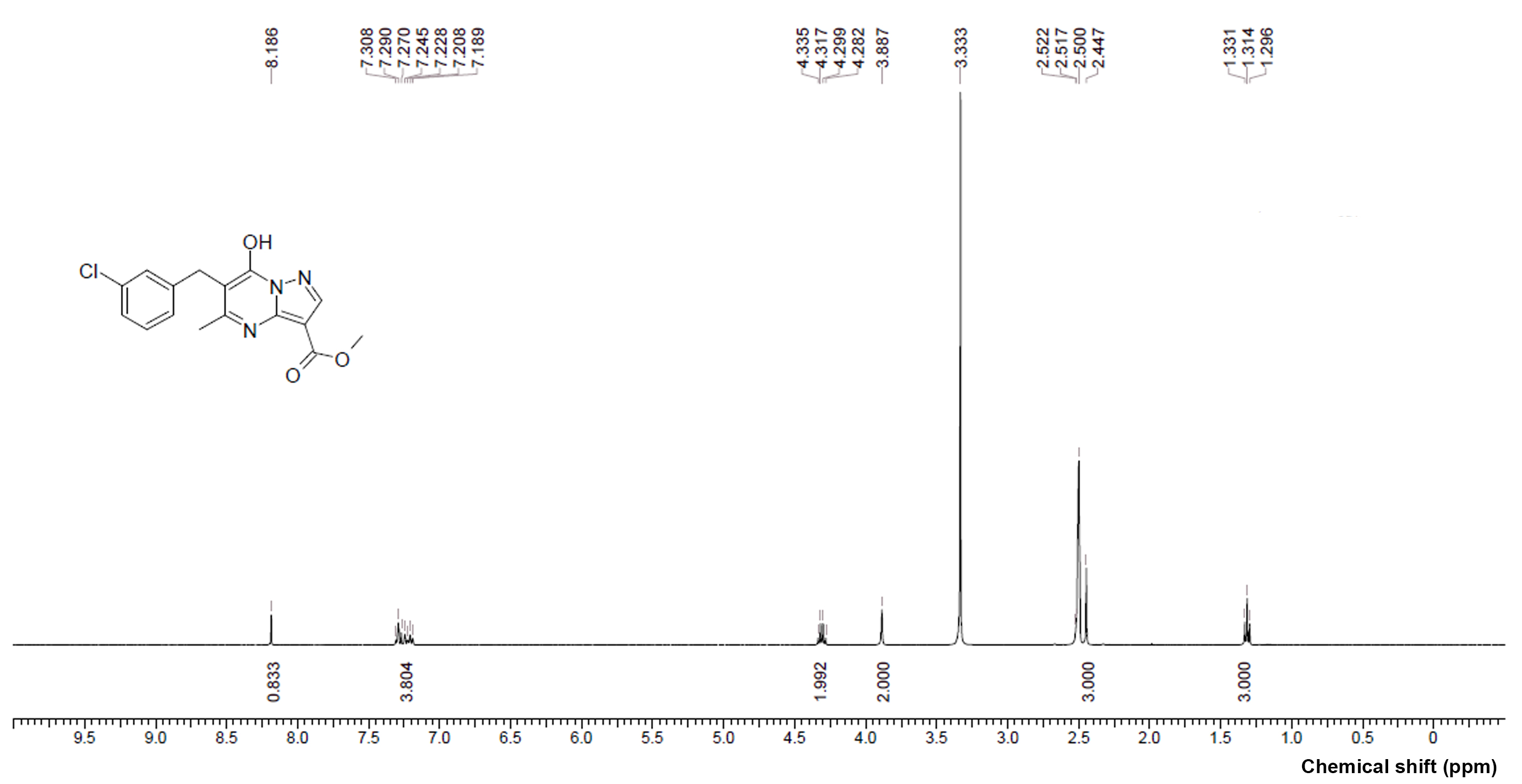


***General procedure for preparation of compound* *6***

To a solution of compound **5** (6.00 g, 17.3 mmol, 1.00 eq) in H_2_O (30.0 mL) and EtOH (30.0 mL) was added KOH (8.96 g, 159 mmol, 9.20 eq). The mixture was stirred at 78 °C for 48 h. TLC (Petroleum ether: Ethyl acetate = 0: 1) showed compound **5** (R_f_ = 0.60) was consumed completely and the desired spot (R_f_ = 0.10) was detected. The reaction mixture was quenched by addition 12 N HCl (11.0 mL) to pH = 3.00, then extracted with ethyl acetate (100 mL * 2). The combined organic layers were washed with brine (50.0 mL * 2), dried over Na_2_SO_4_, filtered, and evaporated in vacuo. The residue was used to the next step directly. Compound **6** (5.00 g, crude) was obtained as a white solid.

***General procedure for preparation of Target 2***

To a solution of compound **6** (5.00 g, 15.7 mmol, 1.00 eq) and compound **7** (2.90 g, 20.4 mmol, 2.48 mL, 1.30 eq) in DMF (50.0 mL) was added DIEA (5.08 g, 39.3 mmol, 6.85 mL, 2.50 eq) and T_3_P (7.51 g, 23.6 mmol, 7.02 mL, 1.50 eq), the reaction mixture was stirred at 25 °C for 3 h. LC-MS showed one main peak [R_t_ = 0.624 min, m/z = 440.9 (M+1)^+^] with desired mass was detected. The reaction mixture was quenched by 1N HCl (20.0 mL) to pH = 3.00. Then, the crude product was separated out. The crude product was purified by reversed-phase HPLC (0.10% FA condition) to obtain the **C218=0546** (1.00 g, 2.25 mmol, 14.3% yield, 99.4% purity) as light yellow solid confirmed by HNMR, LC-MS, and HPLC as follows:

LC-MS: R_t_ = 0.625 min, m/z = 440.9 (M+1)^+^

HPLC: purity: 99.4%, R_t_ = 3.284min

HNMR: DMSO-*d_6_*, 400 MHz. δ: 11.6 (s, 1H), 8.91 (q, *J* = 6.0 Hz, 5.6 Hz 1H), 8.44 (s, 1H), 7.46 - 7.44 (m, 2H), 7.31 -7.19 (m, 6H), 4.57-4.55 (m, 2H), 3.87 (s, 2H), 2.42 (s, 3H)


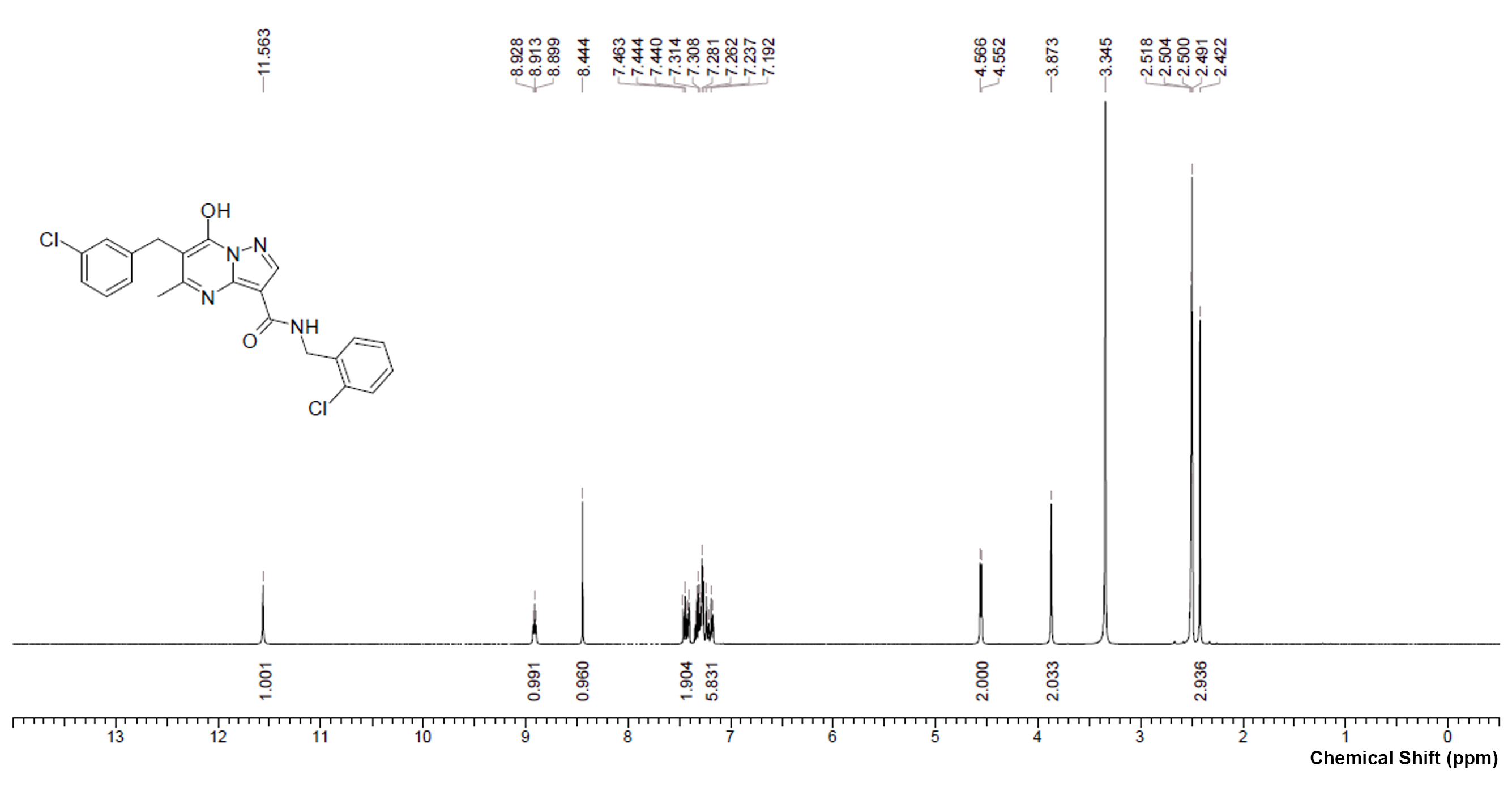


**Synthesis of STK848198.**

***Synthetic route:***

***General procedure for preparation of compound* *2***

To a solution of compound **1** (10.0 g, 55.6 mmol, 1.00 eq) in EtOH (100 mL) , was added N_2_H_4_•H_2_O (5.12 g, 100 mmol, 4.96 mL, 98% purity, 1.80 eq) and AcOH (10.4 g, 174 mmol, 10.0 mL, 3.14 eq), the mixture was stirred at 50 °C for 2 h. Then was added AcOH (20.9 g, 349 mmol, 20.0 mL, 6.27 eq), the mixture was stirred at 80 °C for 10 h. LCMS showed the compound **2** was consumed and one peak (R_t_ = 0.25 min, MS cal.: 193.0, MS observed: [M+H]^+^ = 193.9) with desired mass was detected. The reaction mixture was cooled to 25 °C. Then ice water (200 mL) was poured into the reaction mixture, and neutralized to pH = 9 by adding aqueous ammonia. The precipitate was filtered off, dried in air. Compound **2** was obtained as a yellow solid confirmed by HNMR [*δ:* 7.69, (s, 1H), 7.50 – 7.53 (m, 2H), 7.33 – 7.35 (m, 2H), 4.74 – 4.85 (m, 2H)] as follows:


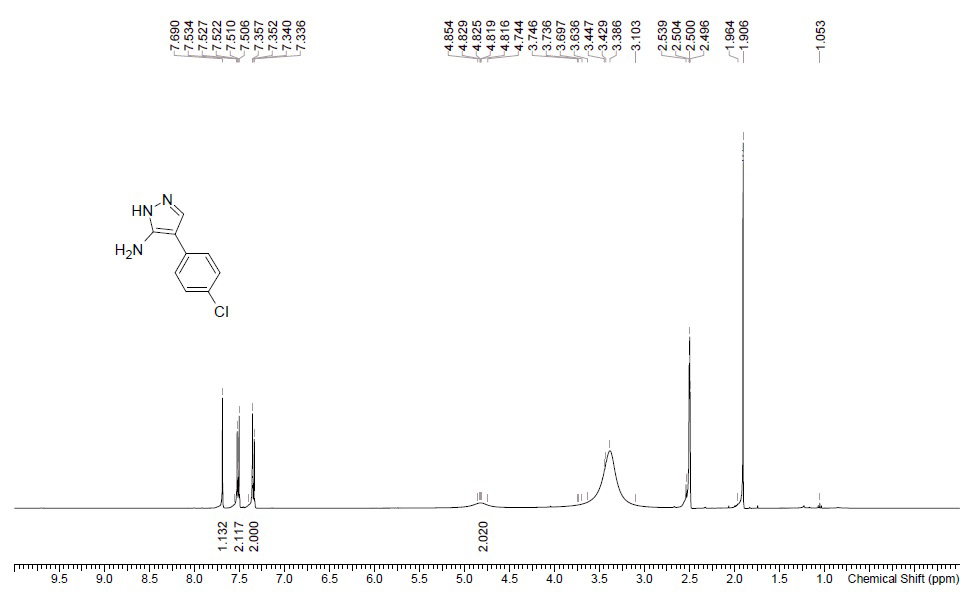
***General procedure for preparation of Target 1 (STK848198)***

To a solution of compound **2** (10.0 g, 50.5 mmol, 1.00 eq) in EtOH (100 mL), was added ethyl compound **3** (9.25 g, 41.9 mmol, 8.93 mL, 0.83 eq) and phosphoric acid (8.42 g, 85.9 mmol, 5.01 mL, 1.70 eq), the mixture was stirred at 78 °C for 12 h. LCMS showed the compound 2 was consumed and one peak (R_t_ = 0.43 min, MS cal.: 349.1, MS observed: [M+H]^+^ = 349.9) with desired mass was detected. The sediment was filtered out, washed with H_2_O (10.0 mL) five times, and concentrated under reduced pressure to give the product. STK848198 (Target 1, 3.00 g, 5.99 mmol, 99.7% yield, 99.7% purity) was obtained as a white solid confirmed by HNMR, LC-MS, and HPLC as follows:

LC-MS: R_t_ = 0.44 min, MS cal.: 349.1, MS observed: [M+H]^+^ = 349.9

HPLC: R_t_ = 2.79 min, purity: 99.7%

HNMR: *δ*: 11.7 (s, 1H), 8.13 (s, 1H), 7.58 – 7.59 (m, 2H), 7.48 – 7.51 (m, 2H), 7.25 – 7.26 (m, 4H), 7.16 – 7.24 (m, 1H), 3.87 (s, 2H), 2.37 (s, 3H)


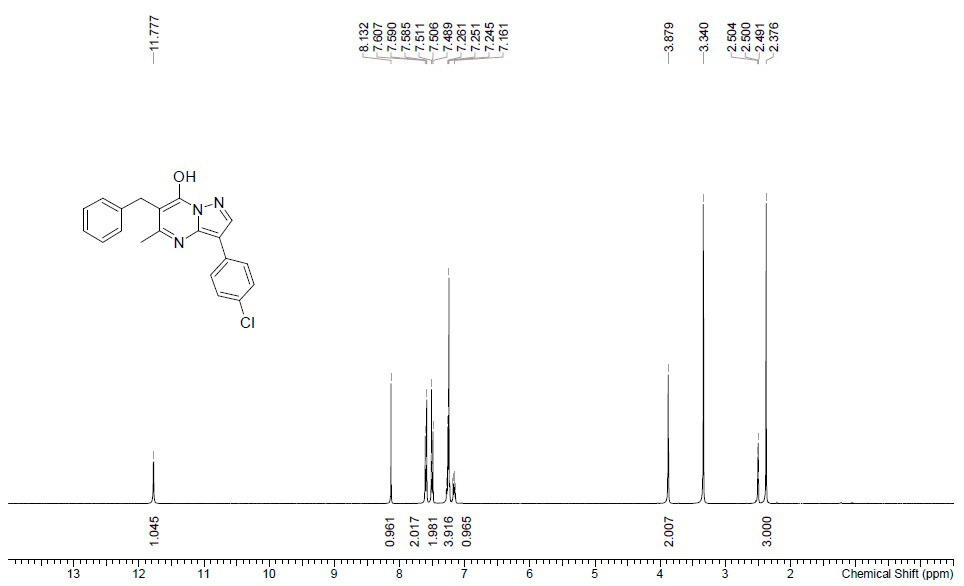


**REFERENCES**

1. CLSI. M100 performance standards for antimicrobial susceptibility testing. 33rd Edition. 2023.
2. She P, Yang Y, Li L, et al. Repurposing of the antimalarial agent tafenoquine to combat MRSA. *mSystems*. 2023;8(6):e0102623.
3. Kim W, Zhu W, Hendricks GL, et al. A new class of synthetic retinoid antibiotics effective against bacterial persisters. *Nature*. 2018;556(7699):103-107.
4. Ravindran R, Chakrapani G, Mitra K, Doble M. Inhibitory activity of traditional plants against Mycobacterium smegmatis and their action on Filamenting temperature sensitive mutant Z (FtsZ)-A cell division protein. *PLoS One*. 2020;15(5):e0232482.
5. Liu W, Gao R, Yang C, et al. ECM-mimetic immunomodulatory hydrogel for methicillin-resistant *Staphylococcus aureus*-infected chronic skin wound healing. *Sci Adv*. 2022;8(27):eabn7006.
6. O'Toole GA. Microtiter dish biofilm formation assay. *J Vis Exp*. 2011;(47):2437.
7. Xu L, She P, Chen L, et al. Repurposing Candesartan Cilexetil as Antibacterial Agent for MRSA Infection. *Front Microbiol*. 2021;12:688772. Published 2021 Sep 13.
8. Yu J, Jiang F, Zhang F, et al. Virtual Screening for Novel SarA Inhibitors to Prevent Biofilm Formation of *Staphylococcus aureus* in Prosthetic Joint Infections. *Front Microbiol*. 2020;11:587175.
9. Song YJ, Yu HH, Kim YJ, Lee NK, Paik HD. Anti-Biofilm Activity of Grapefruit Seed Extract against *Staphylococcus aureus* and *Escherichia coli*. *J Microbiol Biotechnol*. 2019;29(8):1177-1183.
10. da Silva FA, Medeiros SMFRDS, da Costa-Junior SD, et al. Antimicrobial Resistance Profile and Biofilm Production of Microorganisms Isolated from Oropharynx of *Rupornis magnirostris* (Gmelin, 1788) and *Caracara plancus* (Miller, 1777). *Vet Med Int*. 2020;2020:8888618.
11. Shukla SK, Rao TS. *Staphylococcus aureus* biofilm removal by targeting biofilm-associated extracellular proteins. *Indian J Med Res*. 2017;146(Supplement):S1-S8.
12. Sun Z, Sun Y, Li Y, et al. Identification of HeLa cell proteins that interact with Chlamydia trachomatis glycogen synthase using yeast two‑hybrid assays. *Mol Med Rep*. 2020;21(3):1572-1580.
13. Jiang F, Chen Y, Yu J, et al. Repurposed Fenoprofen Targeting SaeR Attenuates *Staphylococcus aureus* Virulence in Implant-Associated Infections. *ACS Cent Sci*. 2023;9(7):1354-1373. Published 2023 Jun 15.
14. Pisu D, Huang L, Grenier JK, Russell DG. Dual RNA-Seq of Mtb-Infected Macrophages In Vivo Reveals Ontologically Distinct Host-Pathogen Interactions. *Cell Rep*. 2020;30(2):335-350.e4.
15. She P, Li Z, Li Y, et al. Pixantrone Sensitizes Gram-Negative Pathogens to Rifampin. *Microbiol Spectr*. 2022;10(6):e0211422.
16. Gerits E, Defraine V, Vandamme K, et al. Repurposing Toremifene for Treatment of Oral Bacterial Infections. *Antimicrob Agents Chemother*. 2017;61(3):e01846-16.
17. Tan F, She P, Zhou L, et al. Bactericidal and Anti-biofilm Activity of the Retinoid Compound CD437 Against *Enterococcus faecalis*. *Front Microbiol*. 2019;10:2301.
18. Qiu Z, He Y, Ming H, Lei S, Leng Y, Xia ZY. Lipopolysaccharide (LPS) Aggravates High Glucose- and Hypoxia/Reoxygenation-Induced Injury through Activating ROS-Dependent NLRP3 Inflammasome-Mediated Pyroptosis in H9C2 Cardiomyocytes. *J Diabetes Res*. 2019;2019:8151836.
19. Kokot M, Weiss M, Zdovc I, et al. Amide containing NBTI antibacterials with reduced hERG inhibition, retained antimicrobial activity against gram-positive bacteria and in vivo efficacy. *Eur J Med Chem*. 2023;250:115160.
20. Yu Y, Zhao H, Lin J, et al. Repurposing Non-Antibiotic Drugs Auranofin and Pentamidine in Combination to Combat Multidrug-Resistant Gram-Negative Bacteria. *Int J Antimicrob Agents*. 2022;59(5):106582.
21. Song M, Liu Y, Li T, et al. Plant Natural Flavonoids Against Multidrug Resistant Pathogens. *Adv Sci (Weinh)*. 2021;8(15):e2100749.
22. Zheng J, Liu X, Xiong Y, et al. AMXT-1501 targets membrane phospholipids against Gram-positive and -negative multidrug-resistant bacteria. *Emerg Microbes Infect*. 2024;13(1):2321981.
23. Belley A, Harris R, Beveridge T, Parr T Jr, Moeck G. Ultrastructural effects of oritavancin on methicillin-resistant Staphylococcus aureus and vancomycin-resistant Enterococcus. *Antimicrob Agents Chemother*. 2009;53(2):800-804.
24. Zhang S, Qu X, Tang H, et al. Diclofenac Resensitizes Methicillin-Resistant *Staphylococcus aureus* to *β*-Lactams and Prevents Implant Infections. *Adv Sci (Weinh)*. 2021;8(13):2100681.
25. Liu Y, Jia Y, Yang K, et al. Metformin Restores Tetracyclines Susceptibility against Multidrug Resistant Bacteria. *Adv Sci (Weinh)*. 2020;7(12):1902227.
26. Farha MA, Verschoor CP, Bowdish D, Brown ED. Collapsing the proton motive force to identify synergistic combinations against Staphylococcus aureus. *Chem Biol*. 2013;20(9):1168-1178.
27. Li L, Zhou P, Wang Y, et al. Antimicrobial activity of cyanidin-3-O-glucoside-lauric acid ester against Staphylococcus aureus and Escherichia coli. *Food Chem*. 2022;383:132410.
28. Wang L, Yuan L, Zeng X, et al. A Multisite-Binding Switchable Fluorescent Probe for Monitoring Mitochondrial ATP Level Fluctuation in Live Cells. *Angew Chem Int Ed Engl*. 2016;55(5):1773-1776.
29. Jumper J, Evans R, Pritzel A, et al. Highly accurate protein structure prediction with AlphaFold. *Nature*. 2021;596(7873):583-589.
30. Hess B. P-LINCS:  A Parallel Linear Constraint Solver for Molecular Simulation. *J Chem Theory Comput*. 2008;4(1):116-122.
31. Martonák R, Laio A, Parrinello M. Predicting crystal structures: the Parrinello-Rahman method revisited. *Phys Rev Lett*. 2003;90(7):075503.
32. Van Der Spoel D, Lindahl E, Hess B, Groenhof G, Mark AE, Berendsen HJ. GROMACS: fast, flexible, and free. *J Comput Chem*. 2005;26(16):1701-1718.
33. Sanner MF. Python: a programming language for software integration and development. *J Mol Graph Model*. 1999;17(1):57-61.
34. Morris GM, Huey R, Lindstrom W, et al. AutoDock4 and AutoDockTools4: Automated docking with selective receptor flexibility. *J Comput Chem*.
35. Pietrocola G, Pellegrini A, Alfeo MJ, Marchese L, Foster TJ, Speziale P. The iron-regulated surface determinant B (IsdB) protein from *Staphylococcus aureus* acts as a receptor for the host protein vitronectin. *J Biol Chem*. 2020;295(29):10008-10022.
36. Espinoza J, Urzúa A, Sanhueza L, et al. Essential Oil, Extracts, and Sesquiterpenes Obtained From the Heartwood of *Pilgerodendron uviferum* Act as Potential Inhibitors of the *Staphylococcus aureus* NorA Multidrug Efflux Pump. *Front Microbiol*. 2019;10:337.
37. Ekins S, Lingerfelt MA, Comer JE, et al. Efficacy of Tilorone Dihydrochloride against Ebola Virus Infection. *Antimicrob Agents Chemother*. 2018;62(2):e01711-17.
38. Brazel EB, Tan A, Neville SL, et al. Dysregulation of Streptococcus pneumoniae zinc homeostasis breaks ampicillin resistance in a pneumonia infection model. *Cell Rep*. 2022;38(2):110202.
39. Pengfei S, Yaqian L, Lanlan X, et al. L007-0069 kills Staphylococcus aureus in high resistant phenotypes. *Cell Mol Life Sci*. 2022;79(11):552.
40. Yang Y, Sun N, Lv J, et al. Environmentally realistic dose of tire-derived metabolite 6PPD-Q exposure causes intestinal jejunum and ileum damage in mice via cannabinoid receptor-activated inflammation. *Sci Total Environ*. 2024;918:170679.
41. Li Z, She P, Liu Y, et al. Triple combination of SPR741, clarithromycin, and erythromycin against Acinetobacter baumannii and its tolerant phenotype. *J Appl Microbiol*. 2023;134(1):lxac023.
42. Song M, Liu Y, Huang X, et al. A broad-spectrum antibiotic adjuvant reverses multidrug-resistant Gram-negative pathogens. *Nat Microbiol*. 2020;5(8):1040-1050.
43. Hussain Z, Pengfei S, Yimin L, et al. Study on antibacterial effect of halicin (SU3327) against Enterococcus faecalis and Enterococcus faecium. *Pathog Dis*. 2022;80(1):ftac037.
